# Rate Dependence on Inductive and Resonance Effects for the Organocatalyzed Enantioselective Conjugate Addition of Alkenyl and Alkynyl Boronic Acids to β-Indolyl Enones and β-Pyrrolyl Enones

**DOI:** 10.3390/molecules26061615

**Published:** 2021-03-14

**Authors:** Amy Boylan, Thien S. Nguyen, Brian J. Lundy, Jian-Yuan Li, Ravikrishna Vallakati, Sasha Sundstrom, Jeremy A. May

**Affiliations:** 1 Department of Chemistry, University of Houston, 3585 Cullen Blvd., Fleming Building 112, Houston, TX 77204-5003, USA; amy.boylan1@gmail.com (A.B.); thiensnguyen@gmail.com (T.S.N.); brianjlundy@yahoo.com (B.J.L.); Jian-Yuan.Li@bcm.edu (J.-Y.L.); vallark@gmail.com (R.V.); sasha.oley@gmail.com (S.S.); 2 Graduate School of Energy, Environment, Water and Sustainability (EEWS), Korea Advanced Institute of Science and Technology (KAIST), 291 Daehak-ro, Yuseong-gu, Daejeon 34141, Korea; 3 Baker Hughes, 17021 Aldine Westfield Rd, Houston, TX 77073, USA; 4 Baylor College of Medicine, One Baylor Plaza, Houston, TX 77030, USA; 5 Vallark Pharma Pvt. Ltd., Genome Valley, Turkapally, Hyderabad 500078, India; 6 Department of Chemistry and Biochemistry, Baylor Sciences Bldg. D.208, One Bear Place #97348, Waco, TX 76798, USA

**Keywords:** enantioselective conjugate addition, heterocycles, pyrrole, indole

## Abstract

Two key factors bear on reaction rates for the conjugate addition of alkenyl boronic acids to heteroaryl-appended enones: the proximity of inductively electron-withdrawing heteroatoms to the site of bond formation and the resonance contribution of available heteroatom lone pairs to stabilize the developing positive charge at the enone β-position. For the former, the closer the heteroatom is to the enone β-carbon, the faster the reaction. For the latter, greater resonance stabilization of the benzylic cationic charge accelerates the reaction. Thus, reaction rates are increased by the closer proximity of inductive electron-withdrawing elements, but if resonance effects are involved, then increased rates are observed with electron-donating ability. Evidence for these trends in isomeric substrates is presented, and the application of these insights has allowed for reaction conditions that provide improved reactivity with previously problematic substrates.

## 1. Introduction

**Figure 1:**
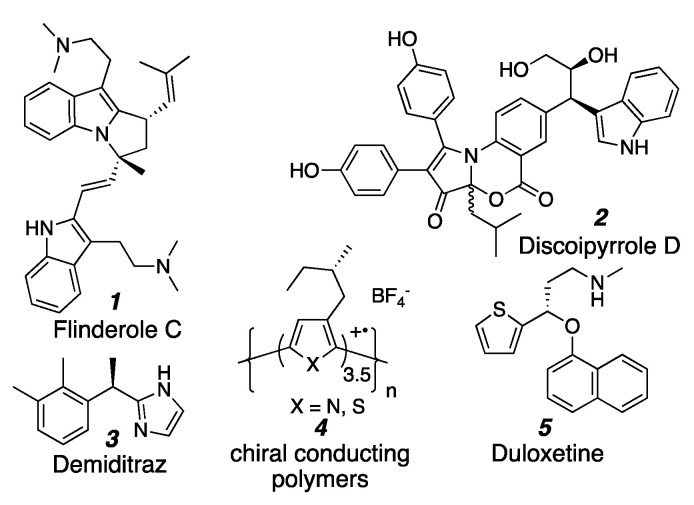
Examples of chiral heteroaromatic compounds.

Heteroaromatics routinely appear as key pharmacophores in small molecule drugs [1,2,3,4,5], as common motifs in natural products [6,7,8], and as important functional groups in materials [9]. The ability to synthesize heteroaromatic systems attached to stereocenters is becoming increasingly important, especially as greater three-dimensionality in compounds is increasingly desired (Figure 1) [10,11,12,13,14,15,16].

**Figure 2:**
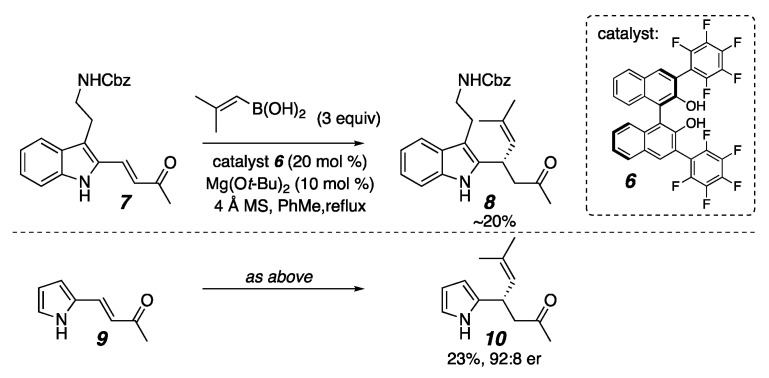
Problematic substrates for organocatalyzed conjugate addition.

Concordantly, many recent reports have described efforts to develop new strategies and catalysts to synthesize heteroaryl-bearing stereocenters with absolute stereocontrol. To cite limited examples, transition metal-mediated couplings [17,18,19,20,21,22,23,24,25,26], Petasis-like reactions [27,28,29], C—H functionalizations [30,31,32,33,34,35], Friedel–Crafts reactions [36,37,38,39,40,41,42,43,44,45,46,47,48,49,50,51,52,53,54,55,56,57,58,59,60,61,62,63,64,65,66,67,68,69,70,71,72,73,74,75,76,77,78,79,80,81,82,83,84,85,86], and conjugate additions have provided significant advances [87,88,89,90,91,92,93,94,95,96,97,98,99,100,101,102,103,104,105,106,107]. We have contributed to this area by demonstrating that α-chiral heterocycles can be synthesized through 3,3′-(bisperfluoroaryl)-BINOL (**6**)-catalyzed conjugate addition of aryl, alkenyl, and alkynyl boronic acids and trifluoroborate salts to β-heteroaryl-appended enones and enals [87,88,89,90]. When heteroaryl trifluoroborate salts are used as nucleophiles, bis-heteroaryl stereocenters are formed [90]. We have consistently encountered two problematic but synthetically important substrates, however: β-(2-indolyl)-enones and β-(pyrrolyl)-enones (Figure 2). This was especially vexing as the α-chiral indole **8** was a proposed intermediate for an enantioselective synthesis of flinderole C (**1**). This report describes why these substrates are problematic, how resonance effects impact the reaction rate and success, and how to increase these substrates’ reactivity.

The use of boronate esters and boronic acids as nucleophiles in catalyzed conjugate additions to enones dates to Suzuki [99,108,109,110,111,112]. More recent efforts have led to transition metal-catalyzed and organocatalyzed enantioselective versions of this reaction. For the latter cases, examples exist of BINOL-based ligands pioneered by Chong [113,114,115,116], 〈-hydroxy acids reported by Sugiura [117,118,119,120], and thiourea catalysts from Takemoto [121]. Those reports, however, primarily dealt with aryl-substituted stereocenter formation, and so they offered little information on how to address heterocycle incorporation and the problematic 2-indole and 2-pyrrole substrates.

## 2. Results

**Figure 3:**
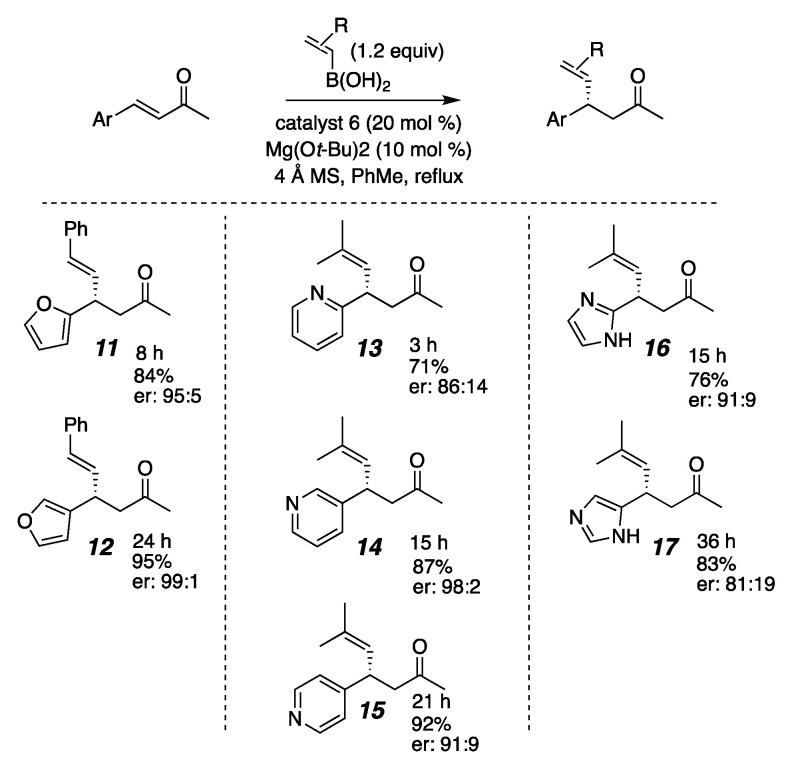
Reaction times for series of heteroaromatic substrates.

**Figure 4:**
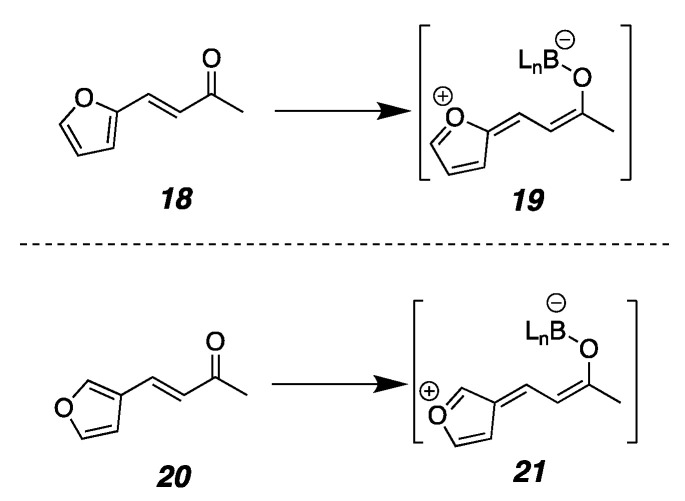
Resonance stabilization of Lewis acid/base interactions.

In looking at data collected from the many heteroaromatic substrates that we had examined, patterns emerged for how the point of enone attachment on furan, pyridine, and imidazole rings affected the reaction rate (Figure 3). In the furanyl enone **11**, where the enone is attached at the 2-position, the conjugate addition reaction occurs in only 8 h, while its counterpart, **12**, which is attached at the 3-position, is not complete until 24 h. Pellegrinet and Goodman established that the initial step in the organocatalyzed conjugate addition mechanism is the formation of a discreet Lewis acid/base adduct between the enone and the catalyst ligated boronate ester [118,122]. One may draw equally viable resonance structures that stabilize the putative Lewis acid/base interaction for the 2- and 3-furan isomers (**19** and **21**, Figure 4) [122,123]. Since the difference in reaction rate was not readily correlated to resonance stabilization, we considered the possibility that proximity to the furan oxygen played a role. Similarly, in β-pyridyl-enones, the reactivity does not correlate to any typical resonance effects in that the 2-pyridine and 4-pyridine substrates do not exhibit similar rates. Rather, the trend still appears correlated with the proximity of the heteroatom to the reacting site, with 2-pyridyl **13** being formed within 3 h and 4-pyridyl **15** taking 21 h for complete reaction, which again implicates inductive electronic effects. Recruitment of the Lewis acidic nucleophile by the pyridyl nitrogen in a similar manner to Takemoto’s work [121] cannot be fully ruled out, either. For the imidazole substrates **16** and **17**, similar resonance structures may be drawn for either isomer as seen for the furans, so resonance effects did not explain the reactivity difference. Again, having more nitrogens closer to the site of reactivity as seen in the 2-imidizole isomer gave a faster reaction than for the 4-imidazole isomer. Taken together, these substrates suggest that proximity to the inductively electron-withdrawing heteroatom in a heteroaromatic substituent accelerates this conjugate addition. They also exhibited high levels of enantioselectivity.

**Figure 5:**
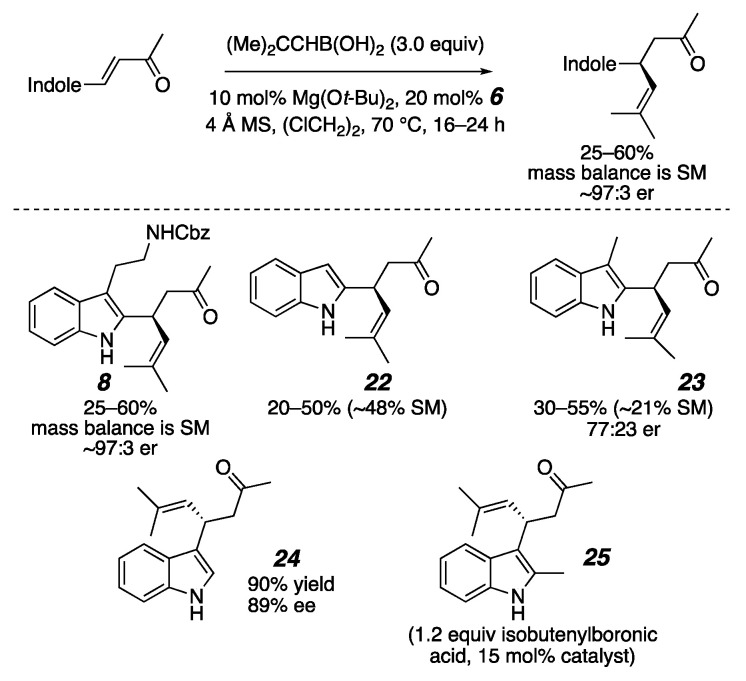
Indole control experiments.

**Figure 6:**
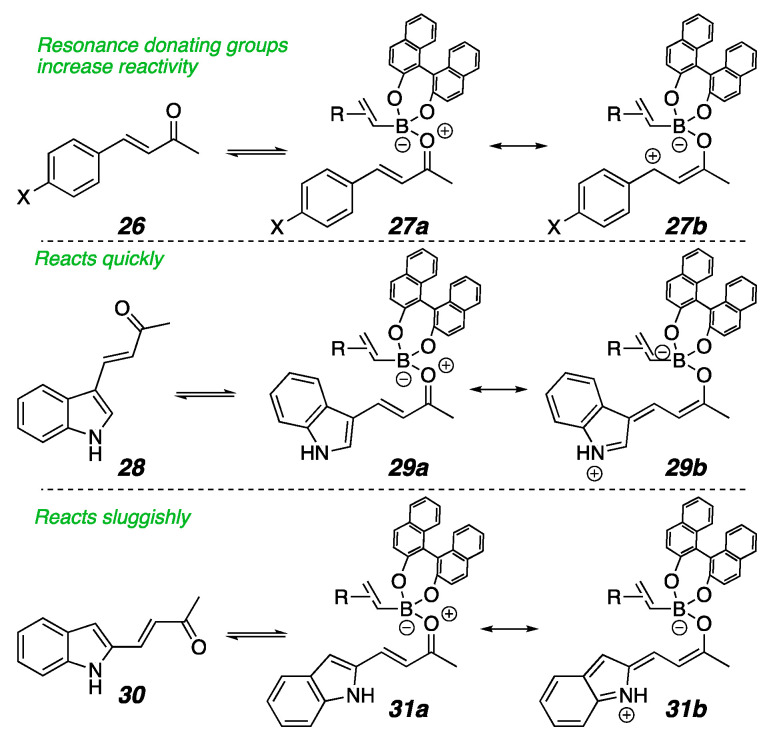
Stabilization of the zwitterionic intermediate.

However, the trend described in the previous paragraph is opposite to that for the indole-substituted enones, where the high-performing 3-indole substrates bear the nitrogen further from the enone β-carbon than the poor-performing 2-indolo-enones (Figure 5). Moreover, inconsistent and unpredictable yields of **8** were routinely obtained. An early thought for the discrepancy was that the enone **7** has substitution at both the 2- and 3-positions, which would increase steric repulsion at the reactive site. However, control experiments with **22**–**25** in Figure 6 dispelled that notion, as the inferior reactivity was clearly due to the indole position of substitution and therefore more likely to be due to the system’s electronics. We reasoned that for these substrates, resonance effects might have played a larger role than the inductive effects seen in Figure 3. A relationship study for resonance effects and reaction rates using a Hammett plot analysis of aryl-substituted enones shed some mechanistic insight on what may have been occurring for the indole substrates [124]. In that study, a clear Hammett parameter correlation was seen for electron-donating substituents on the β-aromatic ring accelerating the reaction, which suggested that the stabilization of the benzylic cationic charge in **27b** increased the reaction rate, likely because the formation of zwitterionic intermediate **27a** is necessary for the reaction (Figure 6). While the resonance structures for charge stabilization for the 2/3-furan and 2/4-imidazole substrates were similar, those for the indoles **28** and **30** are quite different in relative energy because of the additional fused aromatic ring. The 3-indoloenone can stabilize the charge with the resonance structure **29b**, which maintains the aromaticity in the fused benzene ring, but similar resonance stabilization in the 2-indoloenone **31b** would require the loss of aromaticity. This phenomenon is the reason behind the well-established Friedel–Crafts reactivity patterns seen for indoles, where electrophilic substitution preferentially occurs at the 3-position, and would also make the enone 28 more Lewis basic than 30. To compensate for this energy difference, we proposed that we needed to make the 2-indoles more electron-rich for the key Lewis acid/base interaction illustrated in **31a**.

**Figure 7:**
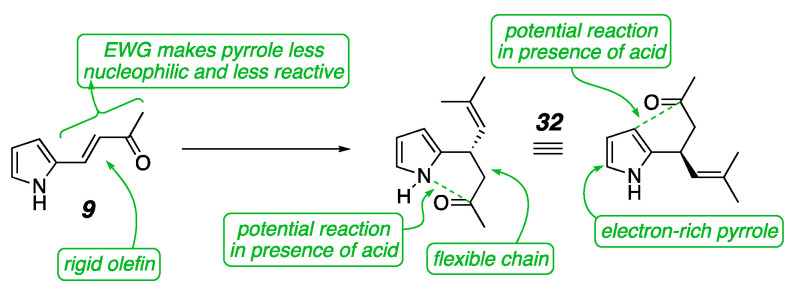
Pyrrole problems.

We also looked more closely at the problems with pyrrole substrates. Control experiments showed that the issues stemmed both from high reactivity found in the starting materials and even greater instability of the products. As evidence of the latter, when pure ketone **9** was reintroduced to the reaction conditions, it readily decomposed. When the starting material alone was stirred with the base and no other reactants, it also formed a new unstable product which could not be isolated or fully characterized. After the conjugate addition, the pyrrole in **32** is electron-rich and nucleophilic, has no protecting group, and bears no steric blocking groups (Figure 7). Various side reactions were consequently seen, such as a pyrrole nitrogen attack on the ketone carbonyl, forming a cyclized product that could be observed in the NMR of the crude reaction mixture but was not stable enough to isolate [19,20]. The Lewis acidic catalyst complex was thought to be promoting the side reactions, and so a less electron-deficient BINOL catalyst was sought as well as milder reaction conditions.

**Figure 8:**
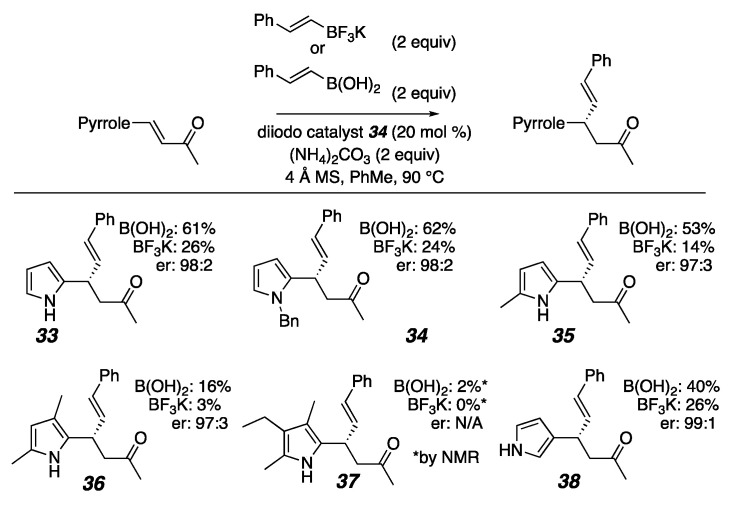
Pyrrole substrates (NMR spectra and HPLC data in Supplementary Materials).

Initially, we thought that a base additive could deprotonate the hydrogen of the pyrrole or indole substituent, at least partially, which would result in a greater electron density in the ring [124,125]. That electron density would then in turn be donated to activate the enone as in Figure 8. As a result, we evaluated a variety of bases to test this theory (Table 1). Note that in the original conditions reported for boronic acid nucleophiles (see Figure 2), Mg(O*t*-Bu)_2_ is used only in sufficient quantities to deprotonate the catalyst. Moreover, *t*-BuOH replicated its effects, suggesting that their function was most likely to serve as a proton transfer agent. The Mg salt was usually slightly better, so metal coordination or pH adjustment may play a role in those conditions. Regardless, Mg(O*t*-Bu)_2_ did not provide useful reactivity for 2-indole substrates (**22** and **23**, Figure 5). The carbonate bases generally outperformed the other bases in 24 h of reaction (entries 2–6). More soluble bases, such as Cs_2_CO_3_ and Na_2_CO_3_, produced less of the conjugate addition product compared to a less soluble base, such as (NH_4_)_2_CO_3_ (entries **3**–**6**). It usually took several hours for the (NH_4_)_2_CO_3_ to dissolve in the solution. Bases that were stronger also resulted in a significant decrease in yield (entry **8**, **9**, and **17**). Overall, the use of a full equivalent of (NH_4_)_2_CO_3_ and 3,3’-diiodo-BINOL (**34**, Table 1) as a catalyst significantly addressed the deficient reactivity of the indole substrates and the hyper-reactivity of the pyrrole compounds.

Since (NH_4_)_2_CO_3_ showed much better outcomes for the pyrrole substrate, we tested those conditions on a variety of indoles and pyrroles, which provided a variety of interesting results (Figure 8). We found that when we protected the unsubstituted 2-pyrrolyl-enone **34**, we obtained nearly identical results to the unprotected version (**33**). This outcome invalidated our initial hypothesis for the role of a base in deprotonating an indole or pyrrole nitrogen. We also found that as more substituents were incorporated onto the pyrrole, the desired reactivity faltered (**35**–**37**). In a control experiment, when the purified products were reintroduced into the reaction conditions, they decomposed. Another indication of how reactive these substituted pyrrole substrates are is that they decompose in ambient lighting more quickly than the unsubstituted starting material **9**. Due to this high reactivity, the most substituted products are not stable enough to be isolated in useful yield. Another possibility for decreased conjugate addition yields could be a result of sterics. As more substituents are added to the ring, especially at the 3-position of the pyrrole, the sterics of these substituents could be causing allylic strain, inhibiting the conjugate addition reaction, and allowing more time for side reactions and decomposition to occur. A similar trend with pyrroles has been observed by the Qiu group [124].

**Figure 9:**
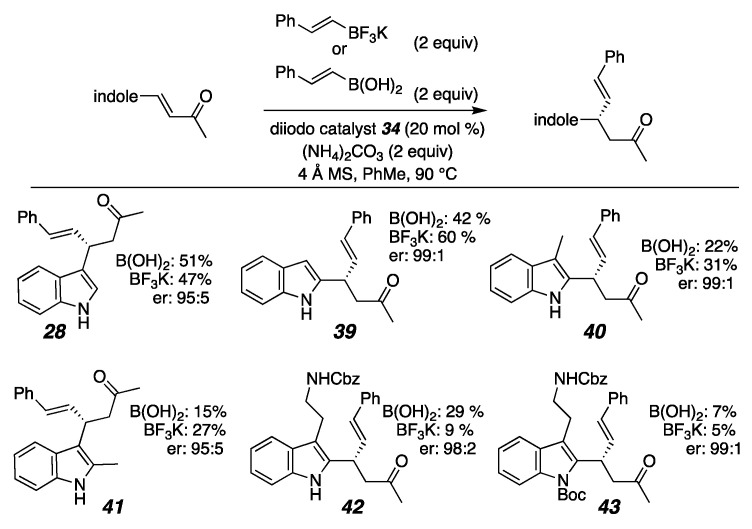
Indole substrates (NMR spectra and HPLC data in Supplementary Materials).

**Figure 10:**
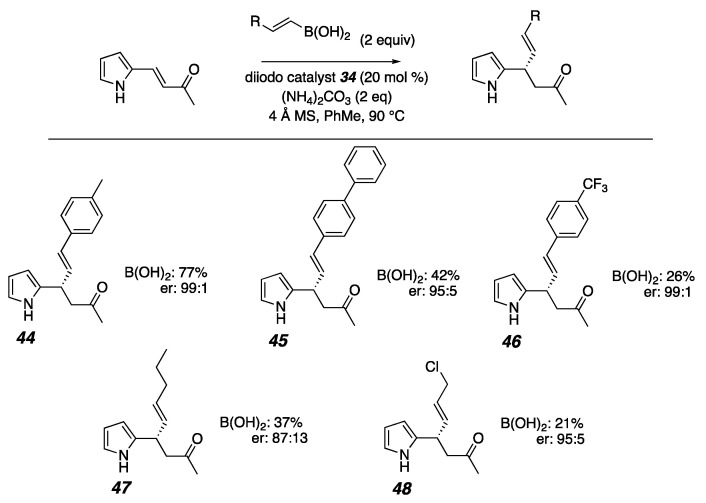
2-pyrrolyl-enone with boronic acids (NMR spectra and HPLC data in Supplementary Materials).

A control experiment of stirring the pyrrolyl-enone with just (NH_4_)_2_CO_3_ in toluene without light at 90 °C without a catalyst or organoboron nucleophile resulted in an unwanted reaction that produced a side product too unstable to isolate. This indicated to us that the base has both an advantageous effect on the conjugate addition and an adverse effect on the starting material stability, creating a conflicted system. Typically, trifluoroborate salts work better in conjugate addition reactions because of their prolonged stability over their boronic acid counterparts [124]. Interestingly, though, in all of the pyrrole substrates (Figure 10) and some of the indole substrates (Figure 9), the boronic acids resulted in higher yields than their trifluoroborate counterparts. These findings led us to believe the base, (NH_4_)_2_CO_3_, could also be helping to promote boroxine formation from the boronic acid or maintain a favorable pKa for the conjugate addition reaction to occur. For the indole substrates in Figure 9, (NH_4_)_2_CO_3_ also improved the yield for the conjugate addition product. The unsubstituted indoles **28** and **39** resulted in moderate yields with both the boronic acid and trifluoroborate salt. The mono-substituted indoles **40** and **41** resulted in better yields when the trifluoroborate salt nucleophile was used compared to when the boronic acid was used. Both the pyrrole and indole products were formed with excellent enantioselectivity (Figure 8 and Figure 9).

A variety of alkenyl boronic acids also show compatibility with these reaction conditions with the problematic 2-pyrroyl-enone (Figure 10). In most cases, the products that were formed in fair to good yields show excellent enantioselectivity (**44**–**48)**.

## 3. Conclusions

Two problematic series of substrates, β-(2-indole)-enones and β-(2-pyrrole)-enones, were thoroughly examined in the enantioselective organocatalyzed conjugate addition of alkenyl boronic acids or trifluoroborates. Analysis of isomer-related reaction rate trends showed that (1) the proximity of a heteroatom to the enone β-carbon was favorable to the reaction rate and (2) increased resonance electron donation also increased the reaction rate. The use of (NH_4_)_2_CO_3_ promoted the conjugate addition reaction better than Mg(O*t*-Bu)_2_ or other additives. The use of a less electron-deficient catalyst in conjunction with the new base minimized side product formation and provided the most advantageous environment for the conjugate addition to sensitive substrates to date.

## 4. Materials and Methods

### 4.1. Materials

Commercially available compounds were purchased from Aldrich (Burlington, MA, USA), Acros (Geel, Belgium), Alfa Aesar (Ward Hill, MA, USA), Ark Pharm (Chicago, IL, USA), and Combi-block (San Diego, CA, USA) and were used without further purification.

### 4.2. General Considerations

All reactions were carried out in flame- or oven-dried glassware. THF, toluene, and CH_2_Cl_2_ were purged with argon and dried over activated alumina columns. Flash chromatography was performed on 60 Å silica gel (EMD Chemicals Inc (St. Louis, MO, USA)). Preparative plate chromatography was performed on EMD silica gel plates, 60 Å, with UV-254 indicator. Chemical names were generated using Cambridge Soft ChemBioDraw Ultra 12.0. Analysis by HPLC was performed on a Shimadzu Prominence LC (LC-20AB) equipped with an SPD-20A UV-Vis detector and a Chiralpak or Chiralcel (250 × 4.6 mm) column (see below for column details). Analytical thin layer chromatography was performed on EMD silica gel/TLC plates with a fluorescent detector at 254 nm. The ^1^H and ^13^C-NMR spectra were recorded on a JEOL ECA-600, JEOL ECA-500, or ECX-400P spectrometer using residual solvent peak as an internal standard (CDCl_3_: 7.26 ppm for ^1^H-NMR and 77.0 ppm for ^13^C-NMR; C6D6: 7.15 ppm for ^1^H-NMR and 128.6 ppm for ^13^C-NMR).

HPLC Columns for Separation of Enantiomers:

Chiralpak AY-3: Amylose tris-(5-chloro-2-methylphenylcarbamate) coated on 3 μm silica gel; Chiralpak AD-H: Amylose tris-(3,5-dimethylphenylcarbamate) coated on 5 μm silica gel; Chiralpak ID: Amylose tris-(3-chlorophenylcarbamate) immobilized on 5 μm silica gel; Chiralcel OJ-H: Cellulose tris-(4-methylbenzoate) coated on 5 μm silica gel; Chiralcel OD-H: Cellulose tris-(3,5-dimethylphenylcarbamate) coated on 5 μm silica gel; Chiralpak AS-H: Amylose tris-[(*S*)-α-methylbenzylcarbamate) coated on 5 μm silica gel. 

(*E*)-4-(furan-2-yl)but-3-en-2-one(**11**) [48], (*E*)-4-(furan-3-yl)but-3-en-2-one(**12**) [48], (*E*)-4-(pyridin-2-yl)but-3-en-2-one (**13**) [48], (*S*)-6-methyl-4-(pyridin-3-yl)hept-5-en-2-one(**14**) [48], (*S*)-6-methyl-4-(pyridin-4-yl)hept-5-en-2-one (**15**) [48], (*S*)-4-(1H-imidazol-2-yl)-6-methylhept-5-en-2-one (**16**) [48], (*S*)-4-(1H-imidazol-5-yl)-6-methylhept-5-en-2-one (**17**) [48],(*E*)-4-(1H-pyrrol-2- yl)but-3-en-2-one [48],(*E*)-4-(1-benzyl-1H-pyrrol-2-yl)but-3-en-2-one [89],(*E*)-4-(1H-indol-3-yl)but-3-en-2-one and Benzyl (*E*)-(2-(2-(3-oxobut-1-en-1-yl)-1H-indol-3-yl)ethyl)carbamate [87], benzyl (*S*,*E*)-(2-(2-(5-oxo-1-phenylhex-1-en-3-yl)-1H-indol-3-yl)ethyl)carbamate [87], tert-butyl (*S*,*E*)-3-(2-(((benzyloxy)carbonyl)amino)ethyl)-2-(5-oxo-1-phenylhex-1-en-3-yl)-1H-indole-1- carboxylate [87], 1,2 (*E*)-4-(1H-indol-2-yl)but-3-en-2-one (**30**) [87], (*E*)-4-(1H-pyrrol-3-yl)but-3-en-2-one [87], (*S*)-4-(1H-indol-2-yl)-6-methylhept-5-en-2-one (**22**) [87], (*S*)-6-methyl-4-(1H-pyrrol-2-yl)hept-5-en-2-one (**10**) [87], (*S*)-4-(1H-indol-2-yl)-6-methylhept-5-en-2-one, (*S*,*E*)-4-(1H-indol-3-yl)-6-phenylhex-5-en-2-one(**67**) [87], benzyl (*S*,*E*)-(2-(2-(5-oxo-1-phenylhex-1-en-3-yl)-1H-indol-3-yl)ethyl)carbamate (**42**) [48], tert-butyl (*S*,*E*)-3-(2-(((benzyloxy)carbonyl)amino)ethyl)-2-(5-oxo-1-phenylhex-1-en-3-yl)-1H-indole-1- carboxylate(**43**) [87], (S,E)-6-phenyl-4-(1H-pyrrol-2-yl)hex-5-en-2-one (**33**) [89], (*S*,*E*)-4-(1H-indol-2-yl)-6-phenylhex-5-en-2-one (**39**) [48], and (*E*)-4-(1-benzyl-1H-pyrrol-2-yl)but-3-en-2-one [48] were synthesized following literature procedures.

### 4.3. General Procedure for the Synthesis of Starting Materials (Enone)

Carboxaldehyde (2 mmol), 1-(triphenylphosphoranylidene)-2-propanone (1.2 equiv, 764 mg), and toluene (4 mL) were added to a flask equipped with a stir bar and a condenser. The reaction mixture was refluxed for 10 h. After completion, the reaction mixture was concentrated via rotary evaporation. The crude mixture was purified via flash column chromatography with an appropriate eluent on silica gel. 

#### 4.3.1. (*E*)-4-(5-methyl-1H-pyrrol-2-yl)but-3-en-2-one

See the general procedure for enone formation above; in addition, the reaction was shielded from light by covering the reaction and product with aluminum foil. The product will decompose in prolonged exposure to light. An amount of 1 g of 5-methyl-1H-pyrrole-2-carbaldehyde was used. The crude reaction mixture was purified via flash column chromatography with a 10–20% gradient of ethyl acetate in hexanes as eluent on silica gel. Yield: 56%

^1^H-NMR (500 MHz, chloroform-D) δ 8.45 (s, 1H), 7.51 (d, *J* = 16.0 Hz, 1H), 7.08 (s, 1H), 6.82 (s, 1H), 6.48−6.43 (m, 2H), 2.32 (s, 3H)

^1^H-NMR (600 MHz, Benzene-D6) δ 7.18 (s, 2H), 6.39 (s, 1H), 6.34 (s, 1H), 5.93 (s, 1H), 1.96 (s, 3H), 1.33(s, 3H).

^13^C-NMR (126 MHz, chloroform-d) δ 198.6, 133.6, 121.4, 119.1, 117.6 113.3, 110.8, 110.0, 31.3, 13.9

IR(neat): 3283, 1613, 1560, 1477, 1423, 1358, 1263, 959, 764, 700, 489 cm^−1^

HRMS-ESI *m*/*z* Calculated for C_9_H_11_NO [M + H]^+^ 150.0913, found 150.0916.

#### 4.3.2. (*E*)-4-(3,5-dimethyl-1H-pyrrol-2-yl)but-3-en-2-one

See the general procedure for enone formation above; in addition, the reaction was shielded from light by covering the reaction and product with aluminum foil. The product will decompose in prolonged exposure to light. An amount of 1 g of 3,5-dimethyl-1*H*-pyrrole-2-carbaldehyde was used. The crude reaction mixture was purified via flash column chromatography with a 10–20% gradient of ethyl acetate in hexanes as eluent on silica gel. Yield: 70%

^1^H-NMR (400 MHz, chloroform-d) δ 8.53 (s, 1H), 7.41 (d, *J* = 15.6 Hz, 1H), 6.16 (d, *J* = 16.0 Hz, 1H), 5.89 (d, *J* = 17.8 Hz, 1H), 2.29 (d, *J* = 11.2 Hz, 6H), 2.18 (s, 3H), 1.80 (s, 2H)

^13^C-NMR (101 MHz, chloroform-D) δ 198.4, 131.1, 130.6, 127.2, 112.3, 111.2, 31.1, 13.8, 13.5, 11.5

IR (neat): 3292, 3246, 1600, 1559, 1433, 1358, 1258, 953, 839, 785, 711, 668 cm^−1^

HRMS-ESI *m*/*z* Calculated for C_10_H_13_NO [M + Na]^+^ 290.1515, found 290.1525.

#### 4.3.3. (*E*)-4-(4-ethyl-3,5-dimethyl-1H-pyrrol-2-yl)but-3-en-2-one 

See the general procedure for enone formation above; in addition, the reaction was shielded from light by covering the reaction and product with aluminum foil. The product will decompose in prolonged exposure to light. An amount of 1 g of 4-ethyl-3,5-dimethyl-1*H*-pyrrole-2-carbaldehyde was used. The crude reaction mixture was purified via flash column chromatography with a 10–20% gradient of ethyl acetate in hexanes as eluent on silica gel. Yield: 55%

IR(neat): 3254, 2961, 2912, 2855, 1612, 1570, 1444, 1253, 950 cm^−1^.

HRMS-ESI *m*/*z* Calculated for C_12_H_17_NO [M + H]^+^ 192.1383, found 192.1386.

#### 4.3.4. (*E*)-4-(3-methyl-1H-indol-2-yl)but-3-en-2-one

A mixture of 3-methyl-1H-indole-2-carbaldehyde (0.1 mmol), but-3-yn-2-one (0.15 mmol), and Sc(OTf)_3_ (10 mol%) in MeCN (0.5 mL) was stirred at 21 °C for the appropriate time. After complete conversion, as indicated by TLC, the reaction mixture was diluted with H_2_O and extracted with EtOAc. The combined organic layers were dried over MgSO_4_, concentrated in vacuo, and purified by column chromatography with a 5–30% gradient of ethyl acetate in hexanes as eluent on silica gel.

^1^H-NMR (500 MHz, chloroform-d): δ 8.60 (brs, NH), 7.66 (d, *J* = 16.5 Hz, 1H), 7.58 (d, *J* = 8.0 Hz, 1H), 7.35 (d, *J* = 8.0 Hz, 1H) 7.29–7.25 (m, 1H), 7.11 (t, *J* = 8.0 Hz, 1H), 6.50 (d, *J* = 16.5 Hz, 1H), 2.44 (s, 3H), 2.41 (s, 3H).

^13^C-NMR (125 MHz, chloroform-d): δ 198.1, 137.7, 131.1, 129.9, 129.0, 125.4, 122.8, 119.9, 119.9, 119.9, 111.1, 27.4, 9.0.

IR(neat): 3299, 1634, 1598, 1257, 1235, 953, 747, 622, 459 cm^−1^.

HRMS-ESI *m*/*z* Calculated for C_13_H_13_NO [M + H]^+^ 200.1070, found 200.1072.

### 4.4. Procedure for Boronic Acid Synthesis: 2-Methylprop-1-Enylboronic Acid

LiCl (1.008 g, 24 mmol, 1.2 equiv) was added to a 250 mL flask and the flask was flame dried under high vacuum. The flask was then back filled with argon. Then, 0.5 M 2-Methyl-1-propenyl magnesium bromide in THF (40 mL, 20 mmol, 1.0 equiv) and Et2O (50 mL) were added. The solution was cooled to −78 °C. Trimethyl borate (2.5 mL, 22 mmol, 1.1 equiv) was added dropwise and the reaction was allowed to slowly warm to room temperature and stir overnight. The next day, it was quenched with 1 M HCl (30 mL) until the reaction mixture became clear and then stirred for 1 h. It was then extracted with Et_2_O (3 times) and washed with sat. aqueous NaHCO_3_ and brine solution. The organic layer was dried with Na_2_SO_4_ and then concentrated via rotary evaporation. The crude solid was purified via column chromatography with a 20–30% gradient of ethyl acetate in hexanes as eluent on silica gel to afford a white solid (1.105 g, 11.06 mmol, 55% yield). All spectral properties were identical to those reported in the literature.

### 4.5. General Procedure for 1,4-Conjugate Addition (Mg(t-BuO)_2_ as Additive)

Here, 4 Å powdered molecular sieves (100 mg) were added to a flask equipped with a stir bar and a condenser and the flask was flame dried under high vacuum. The flask was then back filled with argon. The heterocycle-appended enone (0.2 mmol, 1.0 equiv), Mg(*t*-BuO)_2_ (3.4 mg, 0.02 mmol, 0.1 equiv), boronic acid (3 equiv), and BINOL catalyst (0.04 mmol, 0.2 equiv) were then added. Freshly dried toluene (4 mL) was added and the reaction was heated to reflux in a 70–78 °C oil bath and allowed to stir at this temperature (see each product for specific reaction times). After completion, methanol was added and the reaction mixture was concentrated via rotary evaporation. The crude reaction mixture was then dry loaded onto silica gel and purified via flash column chromatography on silica gel with appropriate eluents. All spectral properties.

(*S*)-6-methyl-4-(1H-pyrrol-2-yl)hept-5-en-2-one (**10**)

^1^H-NMR (400 MHz, chloroform-D): δ 8.41 (brs, NH), 6.66 (dd, *J* = 4.0, 2.4 Hz, 1H), 6.11 (dd, *J* = 5.6, 2.8 Hz, 1H), 5.89–5.88 (m, 1H), 5.27–5.24 (m, 1H), 4.11 (ddd, *J* = 9.5, 6.8, 6.8 Hz, 1H), 2.89 (dd, *J* = 17.2, 7.6 Hz, 1H), 2.73 (dd, *J* = 17.2, 6.0 Hz, 1H), 2.14 (s, 3H), 1.75 (d, *J* = 1.6 Hz, 3H), 1.70 (d, *J* = 1.6 Hz, 3H).

^13^C-NMR (125 MHz, chloroform-D): δ 208.8, 134.5, 133.4, 125.0, 116.5, 108.0, 103.8, 50.0, 32.4, 30.6, 25.8,18.0.

### 4.6. General Procedure for 1,4-Conjugate Addition ((NH_4_)_2_CO_3_ as an Additive)

Here, 4 Å powdered molecular sieves (100 mg) were added to a flask equipped with a stir bar and a condenser and the flask was flame dried under high vacuum. The flask was then back filled with argon. The heterocycle-appended enone (0.2 mmol, 1.0 equiv), (NH_4_)_2_CO_3_ (38 mg, 0.4 mmol, 2.0 equiv), boronic acid (2 equiv), and BINOL catalyst (0.04 mmol, 0.2 equiv) were then added. Freshly dried toluene (4 mL) was added and the reaction was heated to 90 °C in an oil bath and allowed to stir at this temperature for 24 h. After completion, the reaction mixture was concentrated via rotary evaporation. The crude reaction mixture was then dry loaded onto silica gel and purified via flash column chromatography on silica gel with eluents of 10–30% ethyl acetate in hexanes.

(*S*,*E*)-4-(3-methyl-1H-indol-2-yl)-6-phenylhex-5-en-2-one(**40**)

See the general procedure for enone formation above. The crude reaction mixture was purified via flash column chromatography with a 10–30% gradient of ethyl acetate in hexanes as eluent on silica gel.

^1^H-NMR (500 MHz, chloroform-d) δ 8.31 (s, 1H), 7.67 (d, *J* = 16.0 Hz, 1H), 7.59 (d, *J* = 8.0 Hz, 1H), 7.35–7.27 (m, 2H), 7.12 (t, *J* = 7.4 Hz, 1H), 6.45 (d, *J* = 16.0 Hz, 1H), 3.50 (s, 1H), 2.45 (s, 3H), 2.41 (s, 3H), 1.36–1.25 (m, 1H)

^13^C-NMR (101 MHz, chloroform-d) δ 184.5, 138.9, 128.6, 128.4, 127.6, 126.5, 125.8, 124.9, 124.3, 122.9, 121.2, 110.6, 108.7, 74.5, 35.5, 19.0, 13.3

IR(neat): 3090, 3070, 3035, 1477, 1034, 669 cm^−1^.

HRMS-ESI *m*/*z* Calculated for C_21_H_21_NO [M + Na]^+^ 326.1515, found 326.1518.

(*S*,*E*)-4-(2-methyl-1H-indol-3-yl)-6-phenylhex-5-en-2-one(**41**)

See the general procedure for enone formation above. The crude reaction mixture was purified via flash column chromatography with a 10–30% gradient of ethyl acetate in hexanes as eluent on silica gel.

^1^H-NMR (600 MHz, Benzene-D) δ 7.60 (d, *J* = 7.6 Hz, 1H), 7.17–7.11 (m, 5H), 7.01 (t, *J* = 7.2 Hz, 3H), 6.94 (t, *J* = 7.2 Hz, 1H), 6.50 (dd, *J* = 15.8, 5.5 Hz, 1H), 6.40 (d, *J* = 15.8 Hz, 1H), 6.36 (s, 1H), 4.40 (q, *J* = 6.4 Hz, 1H), 2.87 (q, *J* = 8.5 Hz, 1H), 2.57 (dd, *J* = 16.5, 5.5 Hz, 1H), 2.06 (s, 3H), 1.44 (s, 3H)

^13^C-NMR (101 MHz, chloroform-D) δ 208.0, 135.5, 132.2, 129.3, 128.5, 128.4, 127.1, 126.3, 121.0, 119.3, 119.2, 110.6, 48.2, 35.1, 30.9, 12.2.

IR(neat): 3091, 3071, 3035, 1477, 1035, 668 cm^−1^.

HRMS-ESI m/z Calculated for C_21_H_21_NO [M + H]^+^ 326.1515, found 326.1520.

(*S*,*E*)-4-(5-methyl-1H-pyrrol-2-yl)-6-phenylhex-5-en-2-one (**35**)

See the general procedure for enone formation above. The crude reaction mixture was purified via flash column chromatography with a 10–30% gradient of ethyl acetate in hexanes as eluent on silica gel.

^1^H-NMR (400 MHz, chloroform-d) δ 8.04 (s, 1H), 7.36–7.28 (m, 4H), 7.23 (d, *J* = 6.6 Hz, 1H), 6.48 (d, *J* = 16.2 Hz, 1H), 6.30 (q, *J* = 7.9 Hz, 1H), 5.78 (d, *J* = 11.4 Hz, 2H), 4.05–4.01 (m, 1H), 3.03–2.89 (m, 2H), 2.22 (s, 3H), 2.17 (s, 3H)

^13^C-NMR (101 MHz, chloroform-d) δ 208.2, 131.8, 130.8, 130.5, 128.7, 128.4, 127.6, 127.3, 126.4, 105.7, 104.7, 48.9, 37.0, 30.8, 13.1.

IR(neat): 3090, 3070, 3035, 1959, 1814, 1477, 1034, 668 cm^−1^.

HRMS-ESI m/z Calculated for C_17_H_19_NO [M + Na]^+^ 276.1359, found 276.1358.

(*S*,*E*)-4-(3,5-dimethyl-1H-pyrrol-2-yl)-6-phenylhex-5-en-2-one (**36**)

See the general procedure for enone formation above. The crude reaction mixture was purified via flash column chromatography with a 10–30% gradient of ethyl acetate in hexanes as eluent on silica gel.

^1^H-NMR (400 MHz, chloroform-d) δ 7.86 (s, 1H), 7.36–7.28 (m, 5H), 7.22–7.18 (m, 1H), 6.36 (d, *J* = 2.3 Hz, 1H), 5.65 (s, 1H), 4.07 (dd, *J* = 11.5, 6.3 Hz, 1H), 2.94 (d, *J* = 6.4 Hz, 2H), 2.19 (s, 3H), 2.13 (s, 3H), 2.02 (s, 3H).

^13^C-NMR (101 MHz, chloroform-d) δ 208.2, 137.1, 130.8, 129.7, 128.6, 127.4, 126.3, 126.0, 114.6, 108.2, 48.5, 35.5, 30.7, 29.8, 13.1, 11.2

IR(neat): 3090, 3070, 3035, 1959, 1814, 1477, 1034, 668 cm^−1^.

HRMS-ESI m/z Calculated for C_18_H_21_NO [M + Na]^+^ 290.1515, found 290.1525.

(*S*,*E*)-4-(4-ethyl-3,5-dimethyl-1H-pyrrol-2-yl)-6-phenylhex-5-en-2-one(**37**)

See the general procedure for enone formation above. The crude reaction mixture could not be purified, so an NMR standard, 4-methylnitrobenzoate, was used to obtain the yield. All reactants were added to the reaction mixture along with 0.1 mmol of 4-methylnitrobenzoate. The aryl peaks for the 4-methylnitrobenzoate were compared with the typical quartet around 4.0–4.4 ppm, indicating that the beta-bond formed during the conjugate addition reaction.

(*S*,*E*)-6-phenyl-4-(1H-pyrrol-3-yl)hex-5-en-2-one (**38**)

See the general procedure for enone formation above. The crude reaction mixture was purified via flash column chromatography with a 10–30% gradient of ethyl acetate in hexanes as eluent on silica gel.

^1^H-NMR (500 MHz, chloroform-d) δ 8.08 (s, 1H), 7.34 (d, *J* = 7.4 Hz, 2H), 7.28 (d, *J* = 7.4 Hz, 2H), 7.18 (t, *J* = 7.2 Hz, 1H), 6.75 (s, 1H), 6.62 (s, 1H), 6.42 (d, *J* = 15.5 Hz, 1H), 6.30 (q, *J* = 7.8 Hz, 1H), 6.13 (s, 1H), 4.03 (q, *J* = 7.3 Hz, 1H), 2.86 (qd, *J* = 15.8, 7.2 Hz, 2H), 2.12 (s, 3H).

^13^C-NMR (151 MHz, Benzene-d) δ 204.4, 138.7, 137.3, 133.6, 132.1, 129.9, 128.5, 128.4, 128.3, 128.0, 127.9, 127.8, 127.6, 127.3, 127.2, 126.6, 126.3, 121.7, 107.6, 106.1, 50.1, 48.3, 35.3, 29.7

IR(neat): 3090, 3080, 3035, 1959, 1814, 1477, 1034, 668 cm^−1^.

HRMS-ESI m/z Calculated for C_16_H_17_NO [M + Na]^+^ 262.1202, found 262.1208.

(*S*,*E*)-4-(1H-pyrrol-2-yl)-6-(p-tolyl)hex-5-en-2-one (**44**)

See the general procedure for enone formation above. The crude reaction mixture was purified via flash column chromatography with a 10–20% gradient of ethyl acetate in hexanes as eluent on silica gel.

^1^H-NMR (600 MHz, Benzene-d) δ 7.52 (s, 1H), 7.29–7.22 (m, 2H), 7.12 (t, *J* = 7.6 Hz, 2H), 7.06–7.03 (m, 1H), 6.38–6.23 (m, 2H), 5.98 (d, *J* = 2.7 Hz, 2H), 3.95 (q, *J* = 6.9 Hz, 1H), 2.53–2.34 (m, 2H), 1.93 (t, *J* = 15.5 Hz, 3H), 1.58 (s, 3H)

^13^C-NMR (151 MHz, Benzene-d) δ 206.0, 134.7, 132.9, 130.2, 129.3, 128.3, 127.9, 127.8, 127.6, 126.4, 116.9, 108.2, 104.9, 48.6, 36.8

IR(neat): 3380, 3022, 2920, 1706, 1512, 1358, 967, 794, 720 cm^−1^.

HRMS-ESI *m*/*z* Calculated for C_17_H_19_NO [M + Na]^+^ 276.1359, found 276.1361.

(*S*,*E*)-6-([1,1′-biphenyl]-4-yl)-4-(1H-pyrrol-2-yl)hex-5-en-2-one (**45**)

See the general procedure for enone formation above. The crude reaction mixture was purified via flash column chromatography with a 10–20% gradient of ethyl acetate in hexanes as eluent on silica gel.

^1^H-NMR (600 MHz, Benzene-d) δ 7.72 (s, 1H), 7.47 (dd, *J* = 23.7, 7.9 Hz, 4H), 7.27 (d, *J* = 8.2 Hz, 2H), 7.23 (t, *J* = 7.6 Hz, 2H), 7.14 (d, *J* = 6.9 Hz, 1H), 6.41–6.24 (m, 4H), 6.09 (s, 1H), 3.97 (q, *J* = 6.9 Hz, 1H), 2.48 (q, *J* = 8.2 Hz, 1H), 2.35 (dd, *J* = 17.2, 6.2 Hz, 1H), 1.56 (s, 3H)

^13^C-NMR (151 MHz, chloroform-d) 208.2, 130.7, 128.9, 127.4, 127.0, 126.8, 117.3, 108.2, 104.7, 100.0, 77.3, 77.1, 76.9, 74.8, 49.0, 36.9, 11.3

IR(neat): 3334, 3027, 2925, 1697, 964, 720, 691 cm^−1^.

HRMS-ESI m/z Calculated for C_22_H_21_NO [M + Na]^+^ 338.1515, found 338.1520.

(*S*,*E*)-4-(1H-pyrrol-2-yl)-6-(4-(trifluoromethyl)phenyl)hex-5-en-2-one (**46**)

See the general procedure for enone formation above. The crude reaction mixture was purified via flash column chromatography with a 10–20% gradient of ethyl acetate in hexanes as eluent on silica gel.

^1^H-NMR (600 MHz, Benzene-d) δ 7.72 (s, 1H), 7.47 (dd, *J* = 23.7, 7.9 Hz, 4H), 7.27 (d, *J* = 8.2 Hz, 2H), 7.23 (t, *J* = 7.6 Hz, 2H), 7.14 (d, *J* = 6.9 Hz, 1H), 6.41–6.24 (m, 4H), 6.09 (s, 1H), 3.97 (q, *J* = 6.9 Hz, 1H), 2.48 (q, *J* = 8.2 Hz, 1H), 2.35 (dd, *J* = 17.2, 6.2 Hz, 1H), 1.56 (s, 3H)

^13^C-NMR (151 MHz, chloroform-d) δ 208.0, 133.4, 132.7, 129.4, 126.5, 125.6, 117.4, 108.2, 104.9, 77.3, 77.1, 76.9, 76.8, 48.9, 36.8, 30.7

IR(neat): 3407, 2924, 1704, 1363, 1325, 1222, 529 cm^−1^

HRMS-ESI m/z Calculated for C_17_H_16_F_3_NO [M + H]^+^ 308.1257, found 308.1254.

(*S*,*E*)-4-(1H-pyrrol-2-yl)non-5-en-2-one (**47**)

See the general procedure for enone formation above. The crude reaction mixture was purified via flash column chromatography with a 10–20% gradient of ethyl acetate in hexanes as eluent on silica gel.

^1^H-NMR (600 MHz, chloroform-d) δ 8.38 (s, 1H), 6.68 (q, *J* = 2.3 Hz, 1H), 6.11 (q, *J* = 3.0 Hz, 1H), 5.88 (s, 1H), 5.55–5.54 (m, 2H), 3.87 (q, *J* = 6.6 Hz, 1H), 2.90–2.72 (m, 2H), 2.16 (d, *J* = 20.6 Hz, 3H), 2.02–1.99 (m, 2H), 1.42–1.36 (m, 2H), 0.90–0.87 (m, 3H)

^13^C-NMR (151 MHz, chloroform-d) δ 208.7, 134.1, 131.8, 130.6, 116.9, 108.0, 104.3, 77.3, 77.1, 76.9, 49.4, 36.7, 34.6, 30.7, 22.6, 13.8

IR(neat): 3378, 2957, 2927, 1704, 1357, 966, 712 cm^−1^.

HRMS-ESI *m*/*z* Calculated for C_13_H_19_NO [M + Na]^+^ 228.1359, found 228.1359.

## Figures and Tables

**Table 1:**
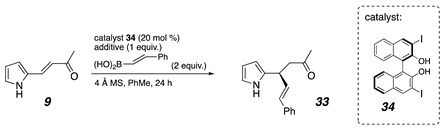
Optimizations of additives with 2-pyrrolyl enone. 0.20 mmol 27 with 0.02 mmol catalyst (20 mol %), 0.4 mmol of boronic acid, 0.4 mmol additive, 100 mg 4 Å MS, and 4 mL PhMe, stirred at reflux for 24 h.

Entry	Additive	Yield
1	Mg(Ot-Bu)_2_	10%
2	(NH_4_)_2_CO_3_	64%
3	K_2_CO_3_	53%
4	Cs_2_CO_3_	35%
5	Li_2_CO_3_	29%
6	Na_2_CO_3_	4%
7	K_3_PO_4_	34%
8	NaHMDS	13%
9	LiHMDS	6%
10	KOH	5%
11	NaOH	4%
12	KO*t*-Bu	4%
13	NaO*t*-Bu	3%
14	LiO*t*-Bu	0%
15	NH_4_Cl	2%
16	NH_4_HSO_4_	0%
17	DBU	3%

## Data Availability

The data presented in this study are available in this article and in the Supplementary Information file.

## Supplementary Materials

The following are available online.
