# Supplementary material for: Rate Dependence on Inductive and Resonance Effects for the Organocatalyzed Enantioselective Conjugate Addition of Alkenyl and Alkynyl Boronic Acids to β-Indolyl Enones and β-Pyrrolyl Enones
Source: Molecules. 2021 Mar 14;26(6):1615. doi: 10.3390/molecules26061615 (PMC8000498; doi:10.3390/molecules26061615)
Supplement: Supplementary file 1 [file molecules-26-01615-s001.pdf]

Rate-Dependence on Inductive and Resonance Effects for the Organocatalyzed Enantioselective Conjugate Addition of Alkenyl and Alkynyl Boronic Acids to β-Indolyl Enones and β-Pyrrolyl Enones.

Amy Boylan[1], Thien N. Nguyen[1,2], Brian J. Lundy[1,3], Jian-Yuan Li[1,4], Ravikrishna Vallakati[1,5], Sasha Sundstom[1,6], and Jeremy A. May[1,*]

Department of Chemistry, University of Houston, 3585 Cullen Blvd, Fleming Bldg. Room 112, TX 77204-5003, United States

TABLE OF CONTENTS

Spectra of Starting Materials..SI-1

Spectra of Products..SI-2

HPLC data...SI-3

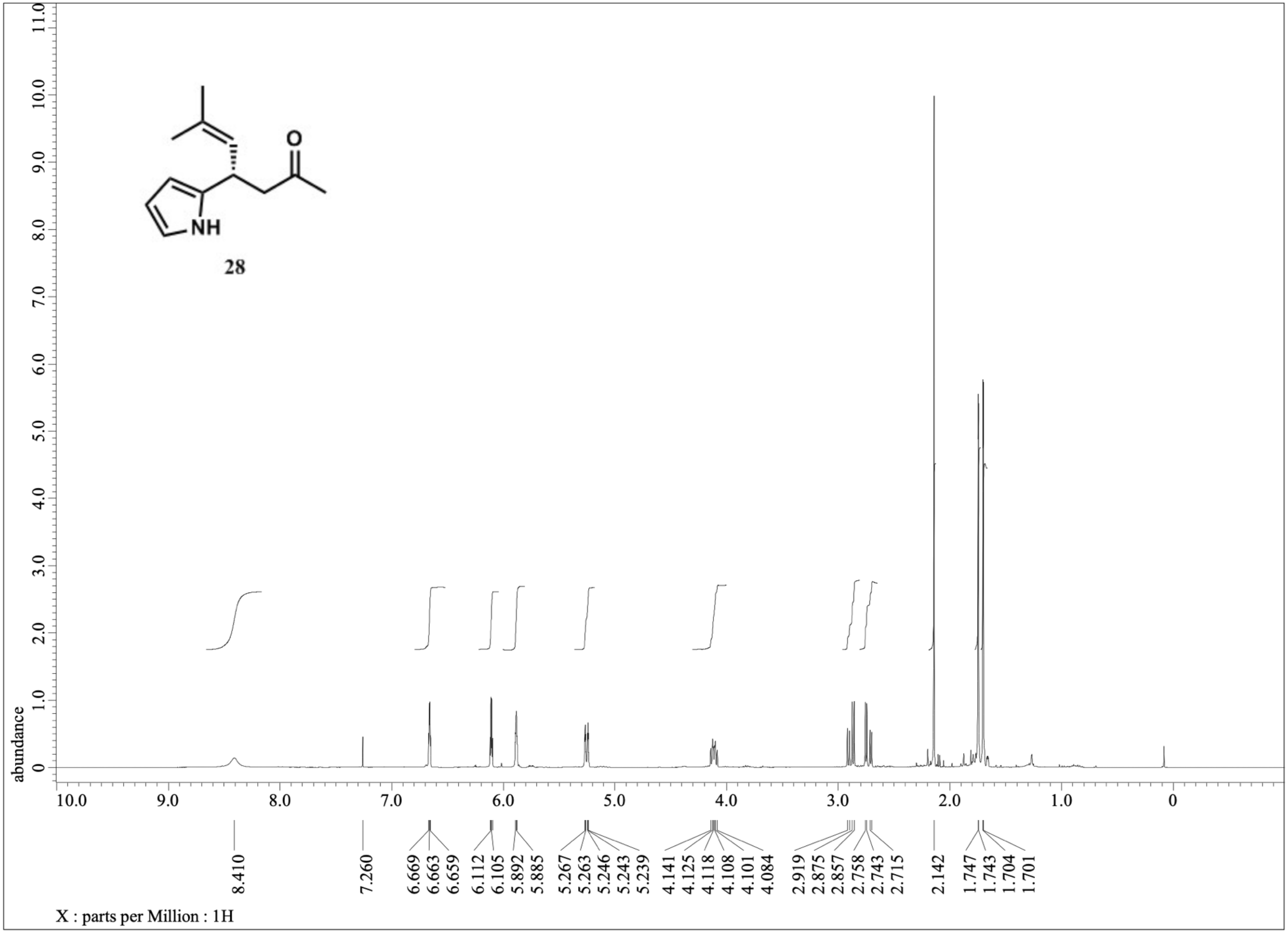
NH
O
28
abundance
11.0
10.0
9.0
8.0
7.0
6.0
5.0
4.0
3.0
2.0
1.0
0
10.0
9.0
8.0
7.0
6.0
5.0
4.0
3.0
2.0
1.0
0
8.410
7.260
6.669
6.663
6.659
6.112
6.105
5.892
5.885
5.267
5.263
5.246
5.243
5.239
4.141
4.125
4.118
4.108
4.101
4.084
2.919
2.875
2.857
2.758
2.743
2.715
2.142
1.747
1.743
1.704
1.701
X : parts per Million : 1H

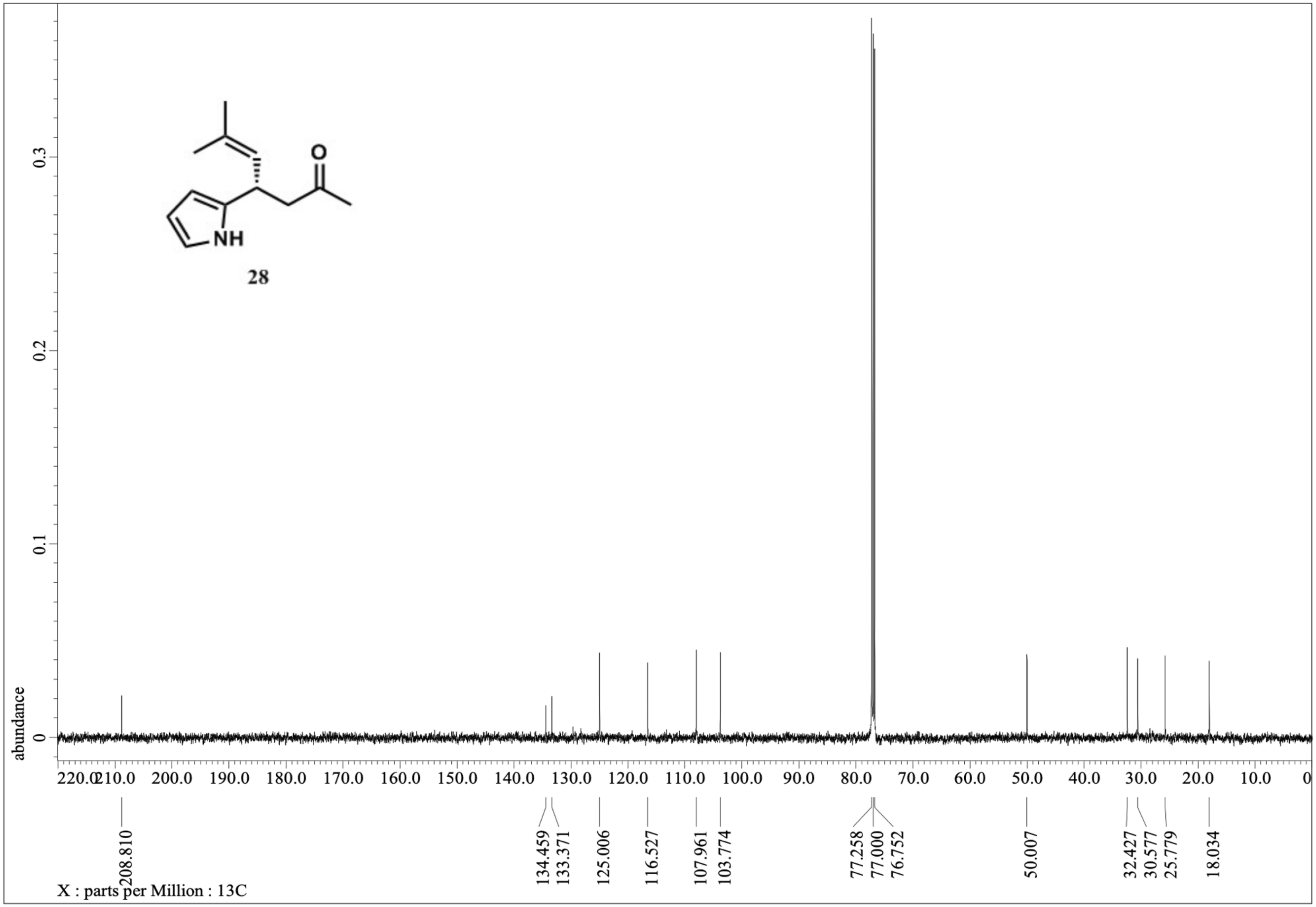

28
0.3
0.2
0.1
0
abundance
220.0
210.0
200.0
190.0
180.0
170.0
160.0
150.0
140.0
130.0
120.0
110.0
100.0
90.0
80.0
70.0
60.0
50.0
40.0
30.0
20.0
10.0
0
208.810
134.459
133.371
125.006
116.527
107.961
103.774
77.258
77.000
76.752
50.007
32.427
30.577
25.779
18.034
X : parts per Million : 13C

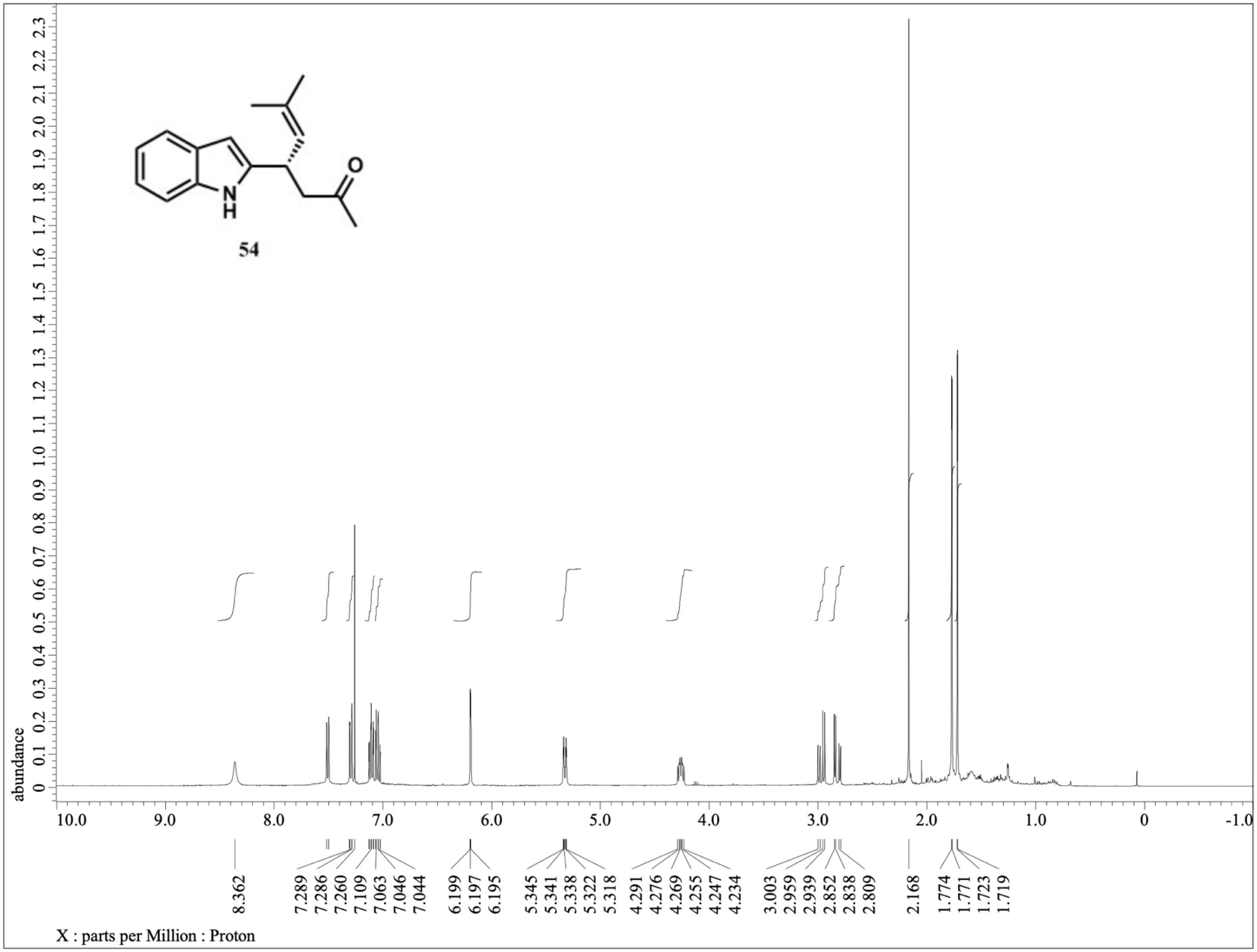

54
abundance
8.362
7.289
7.286
7.260
7.109
7.063
7.046
7.044
6.199
6.197
6.195
5.345
5.341
5.338
5.322
5.318
4.291
4.276
4.269
4.255
4.247
4.234
3.003
2.959
2.939
2.852
2.838
2.809
2.168
1.774
1.771
1.723
1.719
X : parts per Million : Proton

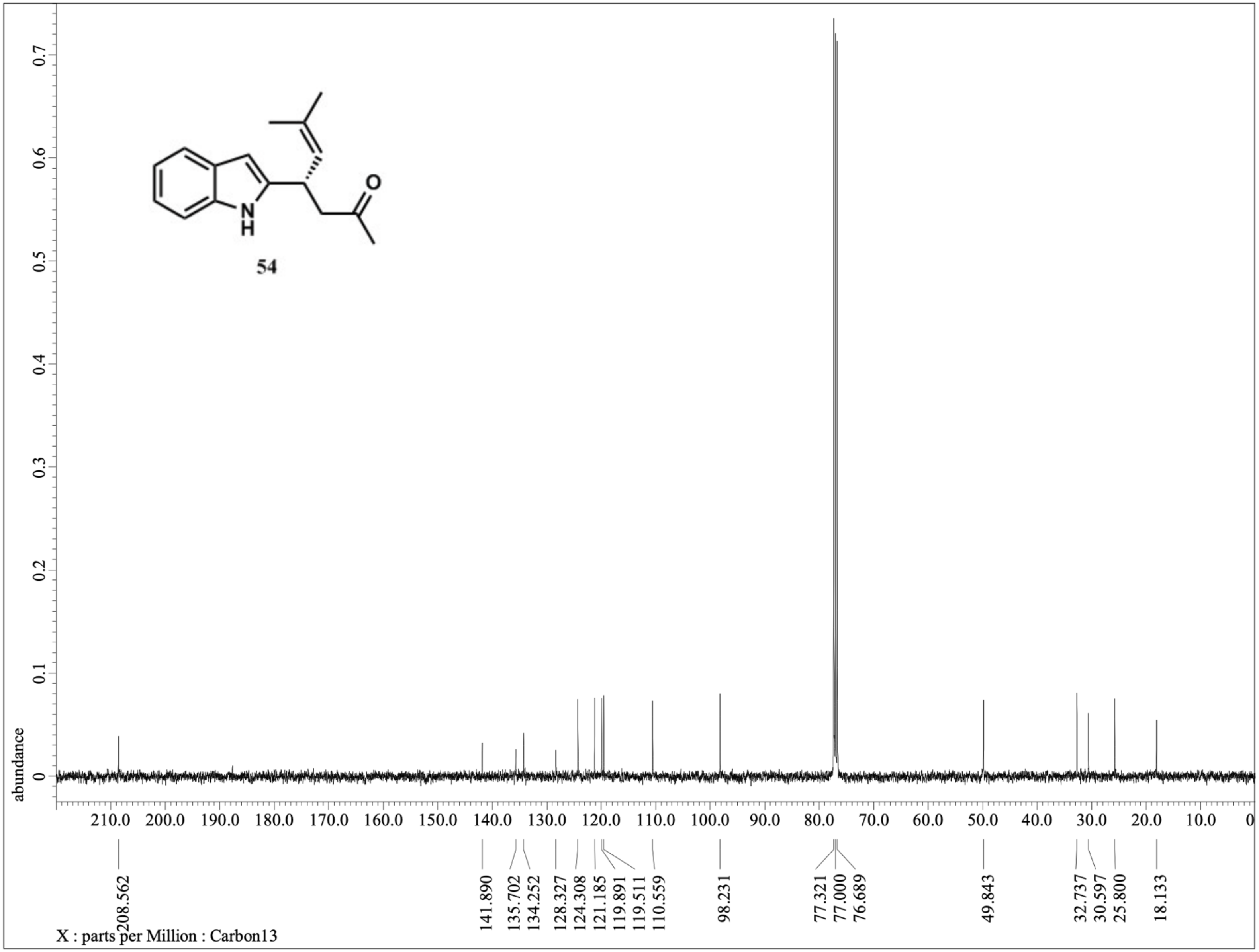

54
abundance
0.7
0.6
0.5
0.4
0.3
0.2
0.1
0
210.0 200.0 190.0 180.0 170.0 160.0 150.0 140.0 130.0 120.0 110.0 100.0 90.0 80.0 70.0 60.0 50.0 40.0 30.0 20.0 10.0 0
208.562
141.890
135.702
134.252
128.327
124.308
121.185
119.891
119.511
110.559
98.231
77.321
77.000
76.689
49.843
32.737
30.597
25.800
18.133
X : parts per Million : Carbon13

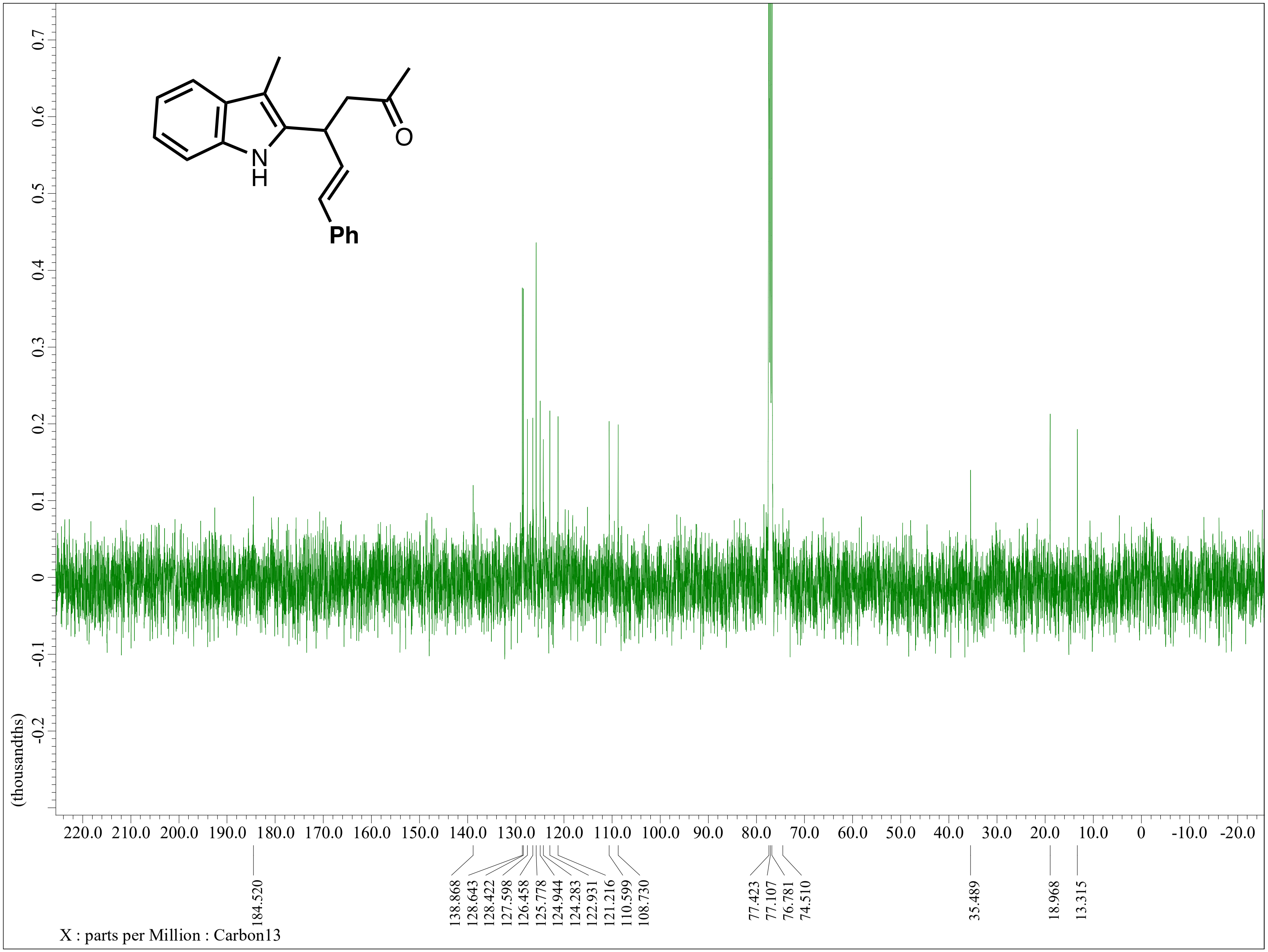

Ph
N
H
O
0.7
0.6
0.5
0.4
0.3
0.2
0.1
0
-0.1
-0.2
(thousandths)
220.0 210.0 200.0 190.0 180.0 170.0 160.0 150.0 140.0 130.0 120.0 110.0 100.0 90.0 80.0 70.0 60.0 50.0 40.0 30.0 20.0 10.0 0 -10.0 -20.0
184.520
138.868
128.643
128.422
127.598
126.458
125.778
124.944
124.283
122.931
121.216
110.599
108.730
77.423
77.107
76.781
74.510
35.489
18.968
13.315
X : parts per Million : Carbon13

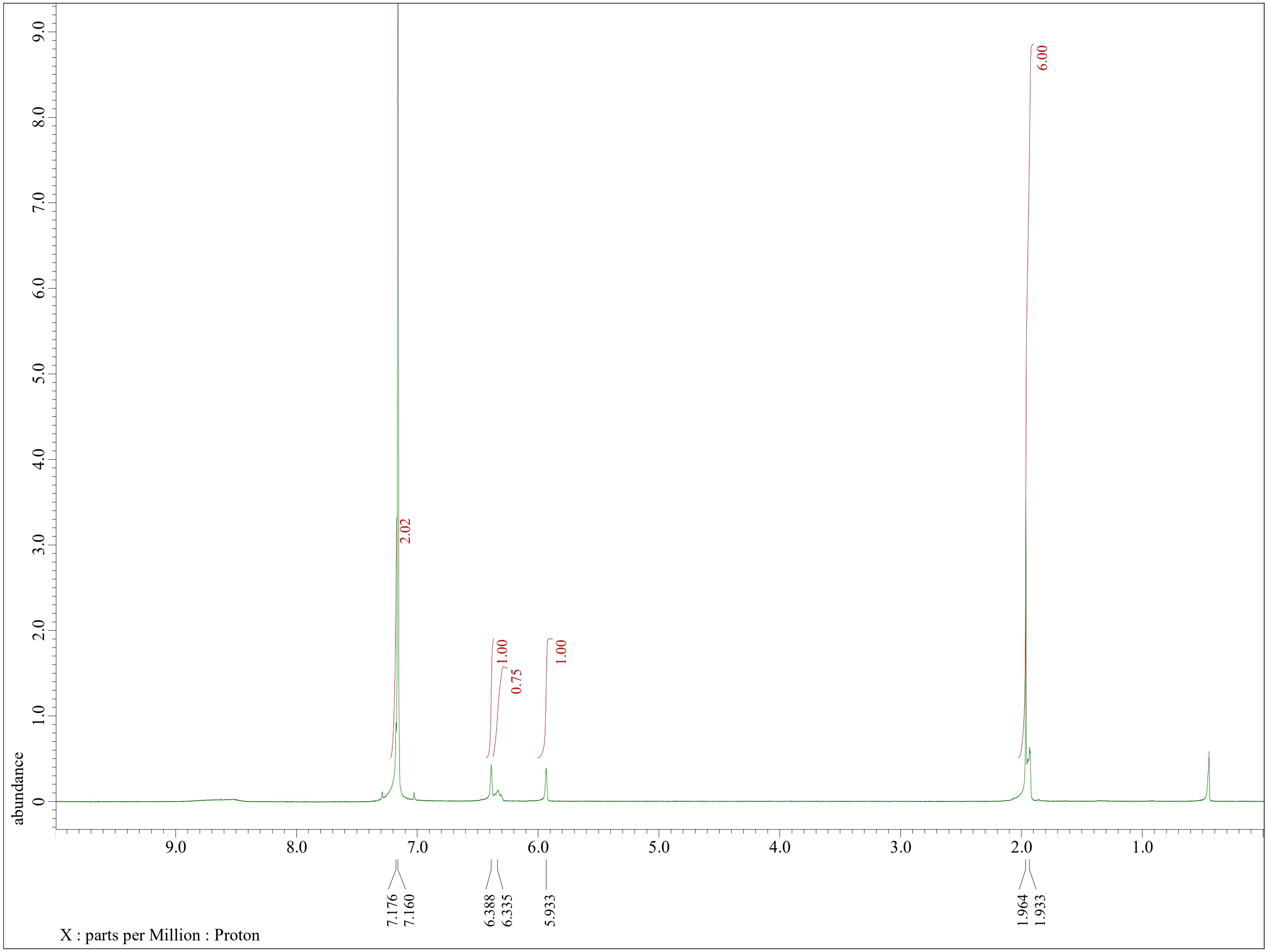

abundance
0
1.0
2.0
3.0
4.0
5.0
6.0
7.0
8.0
9.0
2.02
1.00
0.75
1.00
6.00
7.176
7.160
6.388
6.335
5.933
1.964
1.933
X : parts per Million : Proton

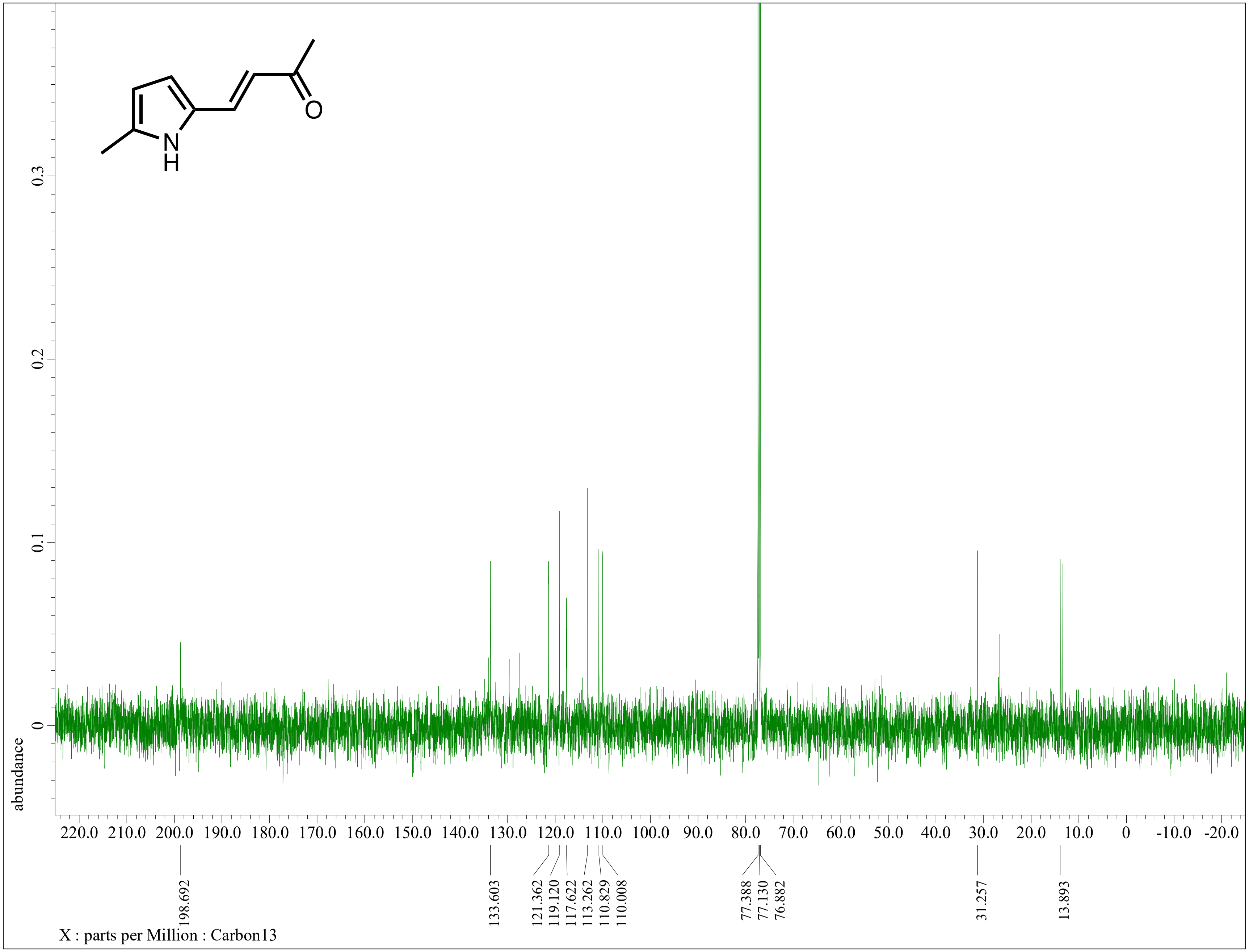

N
H
O
abundance
0.3
0.2
0.1
0
220.0 210.0 200.0 190.0 180.0 170.0 160.0 150.0 140.0 130.0 120.0 110.0 100.0 90.0 80.0 70.0 60.0 50.0 40.0 30.0 20.0 10.0 0 -10.0 -20.0
198.692
133.603
121.362
119.120
117.622
113.262
110.829
110.008
77.388
77.130
76.882
31.257
13.893
X : parts per Million : Carbon13

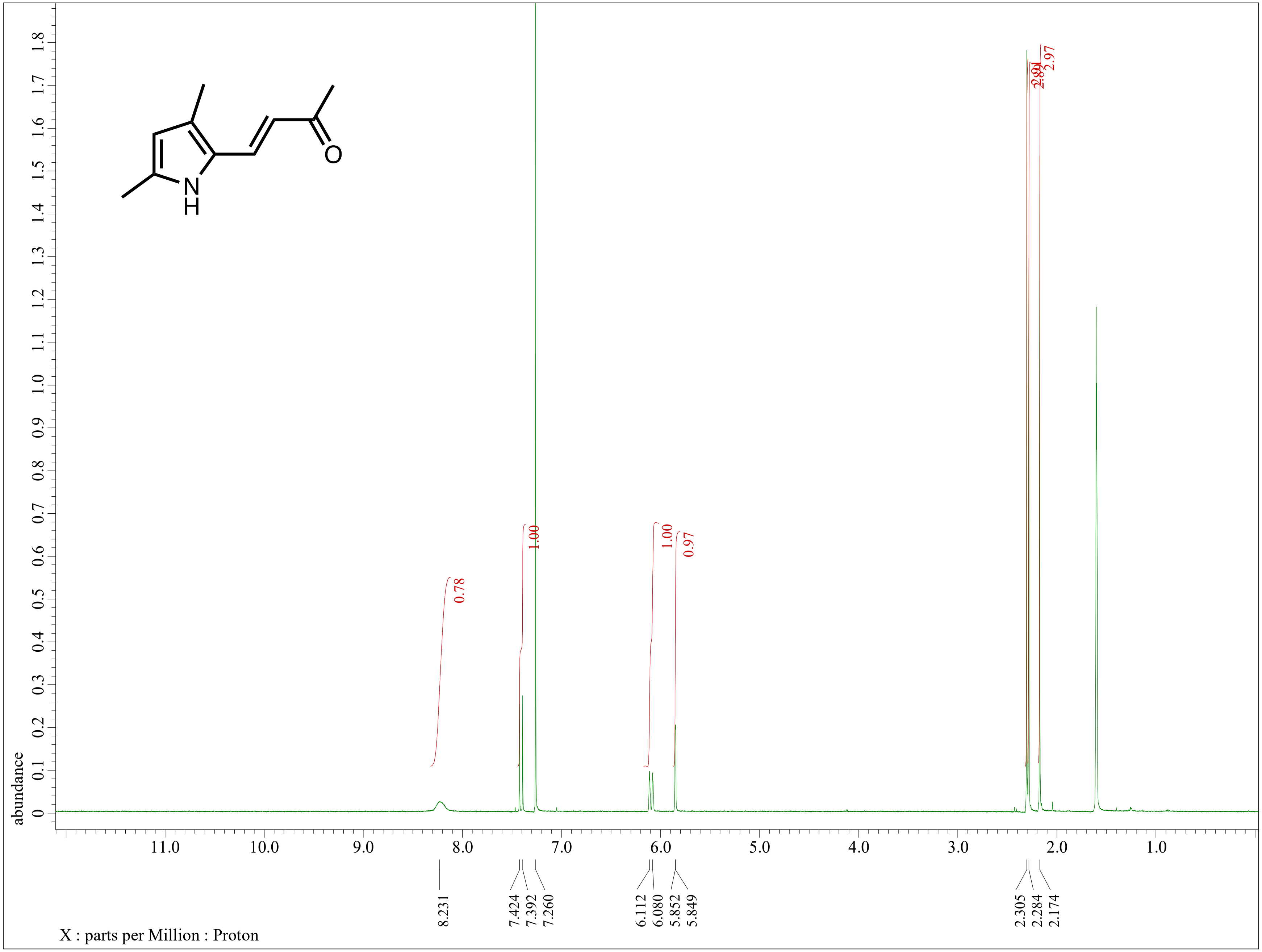
abundance
X : parts per Million : Proton
8.231
7.424
7.392
7.260
6.112
6.080
5.852
5.849
2.305
2.284
2.174
0.78
1.00
1.00
0.97
2.97

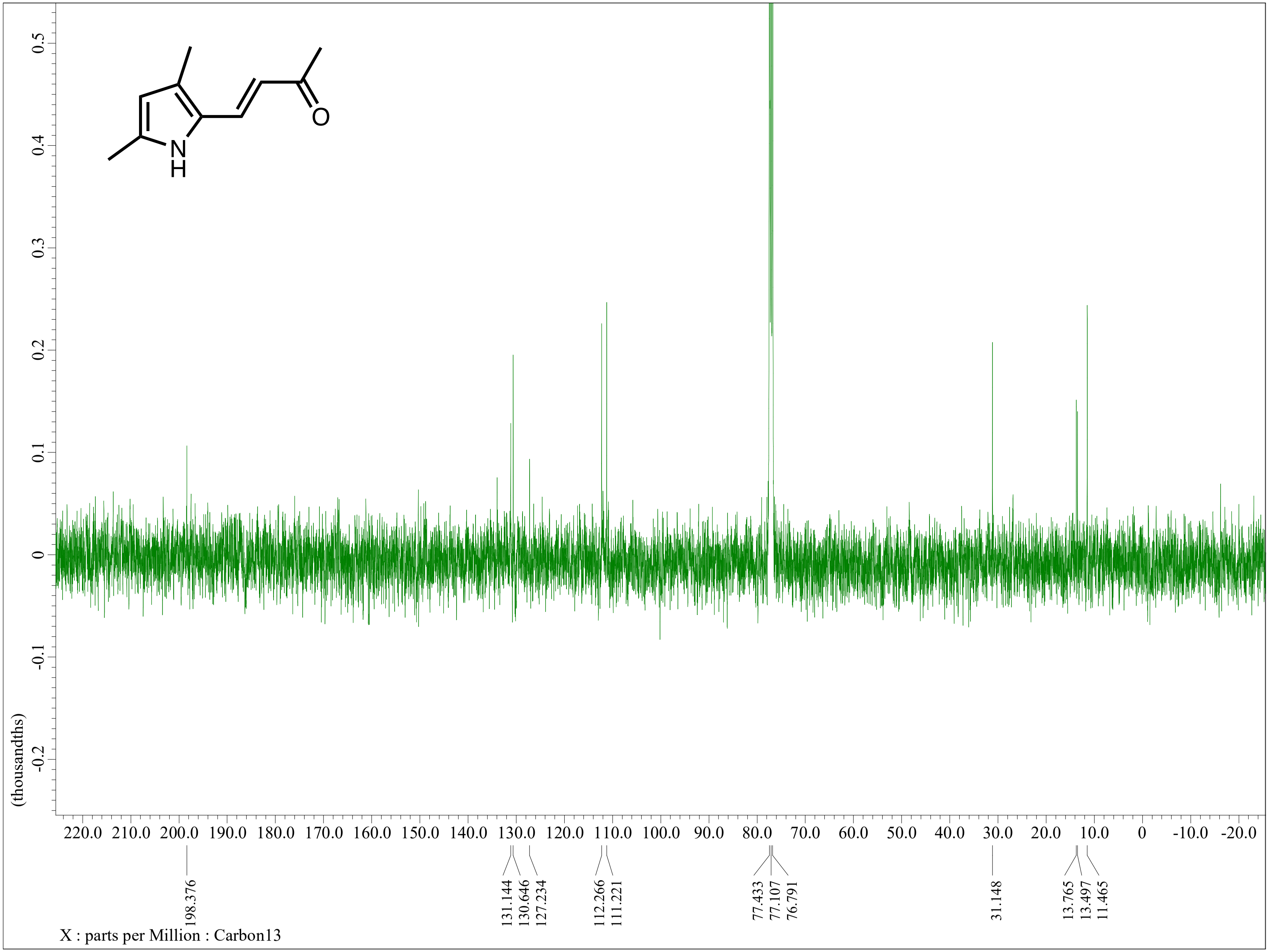
N
H
O
198.376
131.144
130.646
127.234
112.266
111.221
77.433
77.107
76.791
31.148
13.765
13.497
11.465
220.0 210.0 200.0 190.0 180.0 170.0 160.0 150.0 140.0 130.0 120.0 110.0 100.0 90.0 80.0 70.0 60.0 50.0 40.0 30.0 20.0 10.0 0 -10.0 -20.0
0.5 0.4 0.3 0.2 0.1 0 -0.1 -0.2
(thousandths)
X : parts per Million : Carbon13

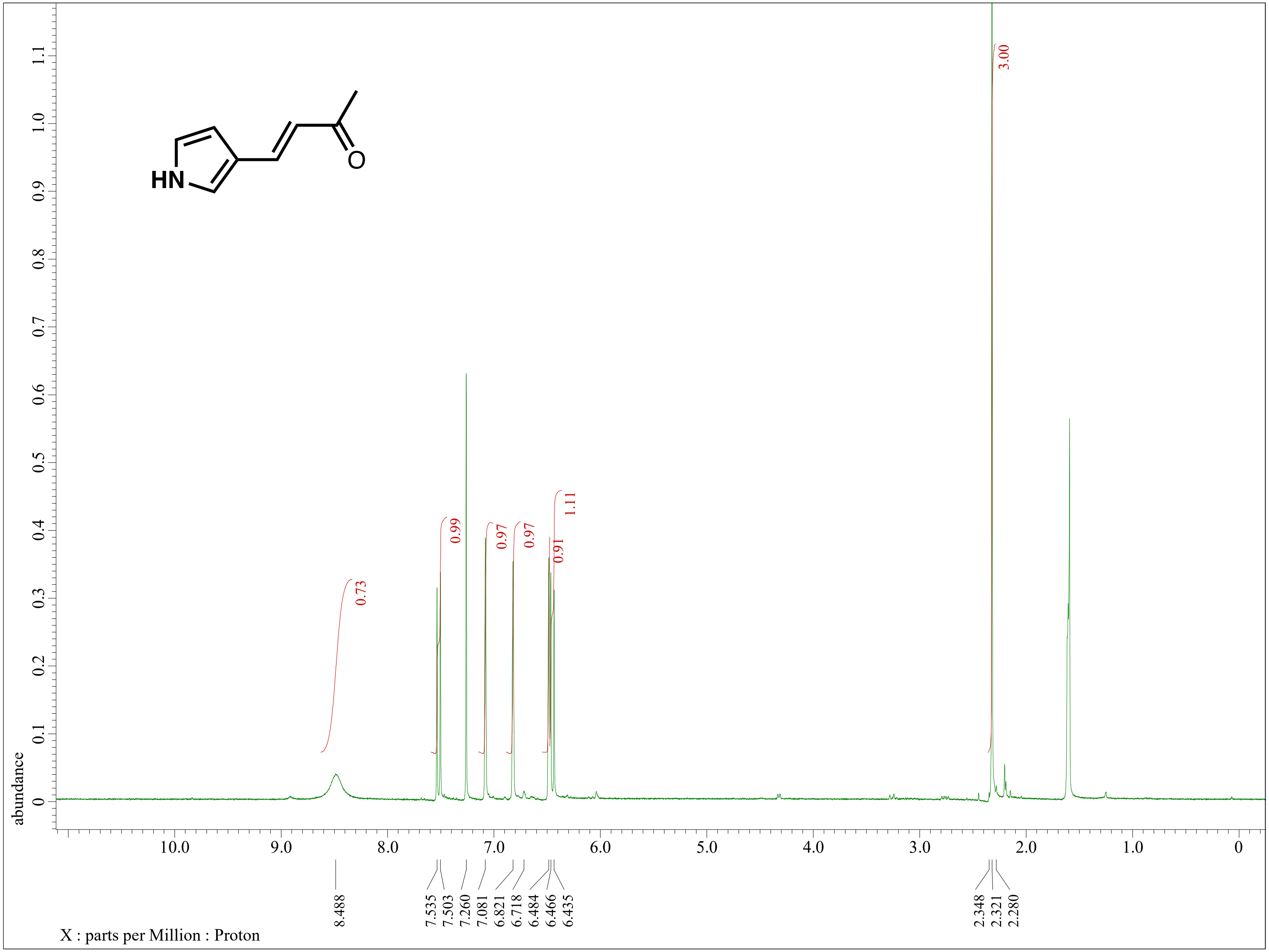

HN
O
abundance
1.1
1.0
0.9
0.8
0.7
0.6
0.5
0.4
0.3
0.2
0.1
0
3.00
0.73
0.99
0.97
0.97
0.91
1.11
10.0
9.0
8.0
7.0
6.0
5.0
4.0
3.0
2.0
1.0
0
8.488
7.535
7.503
7.260
7.081
6.821
6.718
6.484
6.466
6.435
2.348
2.321
2.280
X : parts per Million : Proton

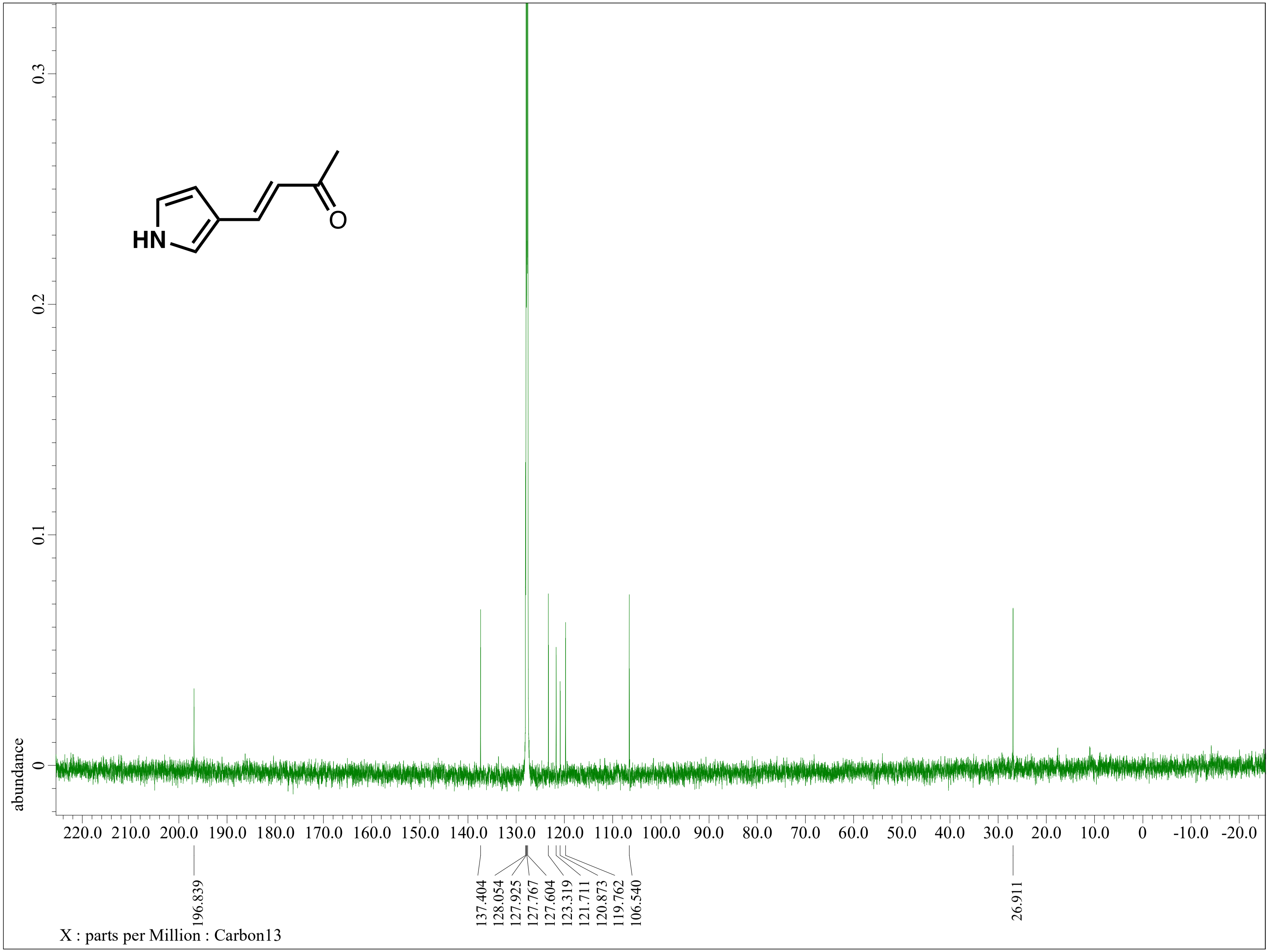

HN
O
0.3
0.2
0.1
0
abundance
220.0 210.0 200.0 190.0 180.0 170.0 160.0 150.0 140.0 130.0 120.0 110.0 100.0 90.0 80.0 70.0 60.0 50.0 40.0 30.0 20.0 10.0 0 -10.0 -20.0
196.839
137.404
128.054
127.925
127.767
127.604
123.319
121.711
120.873
119.762
106.540
26.911
X : parts per Million : Carbon13

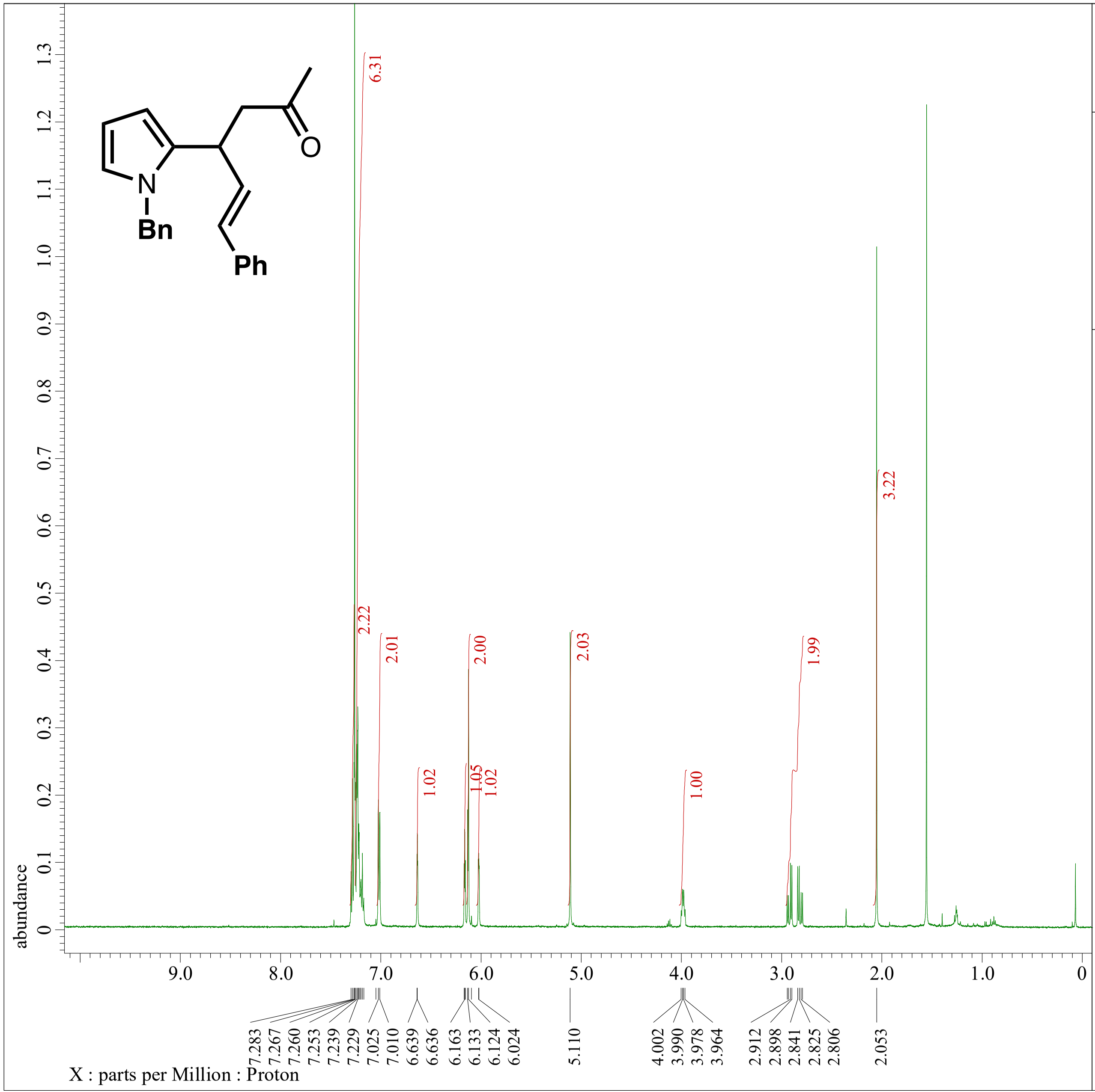


```
---- PROCESSING PARAMETERS ----
dc_balance( 0, FALSE )
sexp( 0.2[Hz], 0.0[s] )
trapezoid( 0[%], 0[%], 80[%], 100[%] )
zerofill( 1 )
fft( 1, TRUE, TRUE )
machinephase
ppm

Derived from: AEB-IV-169_PROTON-2-1.jdf

Filename            = AEB-IV-169_PROTON-2-2.jd
Author              = May
Experiment          = proton.jxp
Sample_Id           = AEB-IV-169
Solvent             = CHLOROFORM-D
Creation_Time       =  8-OCT-2020 13:06:21
Revision_Time       =  8-OCT-2020 13:14:46
Current_Time        =  8-OCT-2020 13:15:16

Data_Format         = 1D COMPLEX
Dim_Size            = 13107
Dim_Title           = Proton
Dim_Units           = [ppm]
Dimensions          = X
Site                = SERC500
Spectrometer        = DELTA2_NMR

Field_Strength      = 11.7441[T] (500[MHz])
X_Acq_Duration      = 1.74587904[s]
X_Domain            = 1H
X_Freq              = 500.02120543[MHz]
X_Offset            = 5[ppm]
X_Points            = 16384
X_Prescans          = 1
X_Resolution        = 0.57277737[Hz]
X_Sweep             = 9.38438438[kHz]
X_Sweep_Clipped     = 7.50750751[kHz]
Irr_Domain          = Proton
Irr_Freq            = 500.02120543[MHz]
Irr_Offset          = 5[ppm]
Tri_Domain          = Proton
Tri_Freq            = 500.02120543[MHz]
Tri_Offset          = 5[ppm]
Clipped             = FALSE
Scans               = 16
Total_Scans         = 16

Relaxation_Delay    = 4[s]
Recvr_Gain          = 46
Temp_Get            = 18.8[dC]
X_90_Width          = 12.4[us]
X_Acq_Time          = 1.74587904[s]
X_Angle             = 45[deg]
X_Atn               = 3[dB]
X_Pulse             = 6.2[us]
Irr_Mode            = Off
Tri_Mode            = Off
Dante_Presat        = FALSE
Initial_Wait        = 1[s]
```

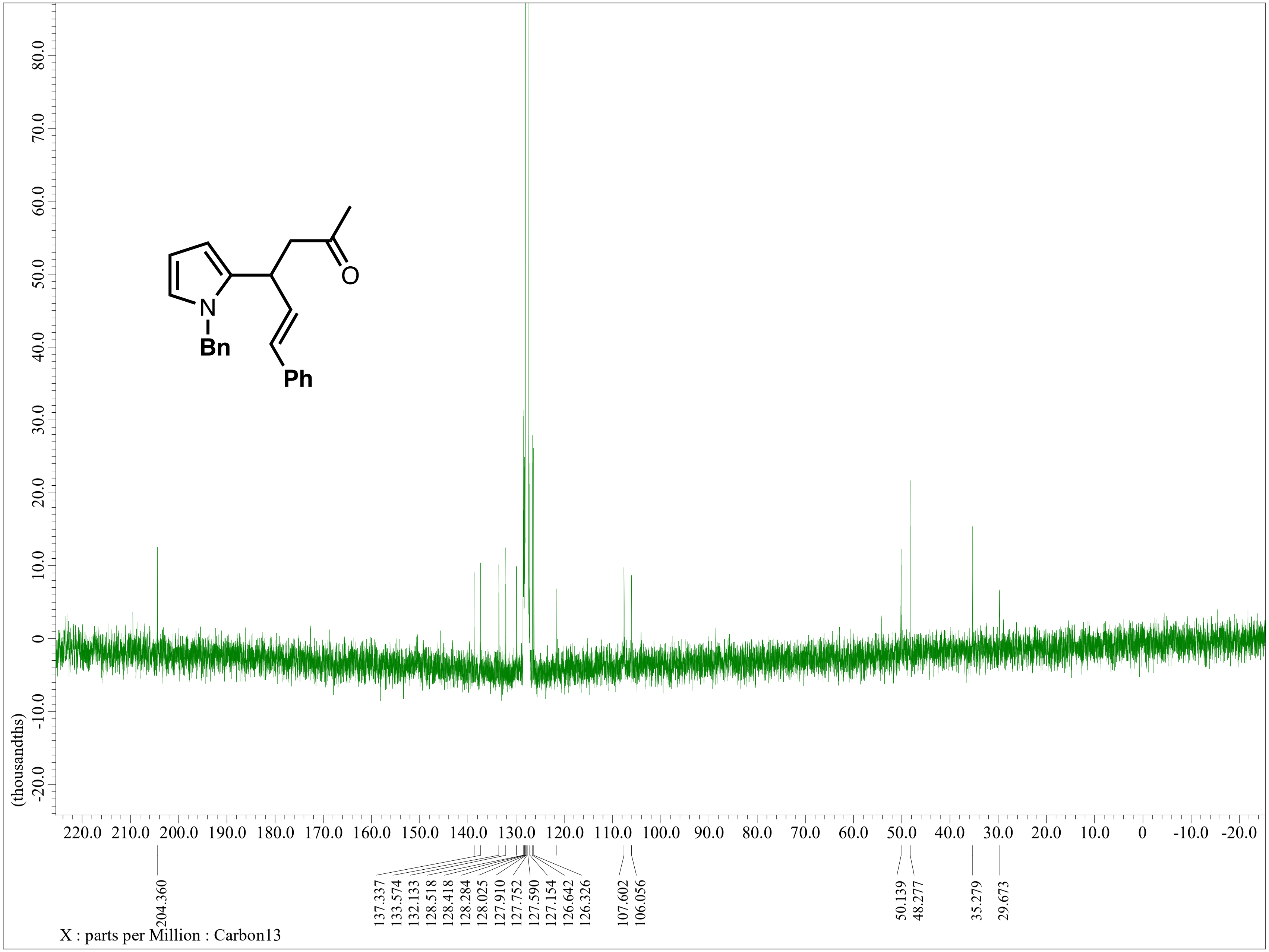

N
Bn
Ph
O
(thousandths)
80.0
70.0
60.0
50.0
40.0
30.0
20.0
10.0
0
-10.0
-20.0
220.0 210.0 200.0 190.0 180.0 170.0 160.0 150.0 140.0 130.0 120.0 110.0 100.0 90.0 80.0 70.0 60.0 50.0 40.0 30.0 20.0 10.0 0 -10.0 -20.0
204.360
137.337
133.574
132.133
128.518
128.418
128.284
128.025
127.910
127.752
127.590
127.154
126.642
126.326
107.602
106.056
50.139
48.277
35.279
29.673
X : parts per Million : Carbon13

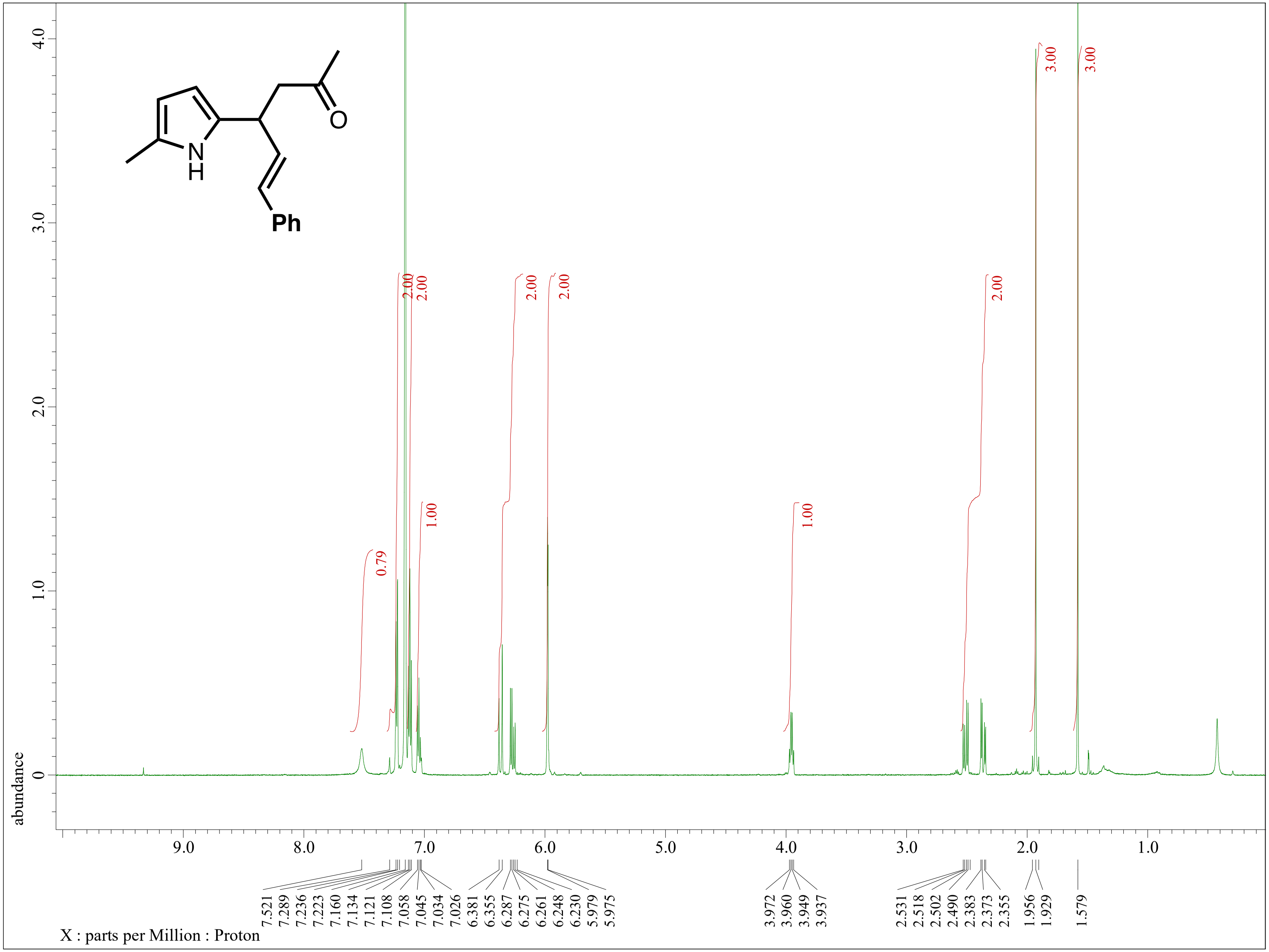

NH
Ph
O
abundance
4.0
3.0
2.0
1.0
0
0.79
2.00
2.00
1.00
2.00
2.00
1.00
2.00
3.00
3.00
9.0
8.0
7.0
6.0
5.0
4.0
3.0
2.0
1.0
7.521
7.289
7.236
7.223
7.160
7.134
7.121
7.108
7.058
7.045
7.034
7.026
6.381
6.355
6.287
6.275
6.261
6.248
6.230
5.979
5.975
3.972
3.960
3.949
3.937
2.531
2.518
2.502
2.490
2.383
2.373
2.355
1.956
1.929
1.579
X : parts per Million : Proton

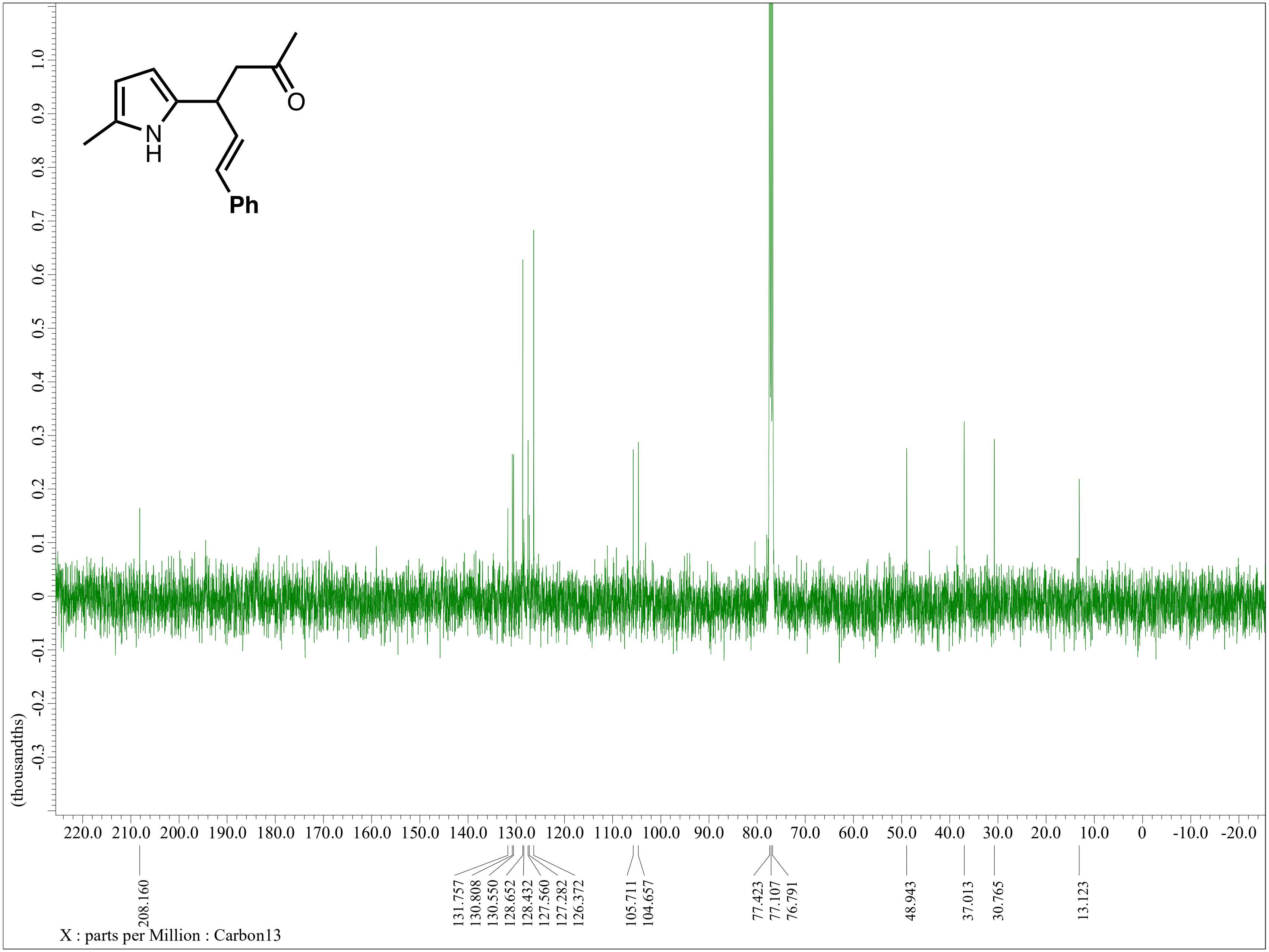

Ph
N
H
O
(thousandths)
1.0
0.9
0.8
0.7
0.6
0.5
0.4
0.3
0.2
0.1
0
-0.1
-0.2
-0.3
220.0 210.0 200.0 190.0 180.0 170.0 160.0 150.0 140.0 130.0 120.0 110.0 100.0 90.0 80.0 70.0 60.0 50.0 40.0 30.0 20.0 10.0 0 -10.0 -20.0
208.160
131.757
130.808
130.550
128.652
128.432
127.560
127.282
126.372
105.711
104.657
77.423
77.107
76.791
48.943
37.013
30.765
13.123
X : parts per Million : Carbon13

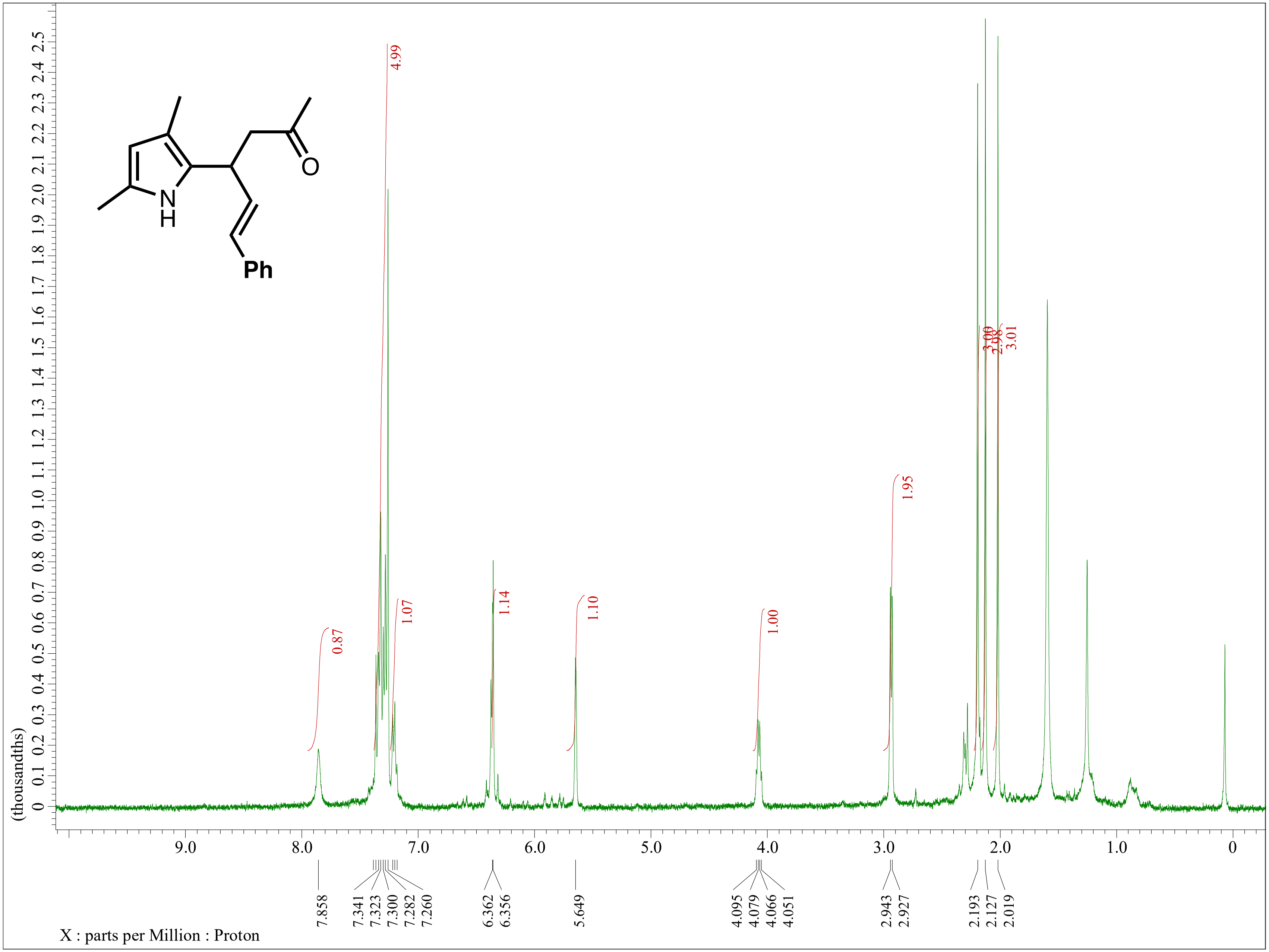

Ph
N
H
O
4.99
0.87
1.07
1.14
1.10
1.00
1.95
3.00
2.98
3.01
7.858
7.341
7.323
7.300
7.282
7.260
6.362
6.356
5.649
4.095
4.079
4.066
4.051
2.943
2.927
2.193
2.127
2.019
(thousandths)
X : parts per Million : Proton

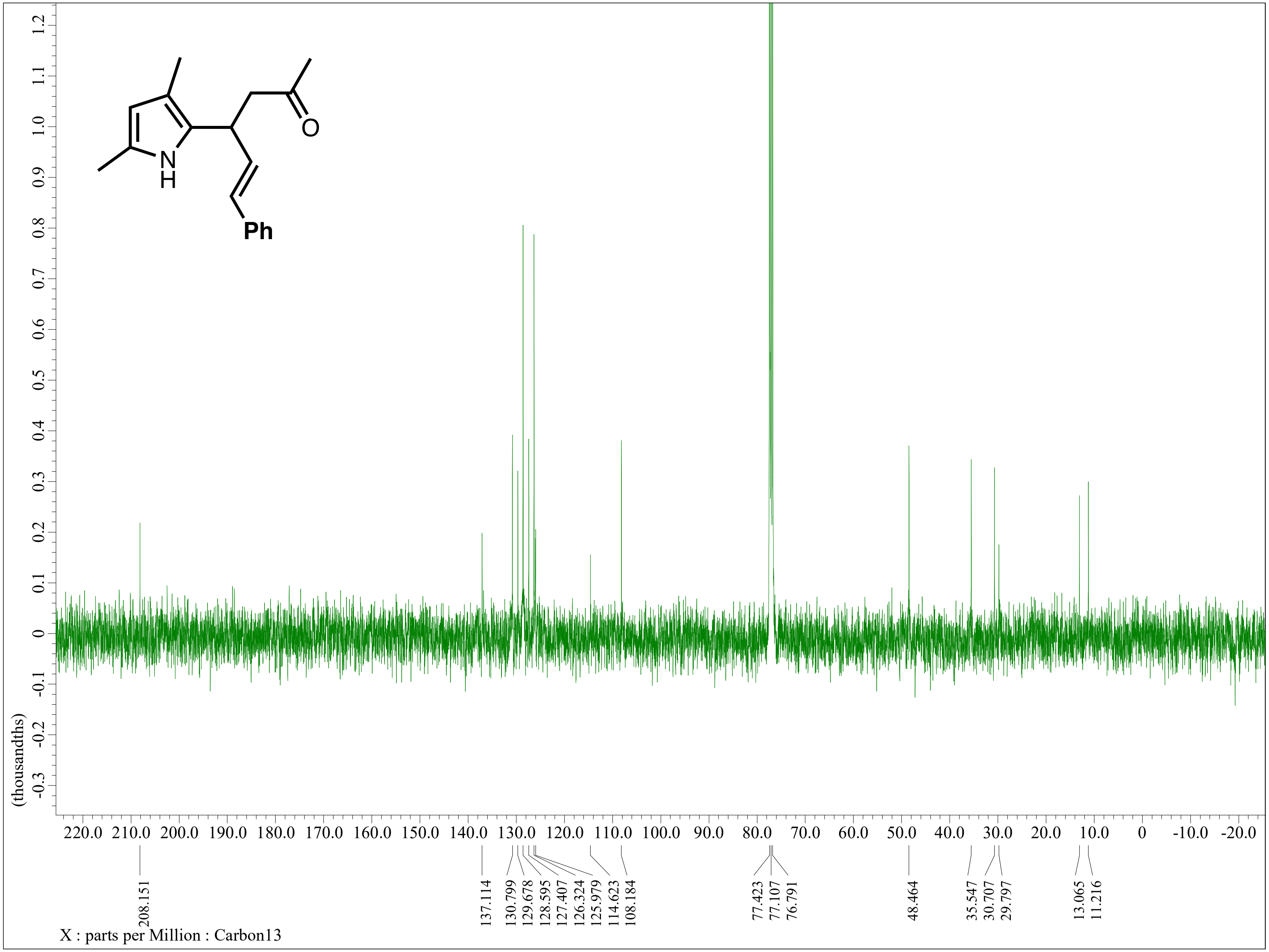

N
H
O
Ph
(thousandths)
1.2
1.1
1.0
0.9
0.8
0.7
0.6
0.5
0.4
0.3
0.2
0.1
0
-0.1
-0.2
-0.3
220.0 210.0 200.0 190.0 180.0 170.0 160.0 150.0 140.0 130.0 120.0 110.0 100.0 90.0 80.0 70.0 60.0 50.0 40.0 30.0 20.0 10.0 0 -10.0 -20.0
208.151
137.114
130.799
129.678
128.595
127.407
126.324
125.979
114.623
108.184
77.423
77.107
76.791
48.464
35.547
30.707
29.797
13.065
11.216
X : parts per Million : Carbon13

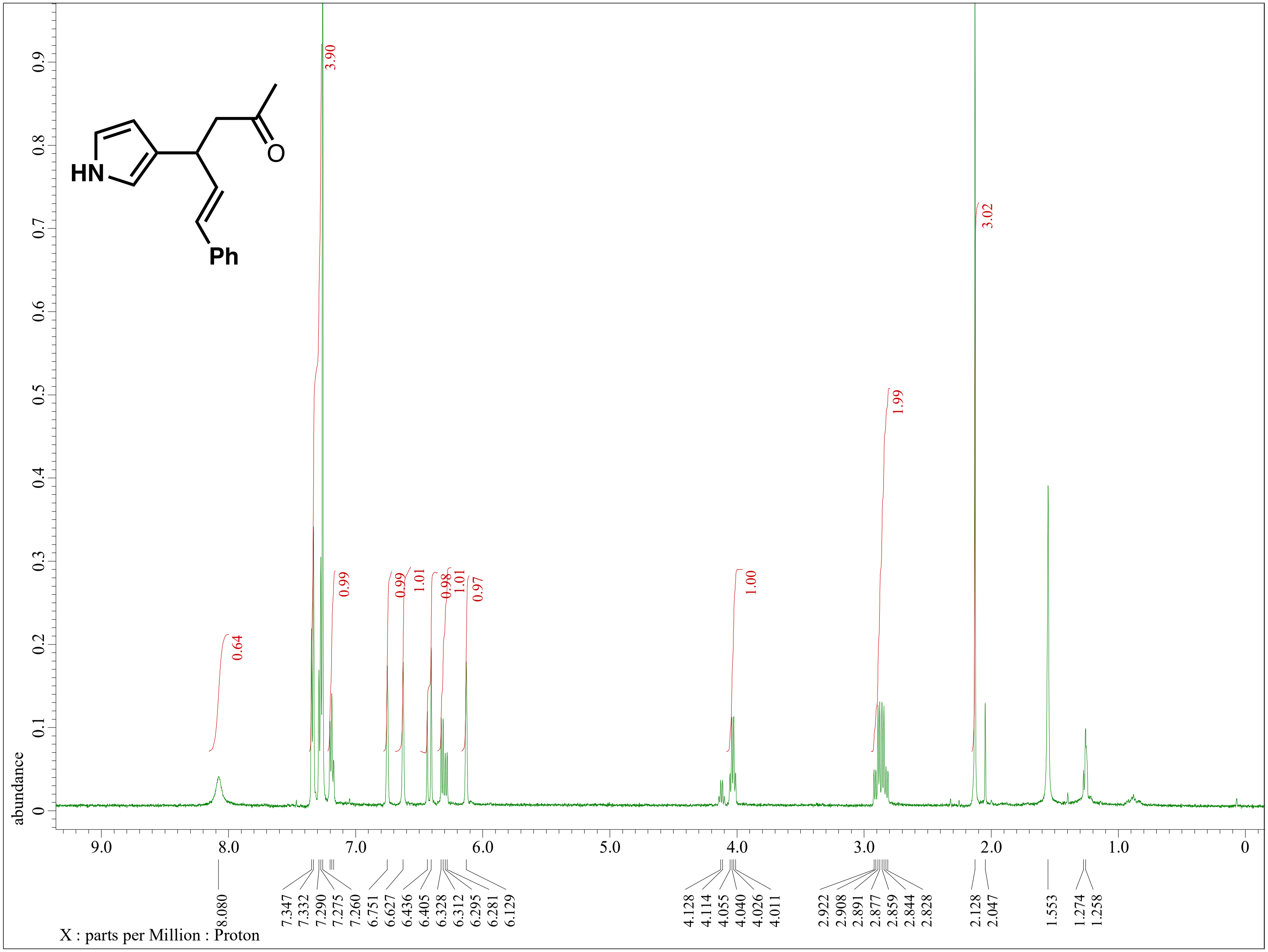
HN
O
Ph
abundance
0.9
0.8
0.7
0.6
0.5
0.4
0.3
0.2
0.1
0
3.90
3.02
1.99
1.00
0.99
0.99
1.01
0.98
1.01
0.97
0.64
9.0
8.0
7.0
6.0
5.0
4.0
3.0
2.0
1.0
0
8.080
7.347
7.332
7.290
7.275
7.260
6.751
6.627
6.436
6.405
6.328
6.312
6.295
6.281
6.129
4.128
4.114
4.055
4.040
4.026
4.011
2.922
2.908
2.891
2.877
2.859
2.844
2.828
2.128
2.047
1.553
1.274
1.258
X : parts per Million : Proton

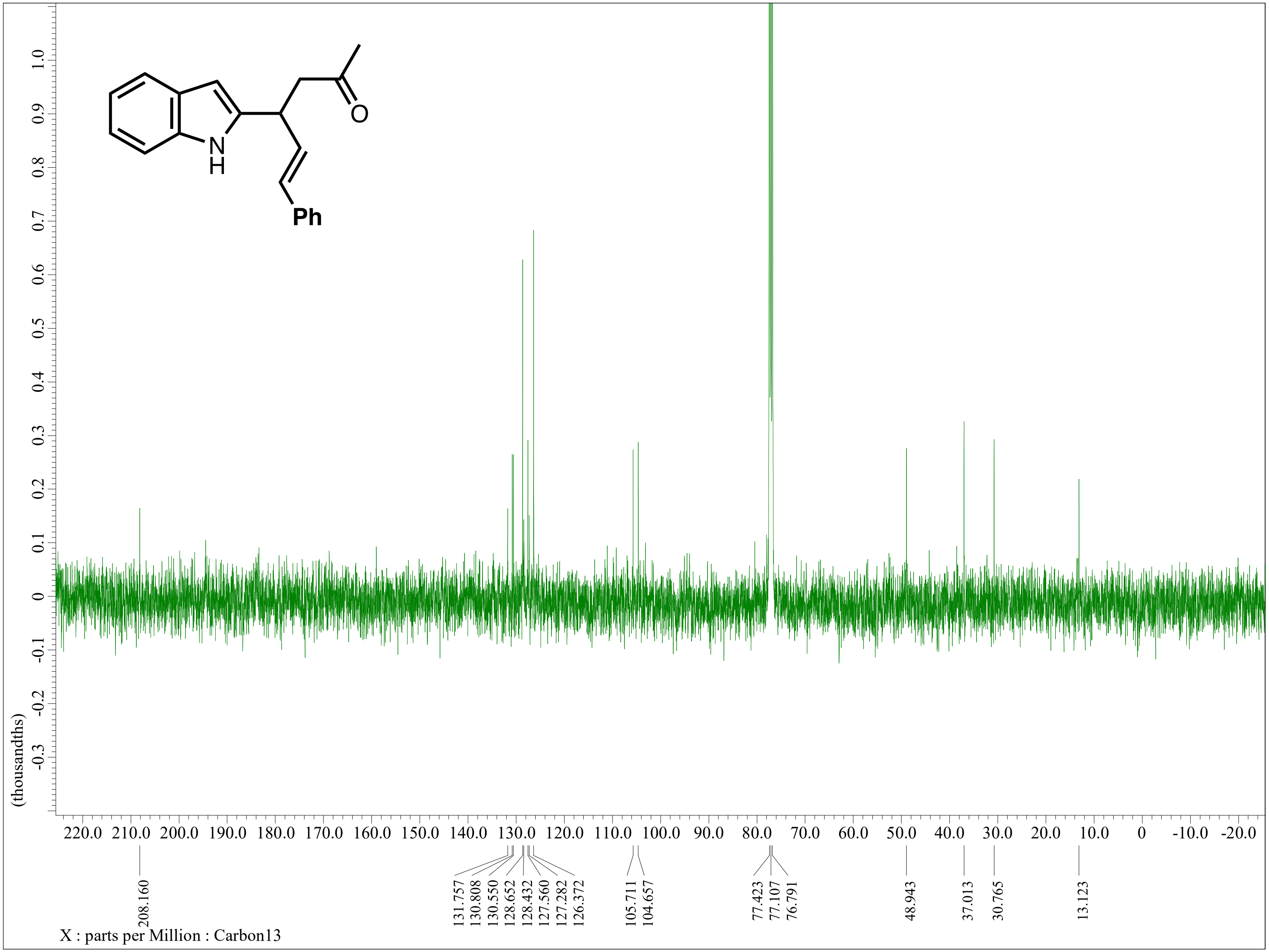

Ph
N
H
O
1.0
0.9
0.8
0.7
0.6
0.5
0.4
0.3
0.2
0.1
0
-0.1
-0.2
-0.3
(thousandths)
220.0 210.0 200.0 190.0 180.0 170.0 160.0 150.0 140.0 130.0 120.0 110.0 100.0 90.0 80.0 70.0 60.0 50.0 40.0 30.0 20.0 10.0 0 -10.0 -20.0
208.160
131.757
130.808
130.550
128.652
128.432
127.560
127.282
126.372
105.711
104.657
77.423
77.107
76.791
48.943
37.013
30.765
13.123
X : parts per Million : Carbon13

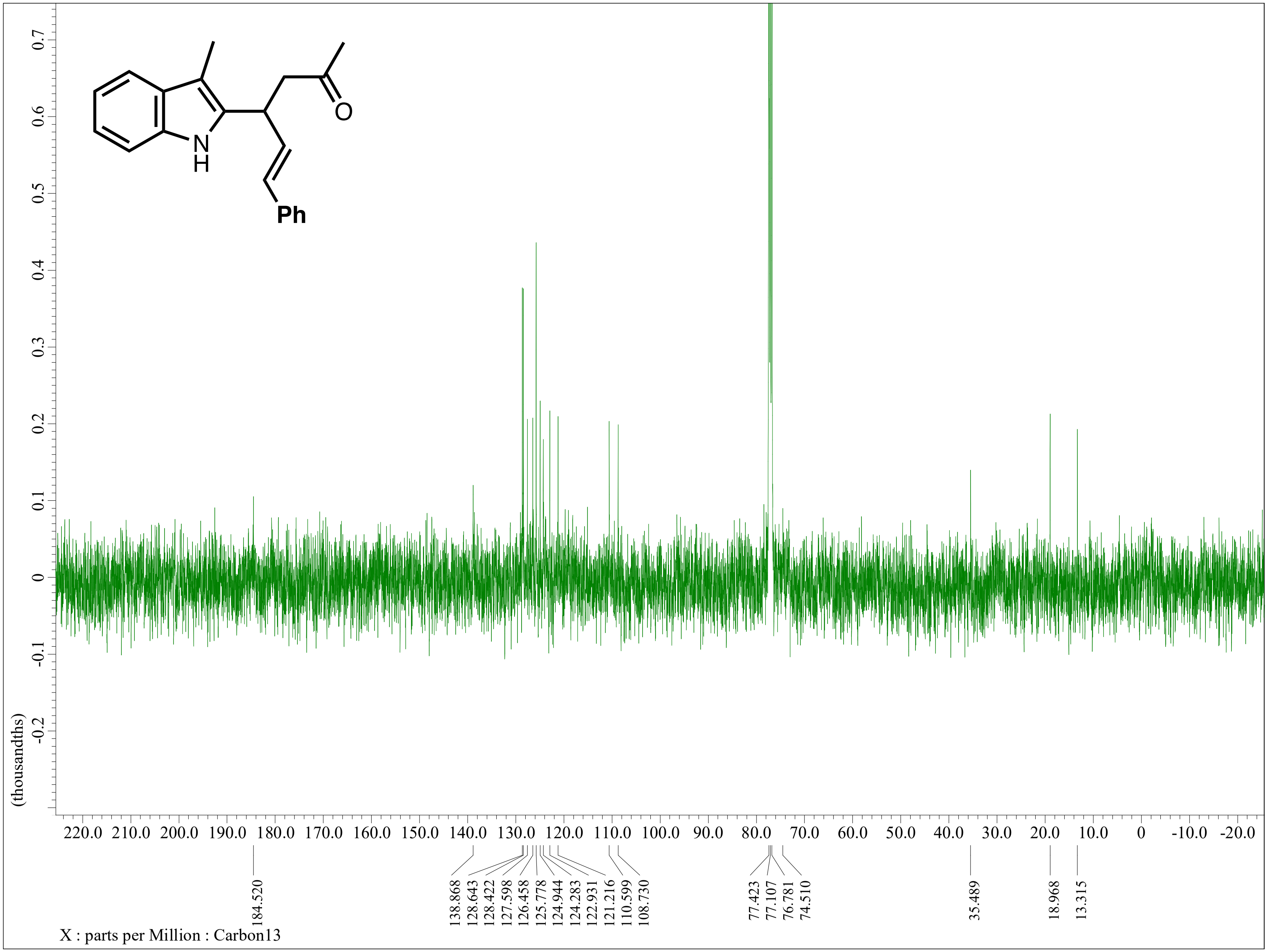

Ph
N
H
O
(thousandths)
0.7
0.6
0.5
0.4
0.3
0.2
0.1
0
-0.1
-0.2
220.0 210.0 200.0 190.0 180.0 170.0 160.0 150.0 140.0 130.0 120.0 110.0 100.0 90.0 80.0 70.0 60.0 50.0 40.0 30.0 20.0 10.0 0 -10.0 -20.0
184.520
138.868
128.643
128.422
127.598
126.458
125.778
124.944
124.283
122.931
121.216
110.599
108.730
77.423
77.107
76.781
74.510
35.489
18.968
13.315
X : parts per Million : Carbon13

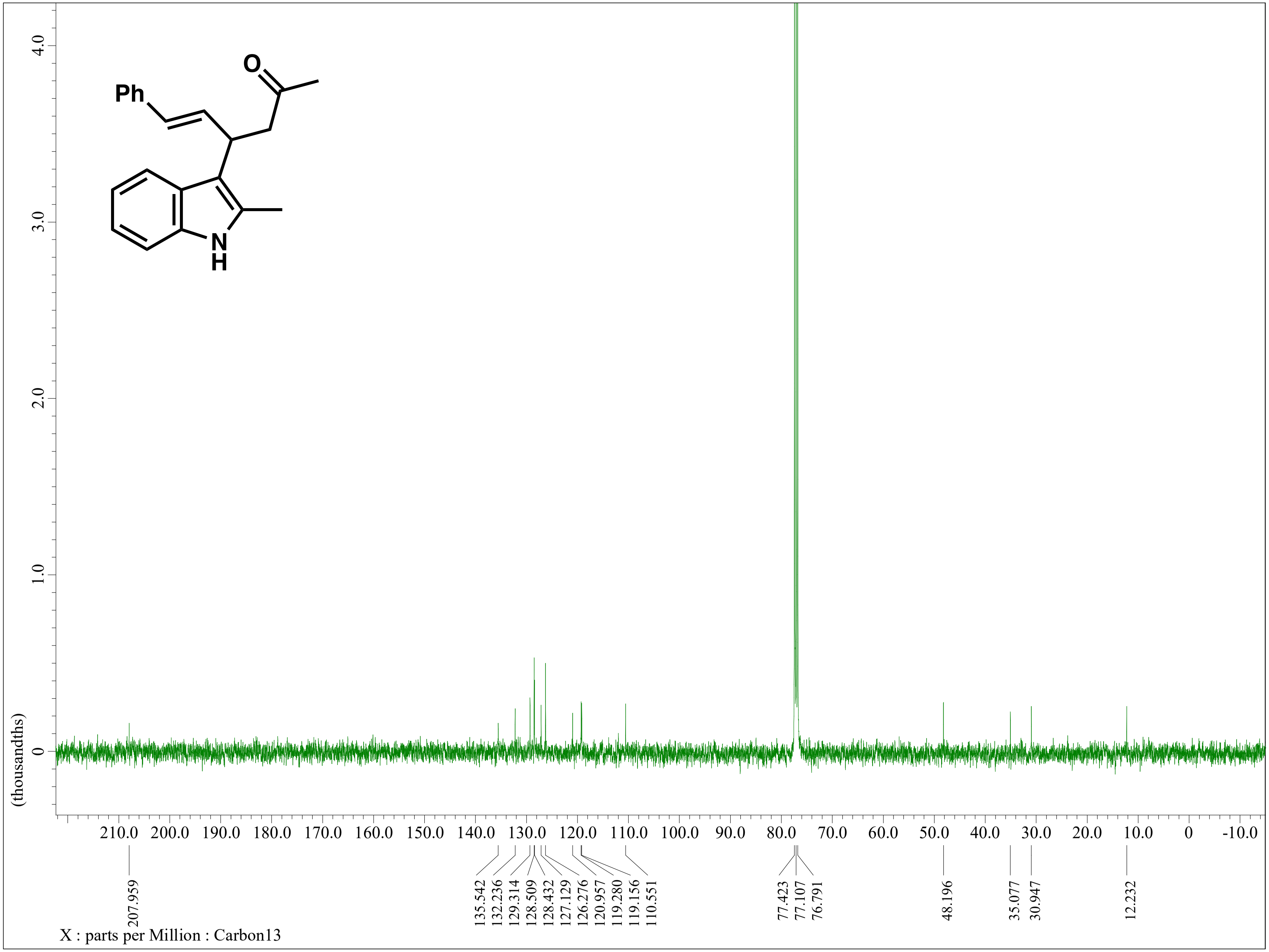
Ph
O
N
H
4.0
3.0
2.0
1.0
0
(thousandths)
210.0 200.0 190.0 180.0 170.0 160.0 150.0 140.0 130.0 120.0 110.0 100.0 90.0 80.0 70.0 60.0 50.0 40.0 30.0 20.0 10.0 0 -10.0
207.959
135.542
132.236
129.314
128.509
128.432
127.129
126.276
120.957
119.280
119.156
110.551
77.423
77.107
76.791
48.196
35.077
30.947
12.232
X : parts per Million : Carbon13

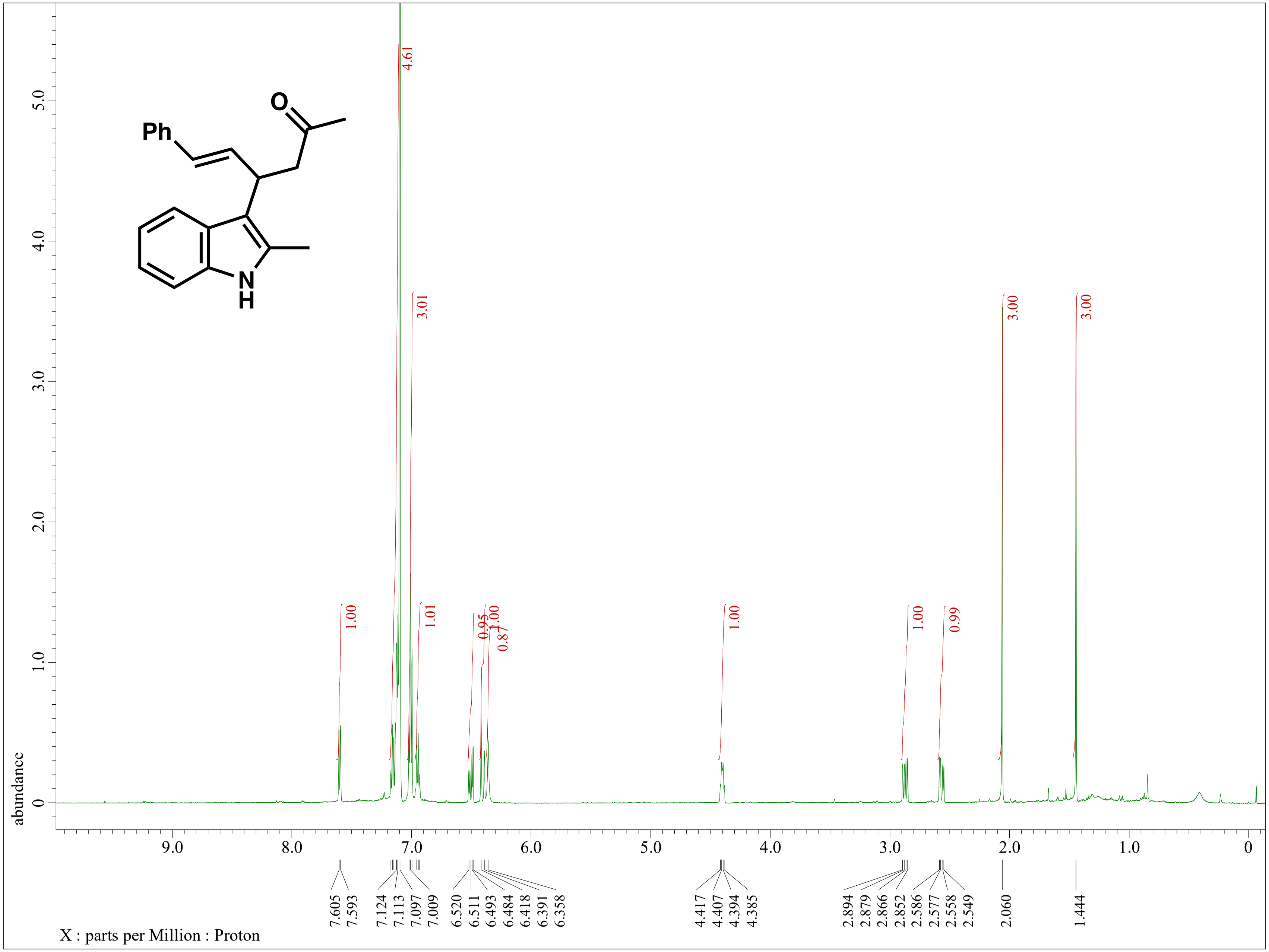
Ph
O
N
H
abundance
5.0
4.0
3.0
2.0
1.0
0
4.61
3.01
1.00
1.01
0.95
1.00
0.87
1.00
1.00
0.99
3.00
3.00
9.0
8.0
7.0
6.0
5.0
4.0
3.0
2.0
1.0
0
7.605
7.593
7.124
7.113
7.097
7.009
6.520
6.511
6.493
6.484
6.418
6.391
6.358
4.417
4.407
4.394
4.385
2.894
2.879
2.866
2.852
2.586
2.577
2.558
2.549
2.060
1.444
X : parts per Million : Proton

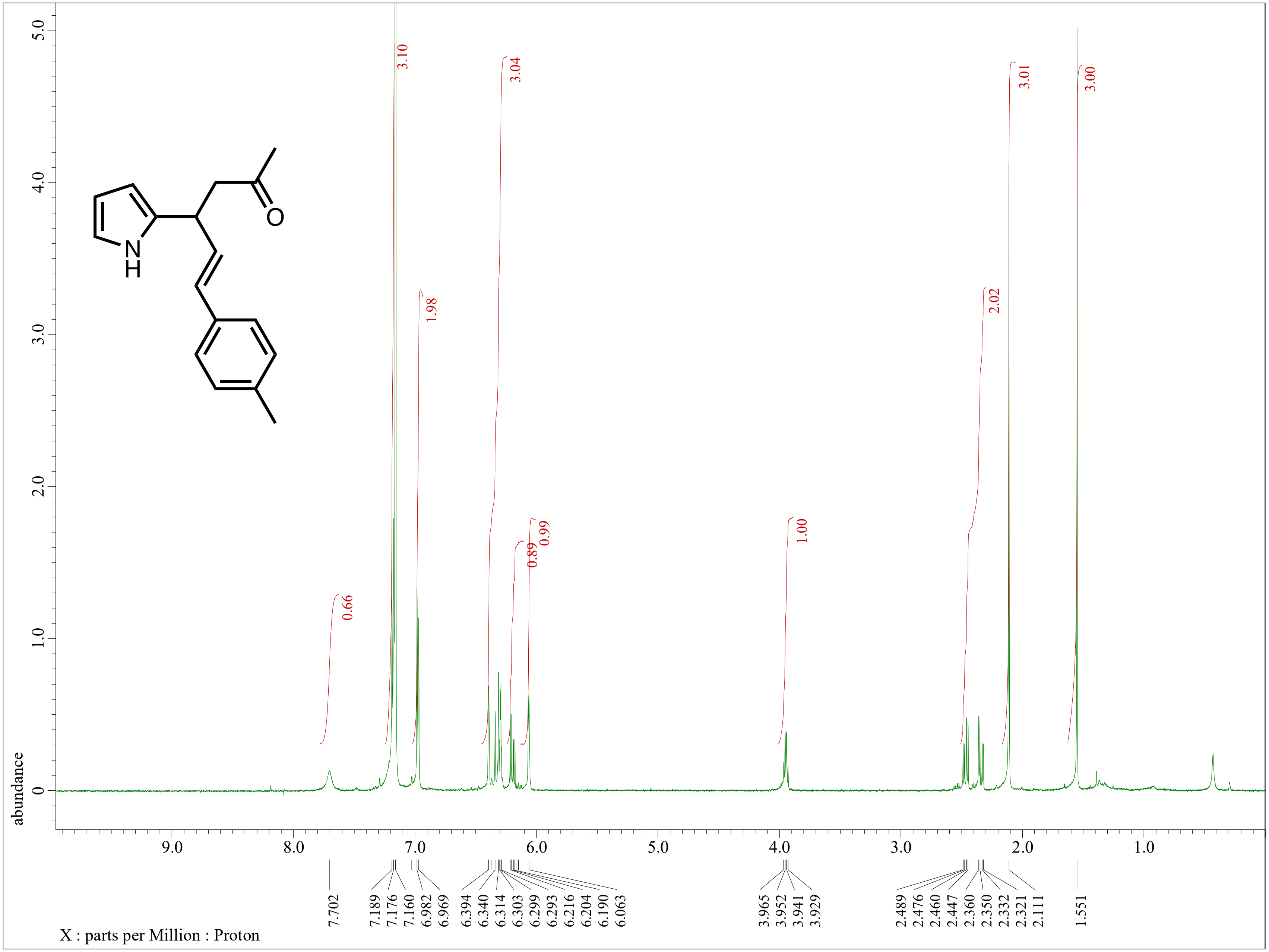

N
H
O
3.10
3.04
1.98
0.66
0.89
0.99
1.00
2.02
3.01
3.00
abundance
5.0
4.0
3.0
2.0
1.0
0
9.0
8.0
7.0
6.0
5.0
4.0
3.0
2.0
1.0
7.702
7.189
7.176
7.160
6.982
6.969
6.394
6.340
6.314
6.303
6.299
6.293
6.216
6.204
6.190
6.063
3.965
3.952
3.941
3.929
2.489
2.476
2.460
2.447
2.360
2.350
2.332
2.321
2.111
1.551
X : parts per Million : Proton

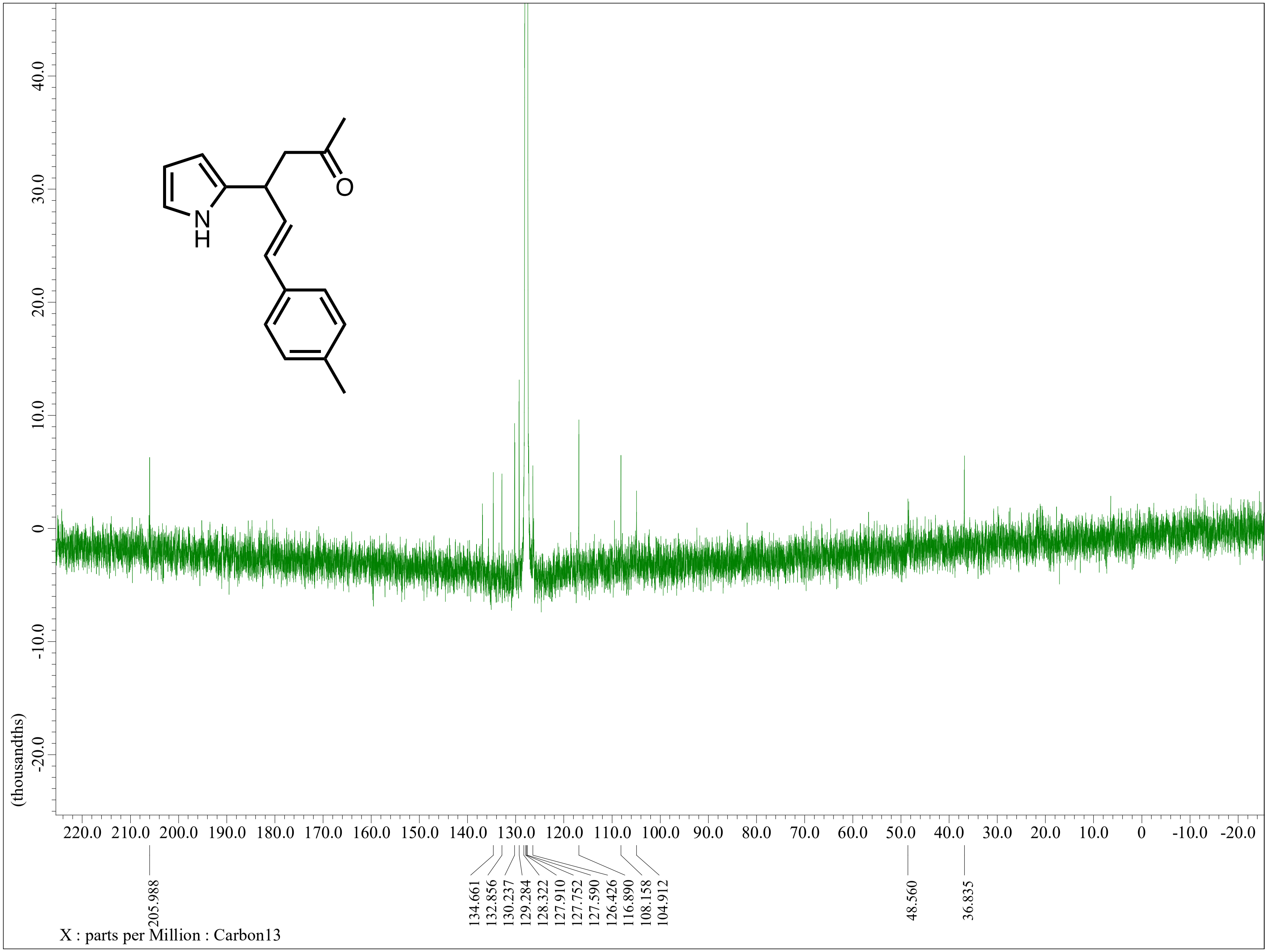

N
H
O
40.0
30.0
20.0
10.0
0
-10.0
-20.0
(thousandths)
220.0 210.0 200.0 190.0 180.0 170.0 160.0 150.0 140.0 130.0 120.0 110.0 100.0 90.0 80.0 70.0 60.0 50.0 40.0 30.0 20.0 10.0 0 -10.0 -20.0
205.988
134.661
132.856
130.237
129.284
128.322
127.910
127.752
127.590
126.426
116.890
108.158
104.912
48.560
36.835
X : parts per Million : Carbon13

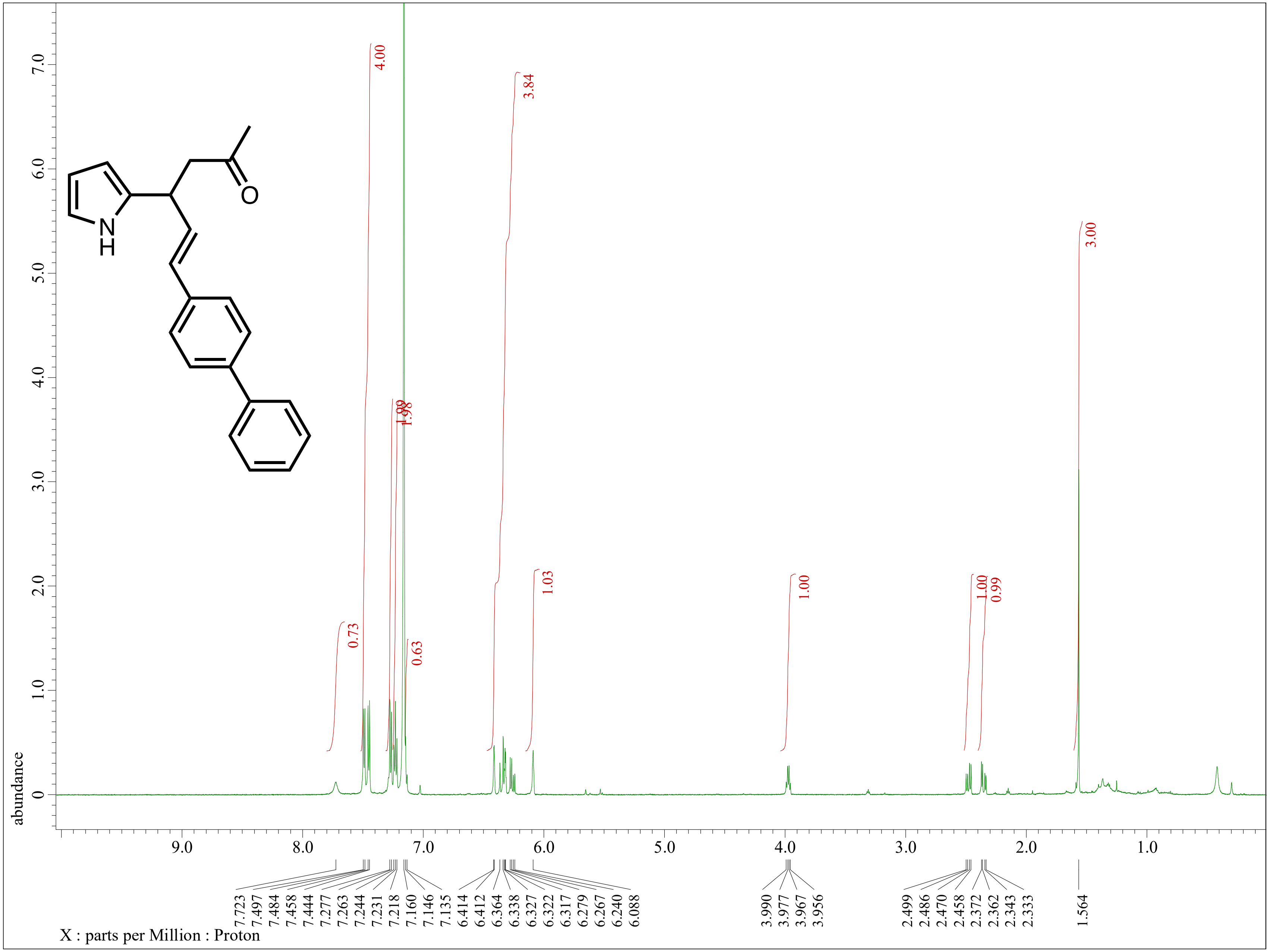

NH
O
abundance
7.0
6.0
5.0
4.0
3.0
2.0
1.0
0
4.00
3.84
3.00
1.98
1.98
1.03
1.00
1.00
0.99
0.73
0.63
9.0
8.0
7.0
6.0
5.0
4.0
3.0
2.0
1.0
7.723
7.497
7.484
7.458
7.444
7.277
7.263
7.244
7.231
7.218
7.160
7.146
7.135
6.414
6.412
6.364
6.338
6.327
6.322
6.317
6.279
6.267
6.240
6.088
3.990
3.977
3.967
3.956
2.499
2.486
2.470
2.458
2.372
2.362
2.343
2.333
1.564
X : parts per Million : Proton

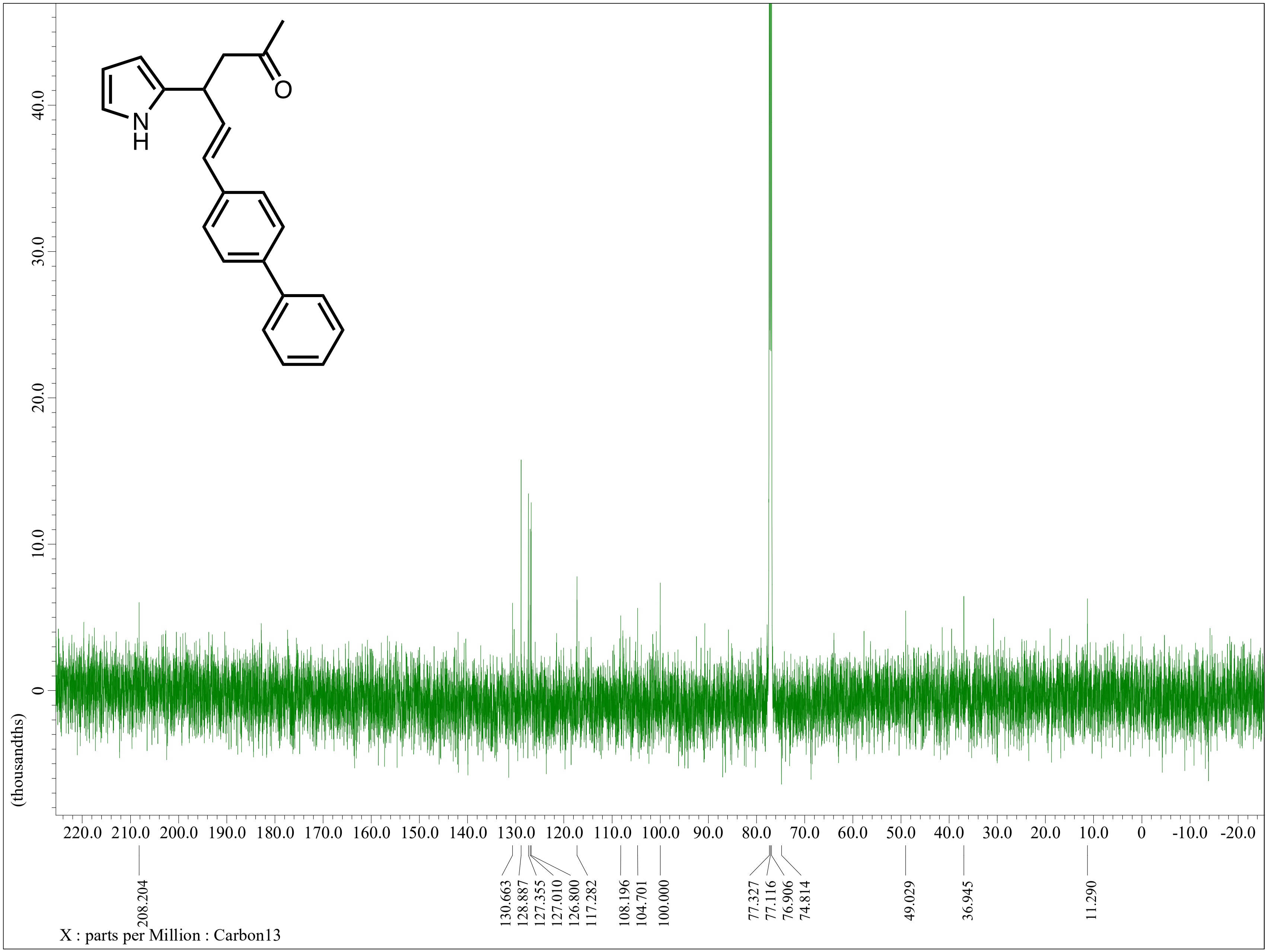

(thousandths)
40.0
30.0
20.0
10.0
0
220.0 210.0 200.0 190.0 180.0 170.0 160.0 150.0 140.0 130.0 120.0 110.0 100.0 90.0 80.0 70.0 60.0 50.0 40.0 30.0 20.0 10.0 0 -10.0 -20.0
208.204
130.663
128.887
127.355
127.010
126.800
117.282
108.196
104.701
100.000
77.327
77.116
76.906
74.814
49.029
36.945
11.290
X : parts per Million : Carbon13

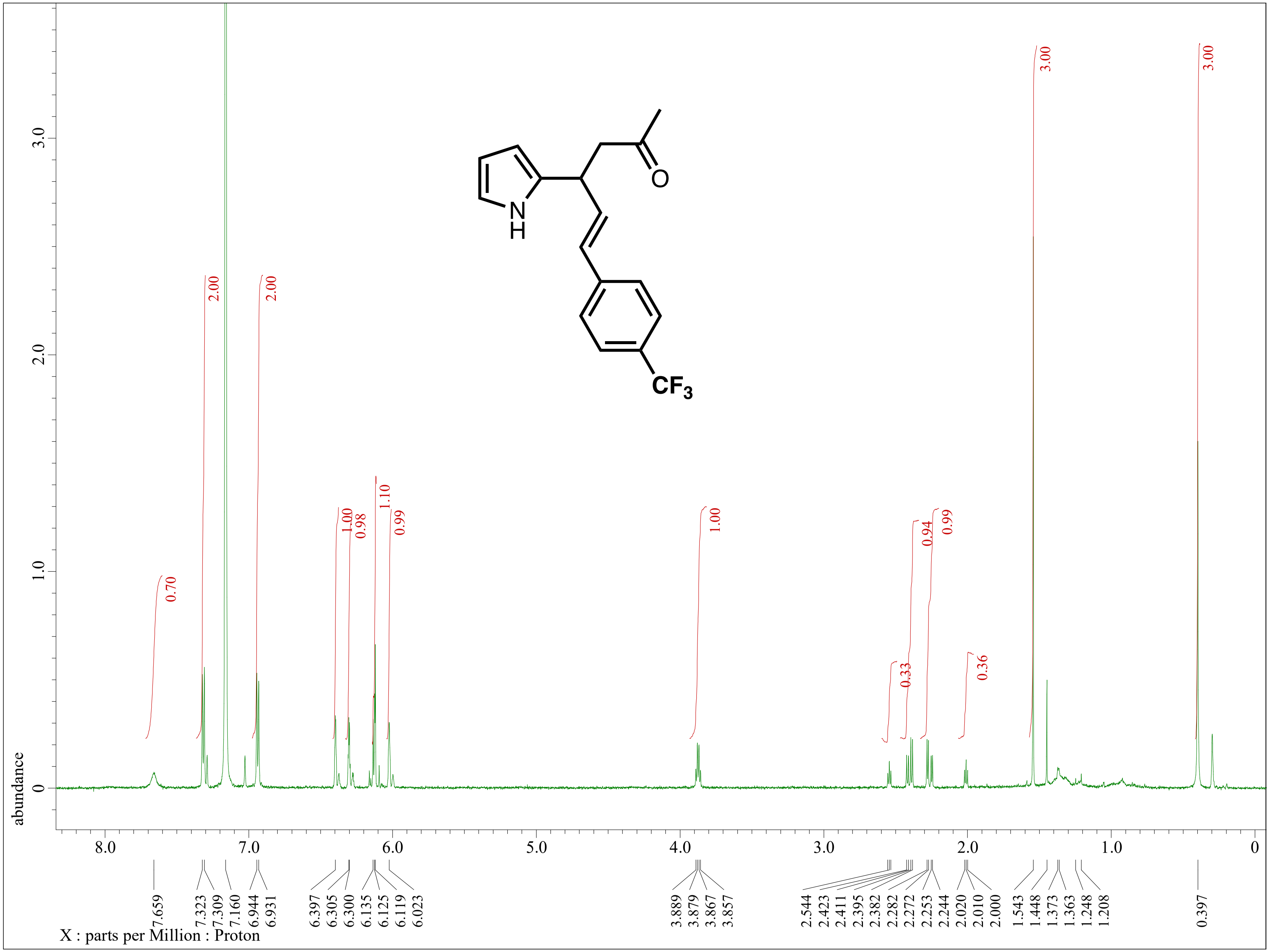

NH
O
CF3
3.00
3.00
2.00
2.00
0.70
1.00
0.98
1.10
0.99
1.00
0.94
0.99
0.33
0.36
7.659
7.323
7.309
7.160
6.944
6.931
6.397
6.305
6.300
6.135
6.125
6.119
6.023
3.889
3.879
3.867
3.857
2.544
2.423
2.411
2.395
2.382
2.282
2.272
2.253
2.244
2.020
2.010
2.000
1.543
1.448
1.373
1.363
1.248
1.208
0.397
abundance
X : parts per Million : Proton

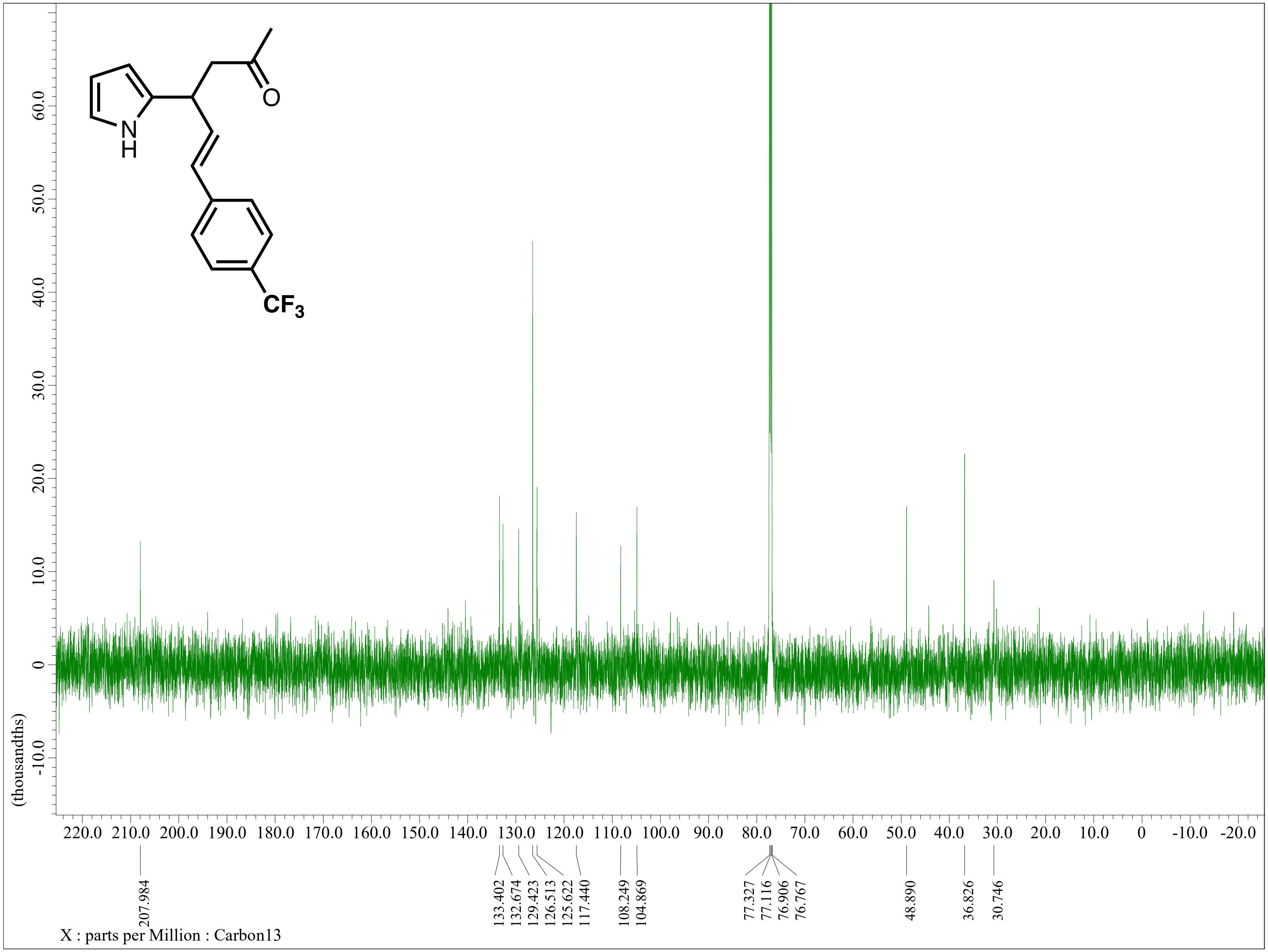

N
H
O
CF3
207.984
133.402
132.674
129.423
126.513
125.622
117.440
108.249
104.869
77.327
77.116
76.906
76.767
48.890
36.826
30.746
220.0 210.0 200.0 190.0 180.0 170.0 160.0 150.0 140.0 130.0 120.0 110.0 100.0 90.0 80.0 70.0 60.0 50.0 40.0 30.0 20.0 10.0 0 -10.0 -20.0
X : parts per Million : Carbon13
60.0 50.0 40.0 30.0 20.0 10.0 0 -10.0
(thousandths)

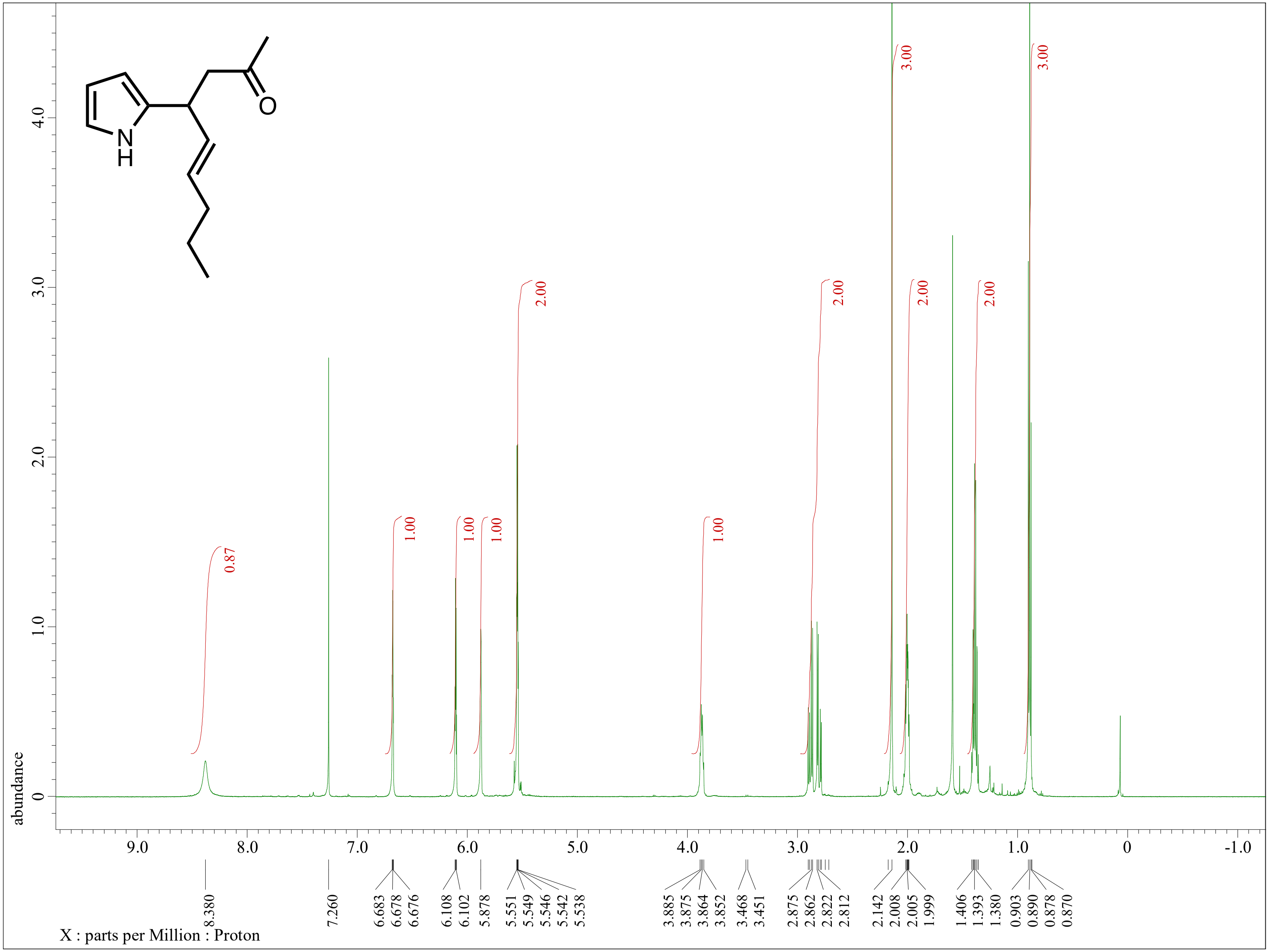

abundance
4.0
3.0
2.0
1.0
0
9.0
8.0
7.0
6.0
5.0
4.0
3.0
2.0
1.0
0
-1.0
X : parts per Million : Proton
0.87
1.00
1.00
1.00
2.00
1.00
2.00
3.00
2.00
2.00
3.00
8.380
7.260
6.683
6.678
6.676
6.108
6.102
5.878
5.551
5.549
5.546
5.542
5.538
3.885
3.875
3.864
3.852
3.468
3.451
2.875
2.862
2.822
2.812
2.142
2.008
2.005
1.999
1.406
1.393
1.380
0.903
0.890
0.878
0.870

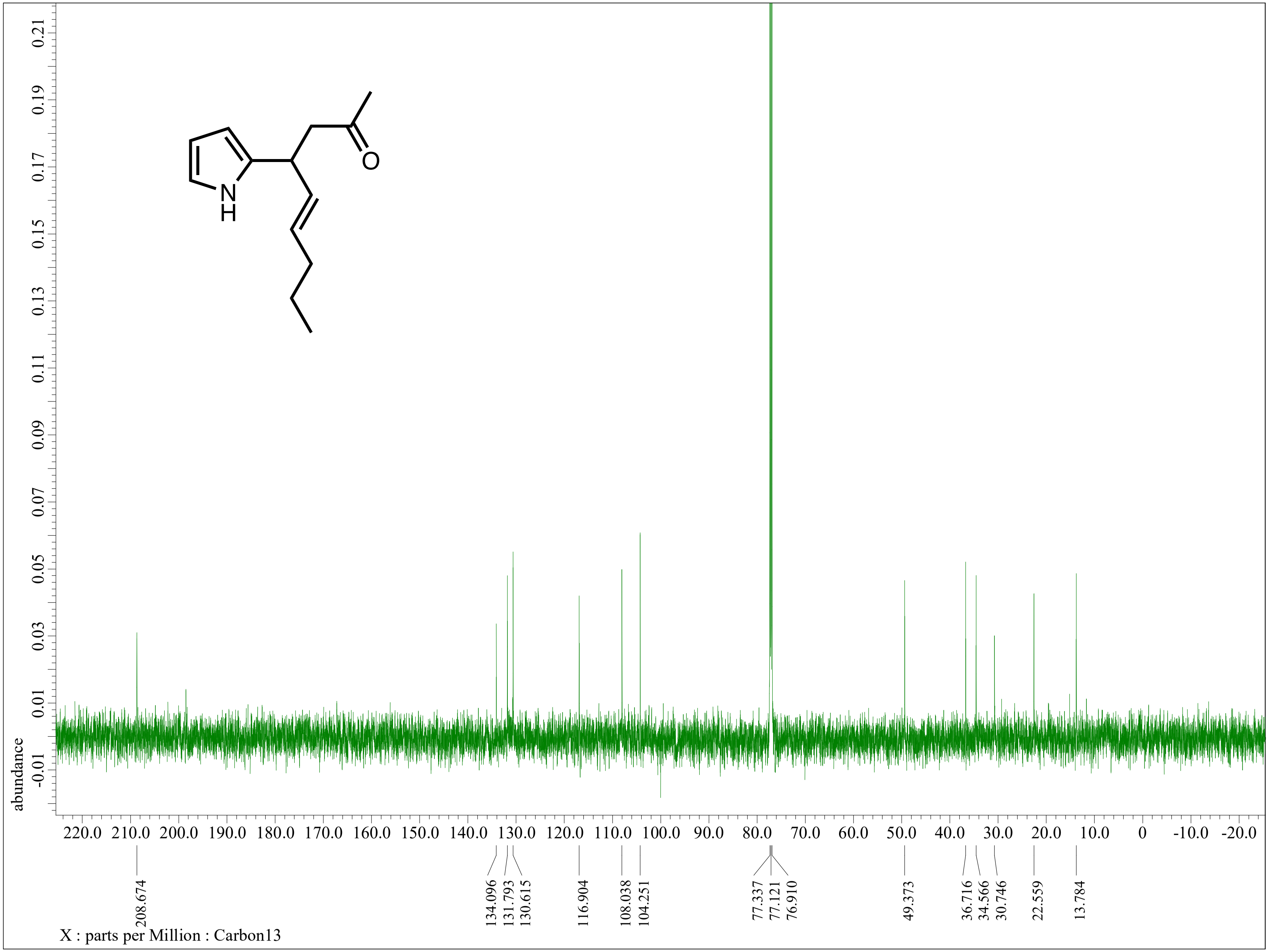

N
H
O
abundance
0.21
0.19
0.17
0.15
0.13
0.11
0.09
0.07
0.05
0.03
0.01
-0.01
220.0 210.0 200.0 190.0 180.0 170.0 160.0 150.0 140.0 130.0 120.0 110.0 100.0 90.0 80.0 70.0 60.0 50.0 40.0 30.0 20.0 10.0 0 -10.0 -20.0
208.674
134.096
131.793
130.615
116.904
108.038
104.251
77.337
77.121
76.910
49.373
36.716
34.566
30.746
22.559
13.784
X : parts per Million : Carbon13

==== Shimadzu LCsolution Analysis Report ====

C:\Documents and Settings\User\Desktop\amy\METHODS 2020\AEB-IV-117-90%-1.lcd

Acquired by	: Admin
Sample Name	: AEB-IV-117-90%-1
Sample ID	: AEB-IV-117-90%-1
Tray#	: 1
Vail #	: 21
Injection Volume	: 10 uL
Data File Name	: AEB-IV-117-90%-1.lcd
Method File Name	: pos3-90%_10MIN_1_d2.lcm
Batch File Name	: Batch table C3 _90%_10min_1.0_D2.lcb
Report File Name	: Default.lcr
Data Acquired	: 7/27/2020 11:10:11 PM
Data Processed	: 7/27/2020 11:20:13 PM

<Chromatogram>

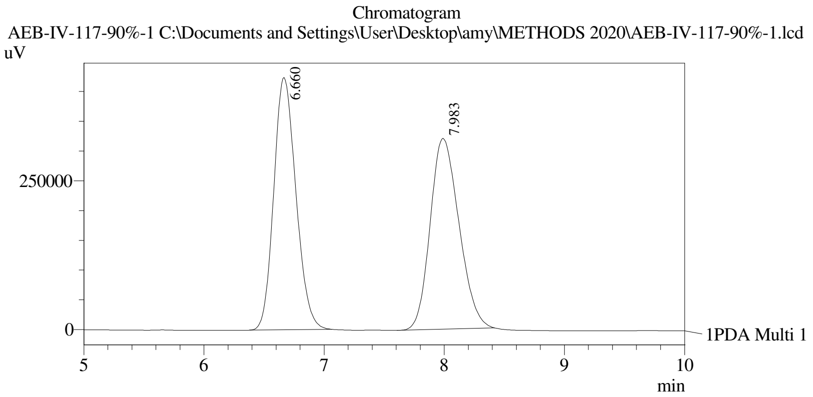

1 PDA Multi 1 / 254nm 4nm

PeakTable

PDA Ch1 254nm 4nm

Peak#	Ret. Time	Area	Height	Area %	Height %
1	6.660	5441985	424203	50.457	56.947
2	7.983	5343393	320699	49.543	43.053
Total		10785378	744902	100.000	100.000

==== Shimadzu LCsolution Analysis Report ====

C:\Documents and Settings\User\Desktop\amy\METHODS 2020\AEB-IV-118-90%-1.lcd

Acquired by	: Admin
Sample Name	: AEB-IV-118-90%-1
Sample ID	: AEB-IV-118-90%-1
Tray#	: 1
Vail #	: 22
Injection Volume	: 10 uL
Data File Name	: AEB-IV-118-90%-1.lcd
Method File Name	: pos3-90%_10MIN_1_d2.lcm
Batch File Name	: Batch table C3 _90%_10min_1.0_D2.lcb
Report File Name	: Default.lcr
Data Acquired	: 7/27/2020 11:30:48 PM
Data Processed	: 7/27/2020 11:40:50 PM

<Chromatogram>

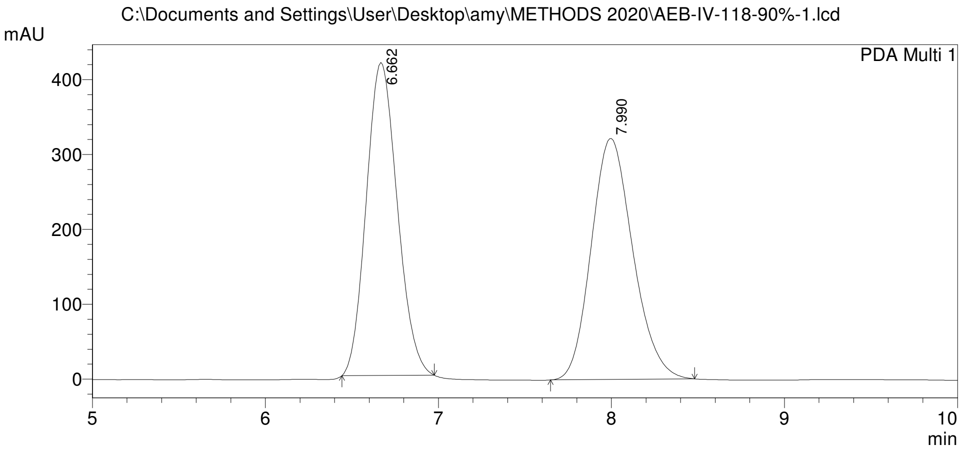

1 PDA Multi 1/254nm 4nm

PeakTable

PDA Ch1 254nm 4nm

Peak#	Ret. Time	Area	Height	Area %	Height %
1	6.662	5226896	418077	49.293	56.486
2	7.990	5376940	322061	50.707	43.514
Total		10603836	740138	100.000	100.000

==== Shimadzu LCsolution Analysis Report ====

C:\Documents and Settings\User\Desktop\amy\METHODS 2020\AEB-IV-59-90%-1.lcd

Acquired by	: Admin
Sample Name	: AEB-IV-59-90%-1
Sample ID	: AEB-IV-59-90%-1
Tray#	: 1
Vail #	: 14
Injection Volume	: 10 uL
Data File Name	: AEB-IV-59-90%-1.lcd
Method File Name	: pos3-90%_10MIN_1_d2.lcm
Batch File Name	: Batch table C3 _90%_10min_1.0_D2.lcb
Report File Name	: Default.lcr
Data Acquired	: 7/27/2020 12:49:46 PM
Data Processed	: 7/27/2020 12:59:48 PM

<Chromatogram>

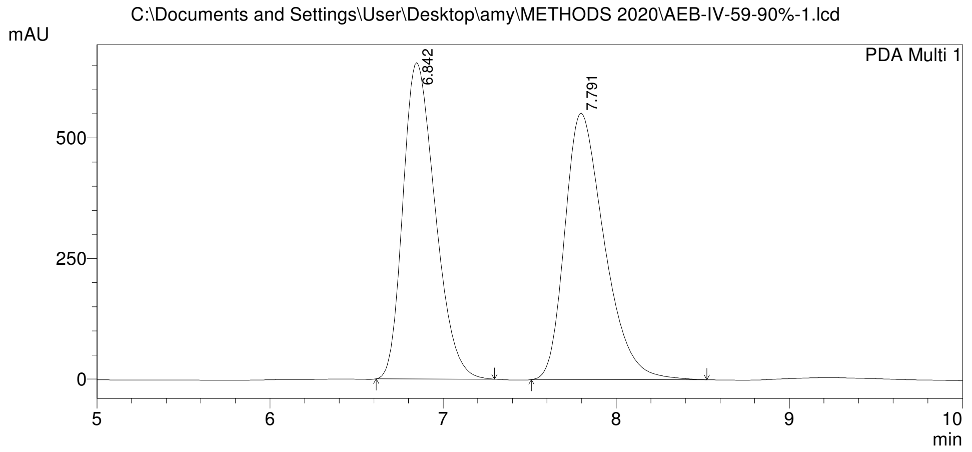

1 PDA Multi 1/254nm 4nm

PeakTable

PDA Ch1 254nm 4nm

Peak#	Ret. Time	Area	Height	Area %	Height %
1	6.842	8479150	656107	49.145	54.267
2	7.791	8774148	552924	50.855	45.733
Total		17253298	1209031	100.000	100.000

==== Shimadzu LCsolution Analysis Report ====

C:\Documents and Settings\User\Desktop\amy\METHODS 2020\AEB-IV-60-90%-1.lcd

Acquired by	: Admin
Sample Name	: AEB-IV-60-90%-1
Sample ID	: AEB-IV-60-90%-1
Tray#	: 1
Vail #	: 1
Injection Volume	: 10 uL
Data File Name	: AEB-IV-60-90%-1.lcd
Method File Name	: pos3-90%_10MIN_1_d2.lcm
Batch File Name	: Batch table C3 _90%_10min_1.0_D2.lcb
Report File Name	: Default.lcr
Data Acquired	: 7/27/2020 4:17:48 PM
Data Processed	: 7/27/2020 4:27:50 PM

<Chromatogram>

C:\Documents and Settings\User\Desktop\amy\METHODS 2020\AEB-IV-60-90%-1.lcd

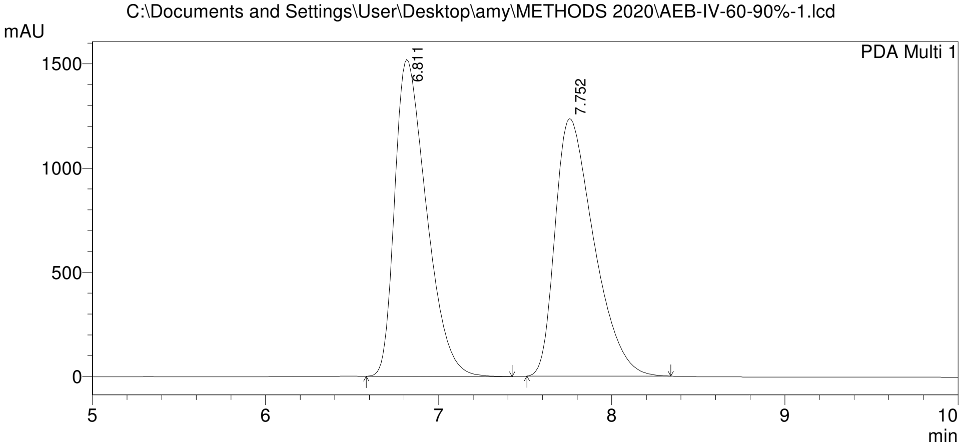

1 PDA Multi 1/254nm 4nm

PeakTable

PDA Ch1 254nm 4nm

Peak#	Ret. Time	Area	Height	Area %	Height %
1	6.811	19357445	1520254	50.132	55.174
2	7.752	19255273	1235117	49.868	44.826
Total		38612718	2755371	100.000	100.000

==== Shimadzu LCsolution Analysis Report ====

C:\Documents and Settings\User\Desktop\amy\METHODS 2020\AEB-IV-61-90%-1.lcd

Acquired by	: Admin
Sample Name	: AEB-IV-61-90%-1
Sample ID	: AEB-IV-61-90%-1
Tray#	: 1
Vail #	: 2
Injection Volume	: 10 uL
Data File Name	: AEB-IV-61-90%-1.lcd
Method File Name	: pos3-90%_10MIN_1_d2.lcm
Batch File Name	: Batch table C3 _90%_10min_1.0_D2.lcb
Report File Name	: Default.lcr
Data Acquired	: 7/27/2020 4:38:25 PM
Data Processed	: 7/27/2020 4:48:27 PM

<Chromatogram>

C:\Documents and Settings\User\Desktop\amy\METHODS 2020\AEB-IV-61-90%-1.lcd

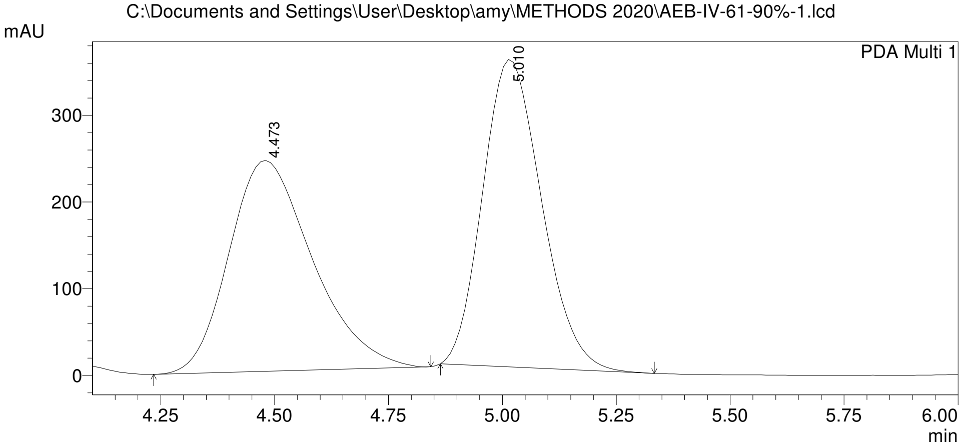

1 PDA Multi 1/254nm 4nm

PeakTable

PDA Ch1 254nm 4nm

Peak#	Ret. Time	Area	Height	Area %	Height %
1	4.473	3100916	243201	49.337	40.708
2	5.010	3184250	354228	50.663	59.292
Total		6285165	597429	100.000	100.000

==== Shimadzu LCsolution Analysis Report ====

C:\Documents and Settings\User\Desktop\amy\METHODS 2020\AEB-IV-62-90%-1.lcd

Acquired by	: Admin
Sample Name	: AEB-IV-62-90%-1
Sample ID	: AEB-IV-62-90%-1
Tray#	: 1
Vail #	: 3
Injection Volume	: 10 uL
Data File Name	: AEB-IV-62-90%-1.lcd
Method File Name	: pos3-90%_10MIN_1_d2.lcm
Batch File Name	: Batch table C3 _90%_10min_1.0_D2.lcb
Report File Name	: Default.lcr
Data Acquired	: 7/27/2020 4:59:03 PM
Data Processed	: 7/27/2020 5:09:06 PM

<Chromatogram>

C:\Documents and Settings\User\Desktop\amy\METHODS 2020\AEB-IV-62-90%-1.lcd

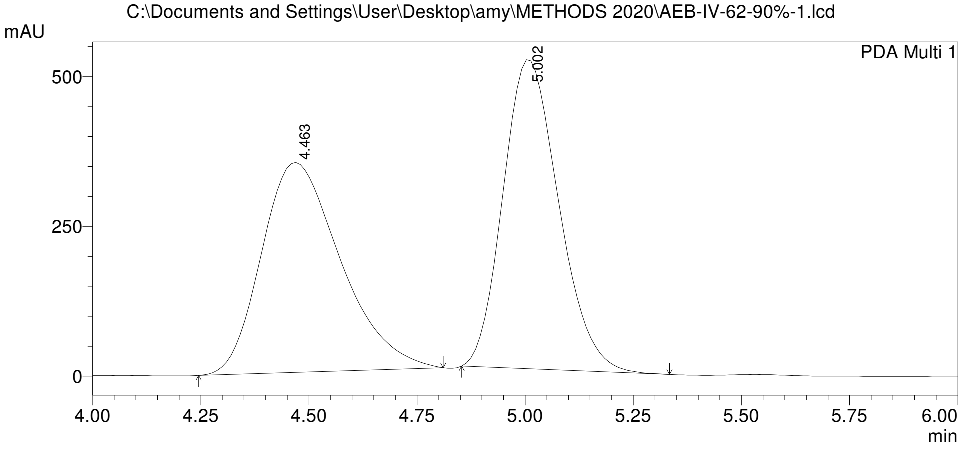

1 PDA Multi 1/254nm 4nm

PeakTable

PDA Ch1 254nm 4nm

Peak#	Ret. Time	Area	Height	Area %	Height %
1	4.463	4456961	350492	49.081	40.462
2	5.002	4623820	515740	50.919	59.538
Total		9080781	866232	100.000	100.000

==== Shimadzu LCsolution Analysis Report ====

C:\Documents and Settings\User\Desktop\amy\METHODS 2020\AEB-IV-63-90%-1.lcd

Acquired by	: Admin
Sample Name	: AEB-IV-63-90%-1
Sample ID	: AEB-IV-63-90%-1
Tray#	: 1
Vail #	: 4
Injection Volume	: 10 uL
Data File Name	: AEB-IV-63-90%-1.lcd
Method File Name	: pos3-90%_10MIN_1_d2.lcm
Batch File Name	: Batch table C3 _90%_10min_1.0_D2.lcb
Report File Name	: Default.lcr
Data Acquired	: 7/27/2020 5:19:40 PM
Data Processed	: 7/27/2020 5:29:42 PM

<Chromatogram>

C:\Documents and Settings\User\Desktop\amy\METHODS 2020\AEB-IV-63-90%-1.lcd

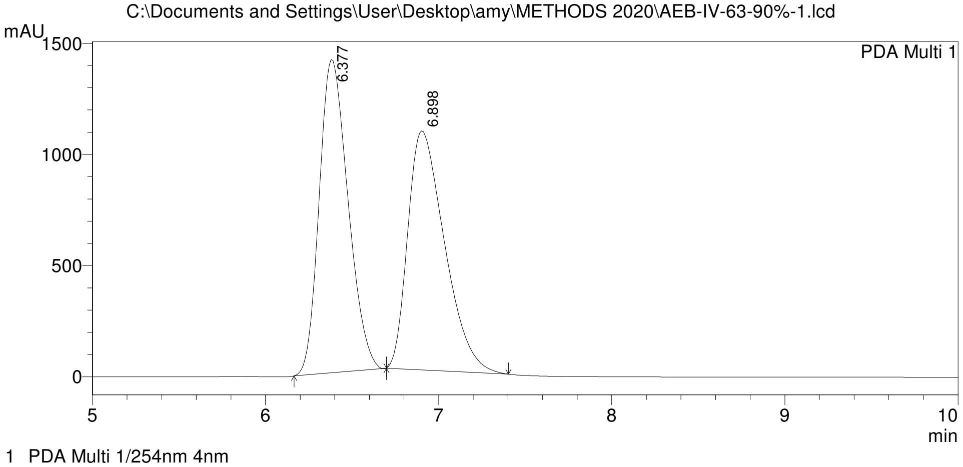

1 PDA Multi 1/254nm 4nm

PeakTable

PDA Ch1 254nm 4nm

Peak#	Ret. Time	Area	Height	Area %	Height %
1	6.377	16303407	1409539	50.678	56.723
2	6.898	15866906	1075393	49.322	43.277
Total		32170313	2484931	100.000	100.000

==== Shimadzu LCsolution Analysis Report ====

C:\Documents and Settings\User\Desktop\amy\METHODS 2020\AEB-IV-64-90%-1.lcd

Acquired by	: Admin
Sample Name	: AEB-IV-64-90%-1
Sample ID	: AEB-IV-64-90%-1
Tray#	: 1
Vail #	: 5
Injection Volume	: 10 uL
Data File Name	: AEB-IV-64-90%-1.lcd
Method File Name	: pos3-90%_10MIN_1_d2.lcm
Batch File Name	: Batch table C3 _90%_10min_1.0_D2.lcb
Report File Name	: Default.lcr
Data Acquired	: 7/27/2020 5:40:19 PM
Data Processed	: 7/27/2020 5:50:21 PM

<Chromatogram>

C:\Documents and Settings\User\Desktop\amy\METHODS 2020\AEB-IV-64-90%-1.lcd

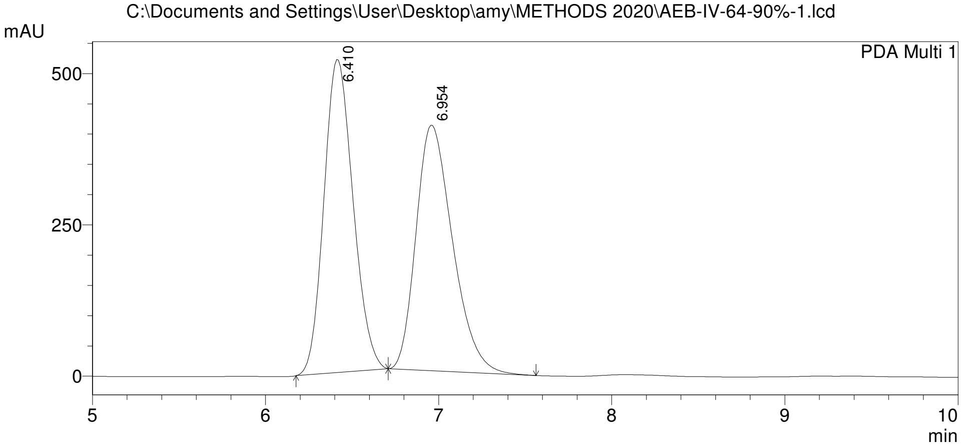

1 PDA Multi 1/254nm 4nm

PeakTable

PDA Ch1 254nm 4nm

Peak#	Ret. Time	Area	Height	Area %	Height %
1	6.410	6058018	517514	50.445	56.059
2	6.954	5951202	405650	49.555	43.941
Total		12009220	923164	100.000	100.000

==== Shimadzu LCsolution Analysis Report ====

C:\Documents and Settings\User\Desktop\amy\METHODS 2020\AEB-IV-65-90%-1.lcd

Acquired by	: Admin
Sample Name	: AEB-IV-65-90%-1
Sample ID	: AEB-IV-65-90%-1
Tray#	: 1
Vail #	: 6
Injection Volume	: 10 uL
Data File Name	: AEB-IV-65-90%-1.lcd
Method File Name	: pos3-90%_10MIN_1_d2.lcm
Batch File Name	: Batch table C3 _90%_10min_1.0_D2.lcb
Report File Name	: Default.lcr
Data Acquired	: 7/27/2020 6:00:55 PM
Data Processed	: 7/27/2020 6:10:58 PM

<Chromatogram>

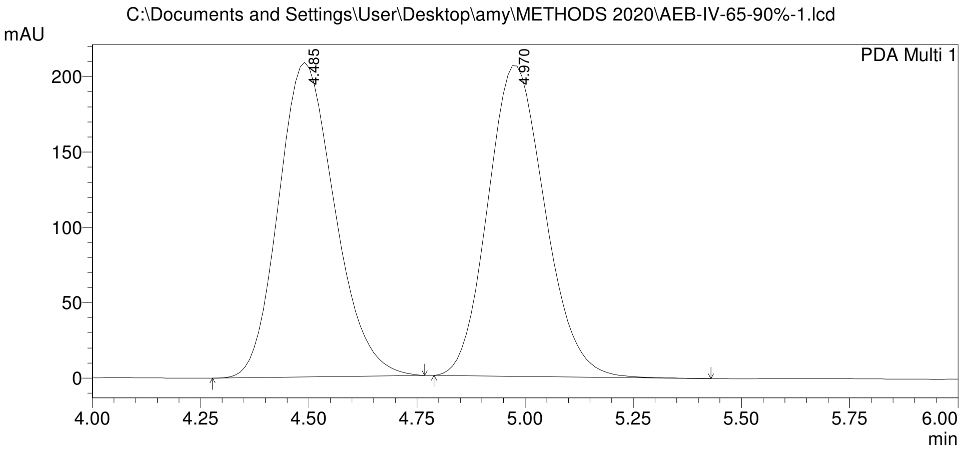

1 PDA Multi 1/254nm 4nm

PeakTable

PDA Ch1 254nm 4nm

Peak#	Ret. Time	Area	Height	Area %	Height %
1	4.485	1891448	208811	49.935	50.317
2	4.970	1896363	206184	50.065	49.683
Total		3787810	414995	100.000	100.000

==== Shimadzu LCsolution Analysis Report ====

C:\Documents and Settings\User\Desktop\amy\METHODS 2020\AEB-IV-66-90%-1.lcd

Acquired by	: Admin
Sample Name	: AEB-IV-66-90%-1
Sample ID	: AEB-IV-66-90%-1
Tray#	: 1
Vail #	: 7
Injection Volume	: 10 uL
Data File Name	: AEB-IV-66-90%-1.lcd
Method File Name	: pos3-90%_10MIN_1_d2.lcm
Batch File Name	: Batch table C3 _90%_10min_1.0_D2.lcb
Report File Name	: Default.lcr
Data Acquired	: 7/27/2020 6:21:32 PM
Data Processed	: 7/27/2020 6:31:34 PM

<Chromatogram>

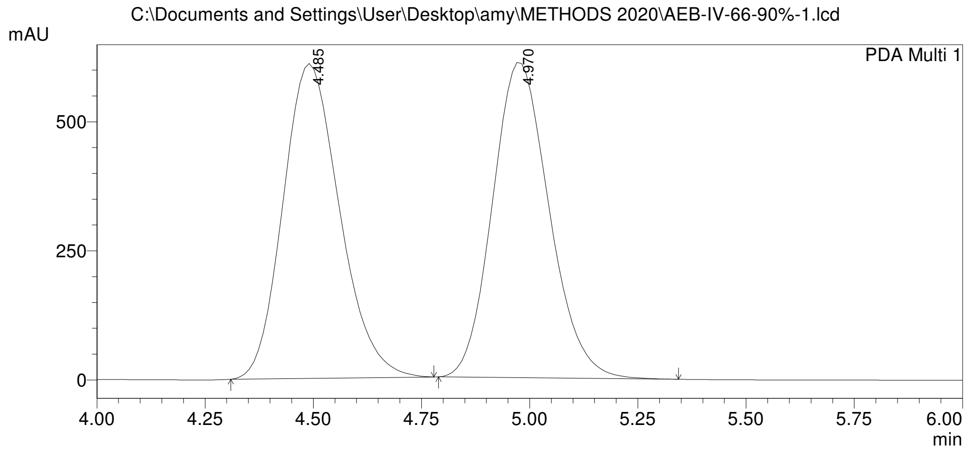

1 PDA Multi 1/254nm 4nm

PeakTable

PDA Ch1 254nm 4nm

Peak#	Ret. Time	Area	Height	Area %	Height %
1	4.485	5544195	609680	49.930	49.975
2	4.970	5559825	610285	50.070	50.025
Total		11104020	1219965	100.000	100.000

==== Shimadzu LCsolution Analysis Report ====

C:\Documents and Settings\User\Desktop\amy\METHODS 2020\AEB-IV-67-90%-1.lcd

Acquired by	: Admin
Sample Name	: AEB-IV-67-90%-1
Sample ID	: AEB-IV-67-90%-1
Tray#	: 1
Vail #	: 8
Injection Volume	: 10 uL
Data File Name	: AEB-IV-67-90%-1.lcd
Method File Name	: pos3-90%_10MIN_1_d2.lcm
Batch File Name	: Batch table C3 _90%_10min_1.0_D2.lcb
Report File Name	: Default.lcr
Data Acquired	: 7/27/2020 6:42:08 PM
Data Processed	: 7/27/2020 6:52:11 PM

<Chromatogram>

C:\Documents and Settings\User\Desktop\amy\METHODS 2020\AEB-IV-67-90%-1.lcd

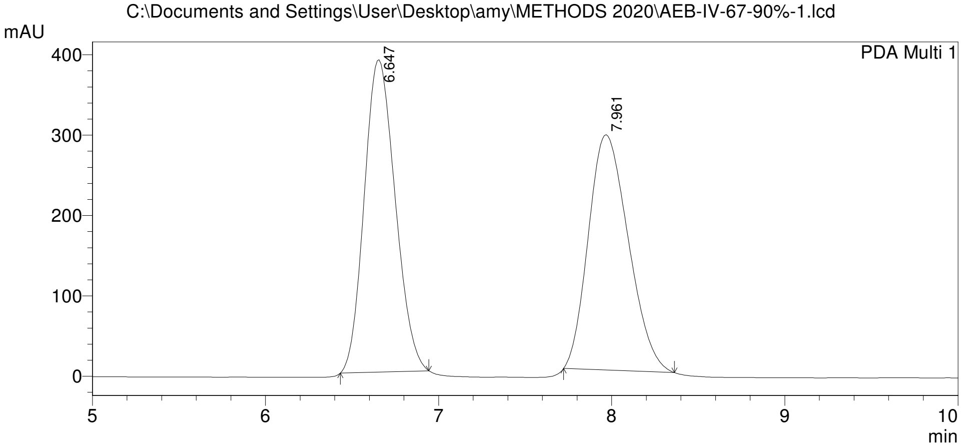

1 PDA Multi 1/254nm 4nm

PeakTable

PDA Ch1 254nm 4nm

Peak#	Ret. Time	Area	Height	Area %	Height %
1	6.647	4847730	388665	50.777	57.034
2	7.961	4699288	292791	49.223	42.966
Total		9547019	681456	100.000	100.000

==== Shimadzu LCsolution Analysis Report ====

C:\Documents and Settings\User\Desktop\amy\METHODS 2020\AEB-IV-68-90%-1.lcd

Acquired by	: Admin
Sample Name	: AEB-IV-68-90%-1
Sample ID	: AEB-IV-68-90%-1
Tray#	: 1
Vail #	: 9
Injection Volume	: 10 uL
Data File Name	: AEB-IV-68-90%-1.lcd
Method File Name	: pos3-90%_10MIN_1_d2.lcm
Batch File Name	: Batch table C3 _90%_10min_1.0_D2.lcb
Report File Name	: Default.lcr
Data Acquired	: 7/27/2020 7:02:45 PM
Data Processed	: 7/27/2020 7:12:47 PM

<Chromatogram>

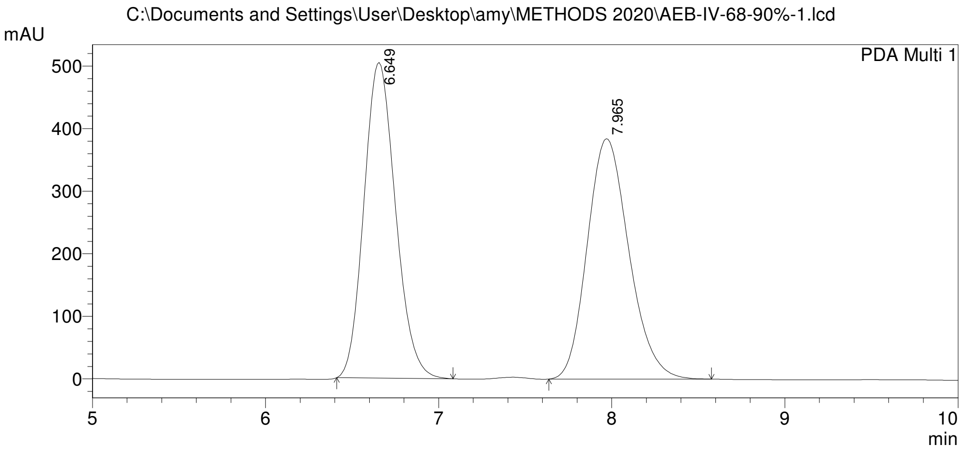

1 PDA Multi 1/254nm 4nm

PeakTable

PDA Ch1 254nm 4nm

Peak#	Ret. Time	Area	Height	Area %	Height %
1	6.649	6409803	504382	49.952	56.760
2	7.965	6422037	384241	50.048	43.240
Total		12831840	888623	100.000	100.000

==== Shimadzu LCsolution Analysis Report ====

C:\Documents and Settings\User\Desktop\amy\METHODS 2020\AEB-IV-70-90%-1.lcd

Acquired by	: Admin
Sample Name	: AEB-IV-70-90%-1
Sample ID	: AEB-IV-70-90%-1
Tray#	: 1
Vail #	: 10
Injection Volume	: 10 uL
Data File Name	: AEB-IV-70-90%-1.lcd
Method File Name	: pos3-90%_10MIN_1_d2.lcm
Batch File Name	: Batch table C3 _90%_10min_1.0_D2.lcb
Report File Name	: Default.lcr
Data Acquired	: 7/27/2020 7:23:24 PM
Data Processed	: 7/27/2020 7:33:25 PM

<Chromatogram>

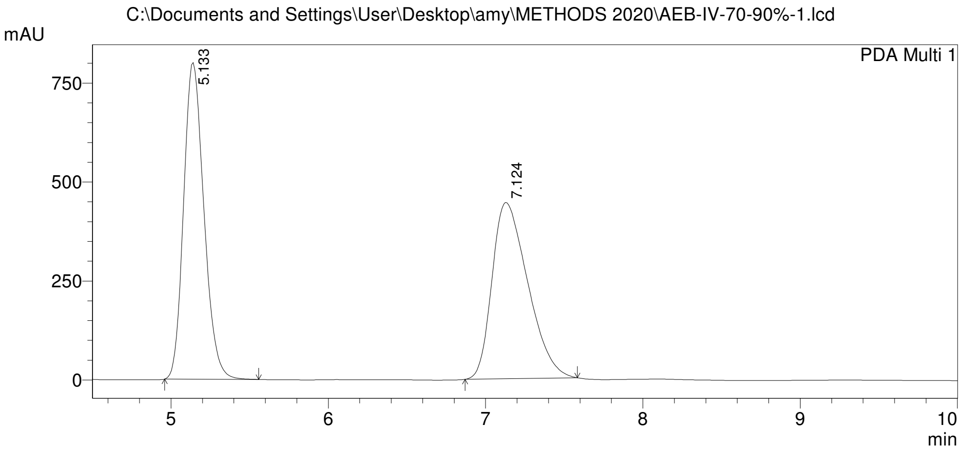

1 PDA Multi 1/254nm 4nm

PeakTable

PDA Ch1 254nm 4nm

Peak#	Ret. Time	Area	Height	Area %	Height %
1	5.133	7172623	799808	50.511	64.225
2	7.124	7027607	445506	49.489	35.775
Total		14200230	1245314	100.000	100.000

==== Shimadzu LCsolution Analysis Report ====

C:\Documents and Settings\User\Desktop\amy\METHODS 2020\AEB-IV-78-90%-1.lcd

Acquired by	: Admin
Sample Name	: AEB-IV-78-90%-1
Sample ID	: AEB-IV-78-90%-1
Tray#	: 1
Vail #	: 11
Injection Volume	: 10 uL
Data File Name	: AEB-IV-78-90%-1.lcd
Method File Name	: pos3-90%_10MIN_1_d2.lcm
Batch File Name	: Batch table C3 _90%_10min_1.0_D2.lcb
Report File Name	: Default.lcr
Data Acquired	: 7/27/2020 7:44:01 PM
Data Processed	: 7/27/2020 7:54:03 PM

<Chromatogram>

C:\Documents and Settings\User\Desktop\amy\METHODS 2020\AEB-IV-78-90%-1.lcd

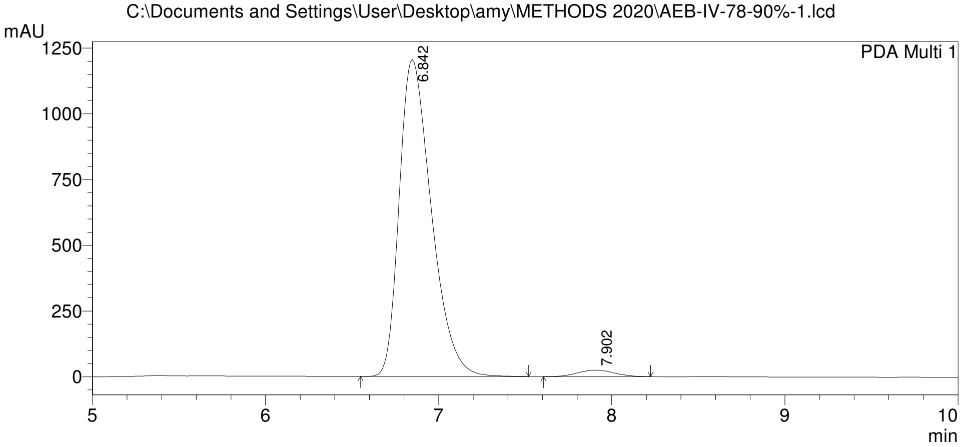

1 PDA Multi 1/254nm 4nm

PeakTable

PDA Ch1 254nm 4nm

Peak#	Ret. Time	Area	Height	Area %	Height %
1	6.842	15529951	1205939	97.688	97.975
2	7.902	367588	24930	2.312	2.025
Total		15897539	1230869	100.000	100.000

==== Shimadzu LCsolution Analysis Report ====

C:\Documents and Settings\User\Desktop\amy\METHODS 2020\AEB-IV-80-90%-1.lcd

Acquired by	: Admin
Sample Name	: AEB-IV-80-90%-1
Sample ID	: AEB-IV-80-90%-1
Tray#	: 1
Vail #	: 12
Injection Volume	: 10 uL
Data File Name	: AEB-IV-80-90%-1.lcd
Method File Name	: pos3-90%_10MIN_1_d2.lcm
Batch File Name	: Batch table C3 _90%_10min_1.0_D2.lcb
Report File Name	: Default.lcr
Data Acquired	: 7/27/2020 8:04:38 PM
Data Processed	: 7/27/2020 8:14:41 PM

<Chromatogram>

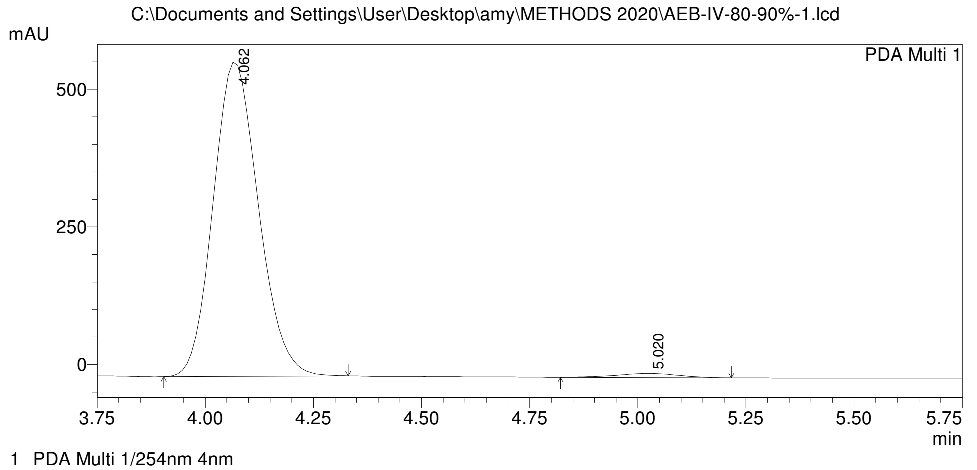

1 PDA Multi 1/254nm 4nm

PeakTable

PDA Ch1 254nm 4nm

Peak#	Ret. Time	Area	Height	Area %	Height %
1	4.062	4157636	570616	98.234	98.676
2	5.020	74744	7655	1.766	1.324
Total		4232380	578272	100.000	100.000

==== Shimadzu LCsolution Analysis Report ====

C:\Documents and Settings\User\Desktop\amy\METHODS 2020\AEB-IV-88-90%-1.lcd

Acquired by	: Admin
Sample Name	: AEB-IV-88-90%-1
Sample ID	: AEB-IV-88-90%-1
Tray#	: 1
Vail #	: 20
Injection Volume	: 10 uL
Data File Name	: AEB-IV-88-90%-1.lcd
Method File Name	: pos3-90%_10MIN_1_d2.lcm
Batch File Name	: Batch table C3 _90%_10min_1.0_D2.lcb
Report File Name	: Default.lcr
Data Acquired	: 7/27/2020 10:49:34 PM
Data Processed	: 7/27/2020 10:59:37 PM

<Chromatogram>

C:\Documents and Settings\User\Desktop\amy\METHODS 2020\AEB-IV-88-90%-1.lcd

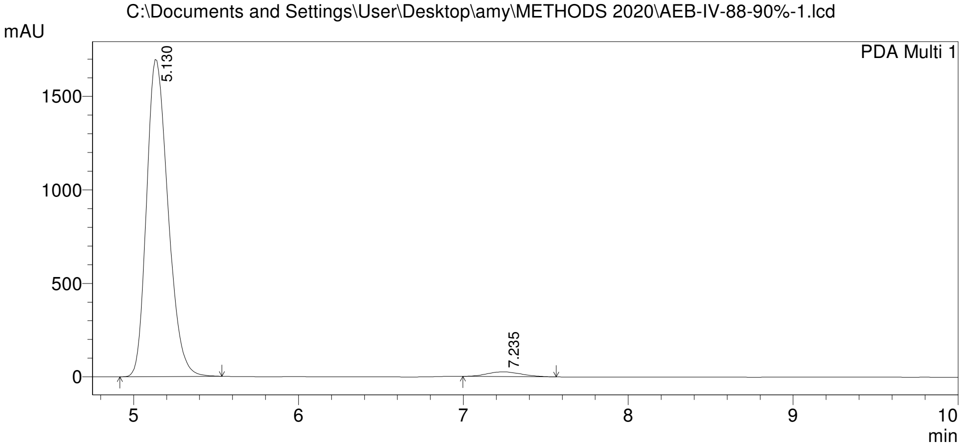

1 PDA Multi 1/254nm 4nm

PeakTable

PDA Ch1 254nm 4nm

Peak#	Ret. Time	Area	Height	Area %	Height %
1	5.130	15379390	1696473	97.714	98.530
2	7.235	359783	25304	2.286	1.470
Total		15739174	1721778	100.000	100.000

==== Shimadzu LCsolution Analysis Report ====

C:\Documents and Settings\User\Desktop\amy\METHODS 2020\AEB-IV-87-90%-1.lcd

Acquired by : Admin
Sample Name : AEB-IV-87-90%-1
Sample ID : AEB-IV-87-90%-1
Tray# : 1
Vail # : 19
Injection Volume : 10 uL
Data File Name : AEB-IV-87-90%-1.lcd
Method File Name : pos3-90%_10MIN_1_d2.lcm
Batch File Name : Batch table C3 _90%_10min_1.0_D2.lcb
Report File Name : Default.lcr
Data Acquired : 7/27/2020 10:28:57 PM
Data Processed : 7/27/2020 10:39:00 PM

<Chromatogram>

C:\Documents and Settings\User\Desktop\amy\METHODS 2020\AEB-IV-87-90%-1.lcd

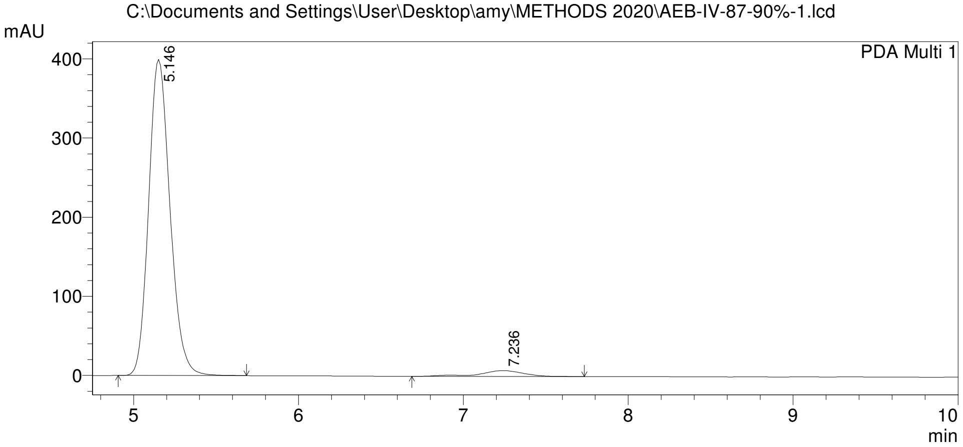

1 PDA Multi 1/254nm 4nm

PeakTable

PDA Ch1 254nm 4nm

Peak#	Ret. Time	Area	Height	Area %	Height %
1	5.146	3598770	399450	96.281	98.191
2	7.236	139009	7361	3.719	1.809
Total		3737780	406811	100.000	100.000

==== Shimadzu LCsolution Analysis Report ====

C:\Documents and Settings\User\Desktop\amy\METHODS 2020\AEB-IV-86-90%-1.lcd

Acquired by	: Admin
Sample Name	: AEB-IV-86-90%-1
Sample ID	: AEB-IV-86-90%-1
Tray#	: 1
Vail #	: 18
Injection Volume	: 10 uL
Data File Name	: AEB-IV-86-90%-1.lcd
Method File Name	: pos3-90%_10MIN_1_d2.lcm
Batch File Name	: Batch table C3 _90%_10min_1.0_D2.lcb
Report File Name	: Default.lcr
Data Acquired	: 7/27/2020 10:08:21 PM
Data Processed	: 7/27/2020 10:18:24 PM

<Chromatogram>

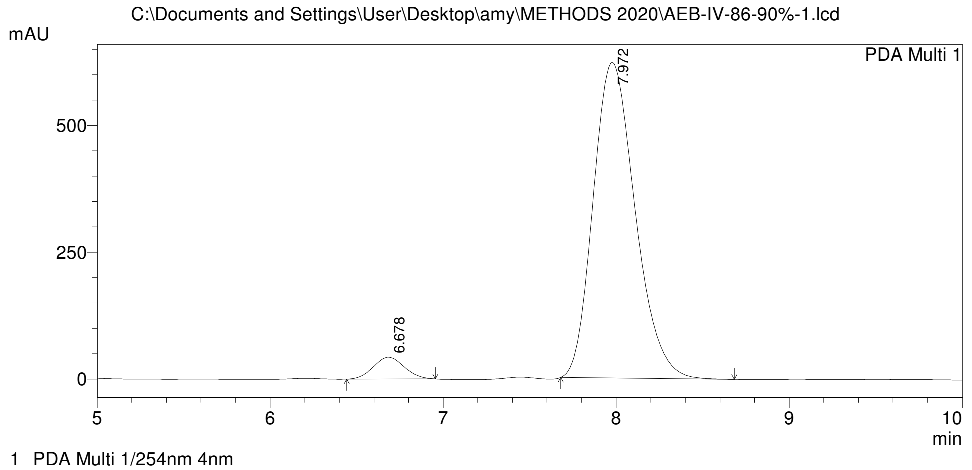

1 PDA Multi 1/254nm 4nm

PeakTable

PDA Ch1 254nm 4nm

Peak#	Ret. Time	Area	Height	Area %	Height %
1	6.678	535867	42947	4.862	6.456
2	7.972	10484817	622329	95.138	93.544
Total		11020684	665276	100.000	100.000

==== Shimadzu LCsolution Analysis Report ====

C:\Documents and Settings\User\Desktop\amy\METHODS 2020\AEB-IV-85-90%-1.lcd

Acquired by	: Admin
Sample Name	: AEB-IV-85-90%-1
Sample ID	: AEB-IV-85-90%-1
Tray#	: 1
Vail #	: 17
Injection Volume	: 10 uL
Data File Name	: AEB-IV-85-90%-1.lcd
Method File Name	: pos3-90%_10MIN_1_d2.lcm
Batch File Name	: Batch table C3 _90%_10min_1.0_D2.lcb
Report File Name	: Default.lcr
Data Acquired	: 7/27/2020 9:47:46 PM
Data Processed	: 7/27/2020 9:57:47 PM

<Chromatogram>

C:\Documents and Settings\User\Desktop\amy\METHODS 2020\AEB-IV-85-90%-1.lcd

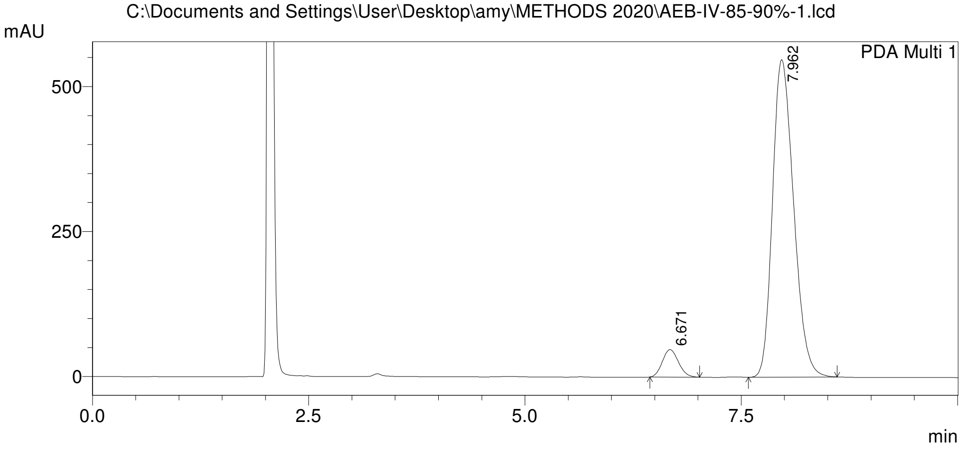

1 PDA Multi 1/254nm 4nm

PeakTable

PDA Ch1 254nm 4nm

Peak#	Ret. Time	Area	Height	Area %	Height %
1	6.671	610949	47344	6.165	7.957
2	7.962	9298609	547628	93.835	92.043
Total		9909558	594972	100.000	100.000

==== Shimadzu LCsolution Analysis Report ====

C:\Documents and Settings\User\Desktop\amy\METHODS 2020\AEB-IV-84-90%-1.lcd

Acquired by	: Admin
Sample Name	: AEB-IV-84-90%-1
Sample ID	: AEB-IV-84-90%-1
Tray#	: 1
Vail #	: 16
Injection Volume	: 10 uL
Data File Name	: AEB-IV-84-90%-1.lcd
Method File Name	: pos3-90%_10MIN_1_d2.lcm
Batch File Name	: Batch table C3 _90%_10min_1.0_D2.lcb
Report File Name	: Default.lcr
Data Acquired	: 7/27/2020 9:27:09 PM
Data Processed	: 7/27/2020 9:37:11 PM

<Chromatogram>

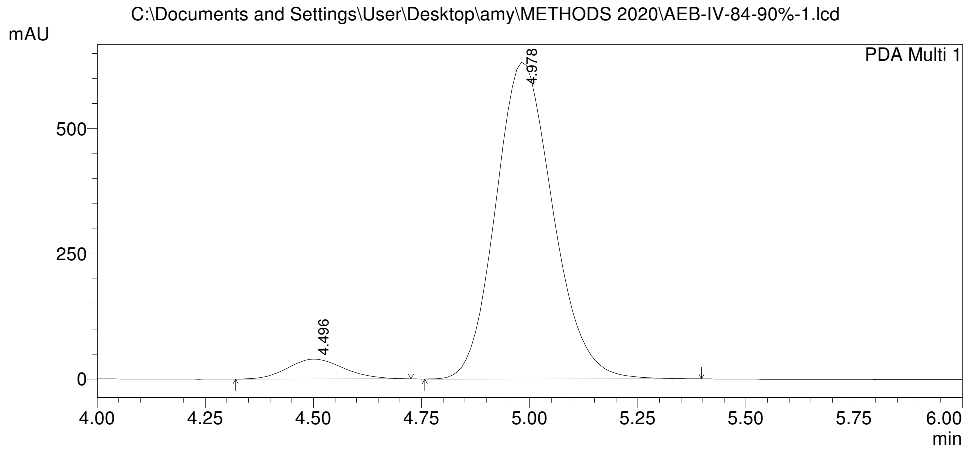

1 PDA Multi 1/254nm 4nm

PeakTable

PDA Ch1 254nm 4nm

Peak#	Ret. Time	Area	Height	Area %	Height %
1	4.496	357312	39678	5.792	5.907
2	4.978	5811359	632004	94.208	94.093
Total		6168670	671682	100.000	100.000

==== Shimadzu LCsolution Analysis Report ====

C:\Documents and Settings\User\Desktop\amy\METHODS 2020\AEB-IV-83-90%-1.lcd

Acquired by	: Admin
Sample Name	: AEB-IV-83-90%-1
Sample ID	: AEB-IV-83-90%-1
Tray#	: 1
Vail #	: 15
Injection Volume	: 10 uL
Data File Name	: AEB-IV-83-90%-1.lcd
Method File Name	: pos3-90%_10MIN_1_d2.lcm
Batch File Name	: Batch table C3 _90%_10min_1.0_D2.lcb
Report File Name	: Default.lcr
Data Acquired	: 7/27/2020 9:06:29 PM
Data Processed	: 7/27/2020 9:16:33 PM

<Chromatogram>

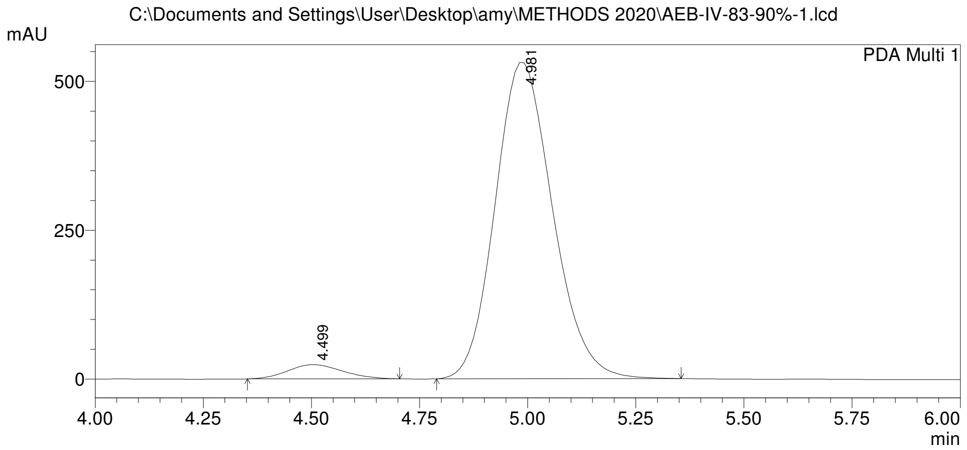

1 PDA Multi 1/254nm 4nm

PeakTable

PDA Ch1 254nm 4nm

Peak#	Ret. Time	Area	Height	Area %	Height %
1	4.499	209235	23806	4.085	4.289
2	4.981	4912857	531207	95.915	95.711
Total		5122092	555013	100.000	100.000

==== Shimadzu LCsolution Analysis Report ====

C:\Documents and Settings\User\Desktop\amy\METHODS 2020\AEB-IV-82-90%-1.lcd

Acquired by	: Admin
Sample Name	: AEB-IV-82-90%-1
Sample ID	: AEB-IV-82-90%-1
Tray#	: 1
Vail #	: 14
Injection Volume	: 10 uL
Data File Name	: AEB-IV-82-90%-1.lcd
Method File Name	: pos3-90%_10MIN_1_d2.lcm
Batch File Name	: Batch table C3 _90%_10min_1.0_D2.lcb
Report File Name	: Default.lcr
Data Acquired	: 7/27/2020 8:45:50 PM
Data Processed	: 7/27/2020 8:55:52 PM

<Chromatogram>

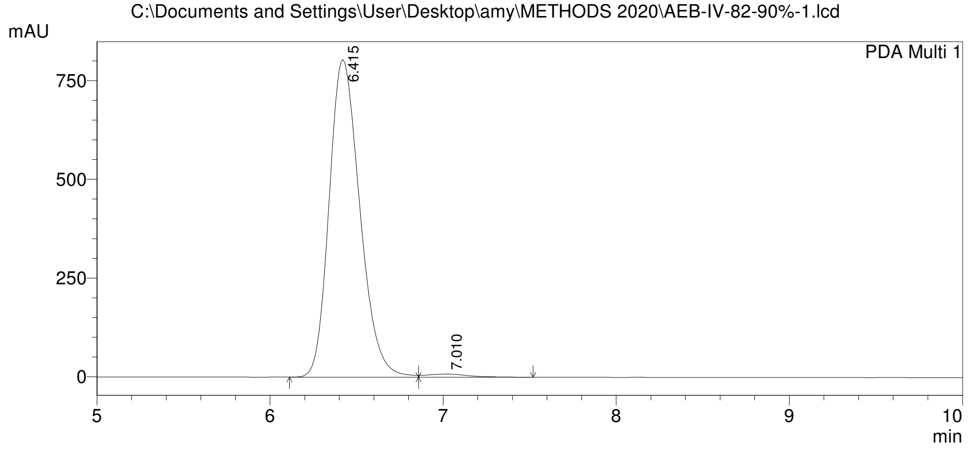

1 PDA Multi 1/254nm 4nm

PeakTable

PDA Ch1 254nm 4nm

Peak#	Ret. Time	Area	Height	Area %	Height %
1	6.415	9903400	804210	98.716	99.028
2	7.010	128861	7895	1.284	0.972
Total		10032262	812104	100.000	100.000

==== Shimadzu LCsolution Analysis Report ====

C:\Documents and Settings\User\Desktop\amy\METHODS 2020\AEB-IV-81-90%-1.lcd

Acquired by	: Admin
Sample Name	: AEB-IV-81-90%-1
Sample ID	: AEB-IV-81-90%-1
Tray#	: 1
Vail #	: 13
Injection Volume	: 10 uL
Data File Name	: AEB-IV-81-90%-1.lcd
Method File Name	: pos3-90%_10MIN_1_d2.lcm
Batch File Name	: Batch table C3 _90%_10min_1.0_D2.lcb
Report File Name	: Default.lcr
Data Acquired	: 7/27/2020 8:25:13 PM
Data Processed	: 7/27/2020 8:35:15 PM

<Chromatogram>

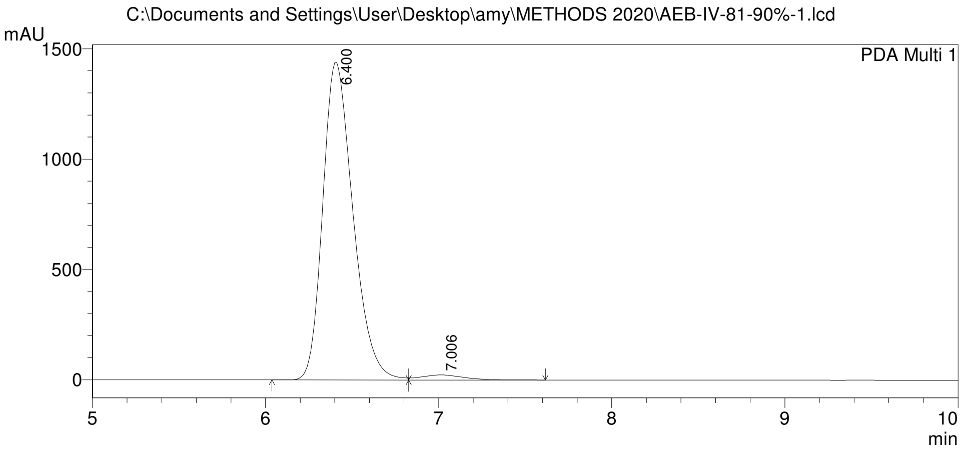

1 PDA Multi 1/254nm 4nm

PeakTable

PDA Ch1 254nm 4nm

Peak#	Ret. Time	Area	Height	Area %	Height %
1	6.400	17474047	1439032	97.823	98.432
2	7.006	388919	22926	2.177	1.568
Total		17862966	1461958	100.000	100.000

==== Shimadzu LCsolution Analysis Report ====

C:\Documents and Settings\User\Desktop\amy\METHODS 2020\AEB-IV-119-90%-1.lcd

Acquired by	: Admin
Sample Name	: AEB-IV-119-90%-1
Sample ID	: AEB-IV-119-90%-1
Tray#	: 1
Vail #	: 23
Injection Volume	: 10 uL
Data File Name	: AEB-IV-119-90%-1.lcd
Method File Name	: pos3-90%_10MIN_1_d2.lcm
Batch File Name	: Batch table C3 _90%_10min_1.0_D2.lcb
Report File Name	: Default.lcr
Data Acquired	: 7/27/2020 11:51:24 PM
Data Processed	: 7/28/2020 12:01:26 AM

<Chromatogram>

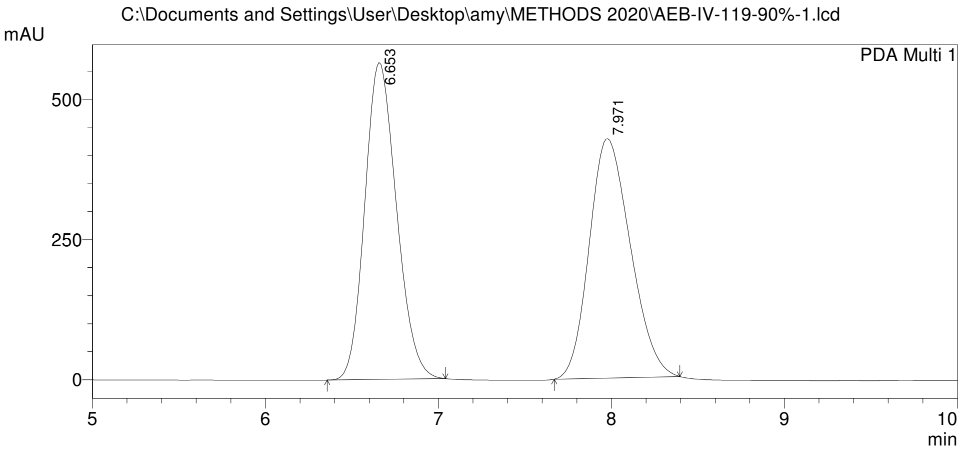

1 PDA Multi 1/254nm 4nm

PeakTable

PDA Ch1 254nm 4nm

Peak#	Ret. Time	Area	Height	Area %	Height %
1	6.653	7242799	566112	50.595	56.959
2	7.971	7072516	427784	49.405	43.041
Total		14315315	993895	100.000	100.000

==== Shimadzu LCsolution Analysis Report ====

C:\Documents and Settings\User\Desktop\amy\METHODS 2020\AEB-IV-120-90%-1.lcd

Acquired by	: Admin
Sample Name	: AEB-IV-120-90%-1
Sample ID	: AEB-IV-120-90%-1
Tray#	: 1
Vail #	: 24
Injection Volume	: 10 uL
Data File Name	: AEB-IV-120-90%-1.lcd
Method File Name	: pos3-90%_10MIN_1_d2.lcm
Batch File Name	: Batch table C3 _90%_10min_1.0_D2.lcb
Report File Name	: Default.lcr
Data Acquired	: 7/28/2020 12:12:00 AM
Data Processed	: 7/28/2020 12:22:02 AM

<Chromatogram>

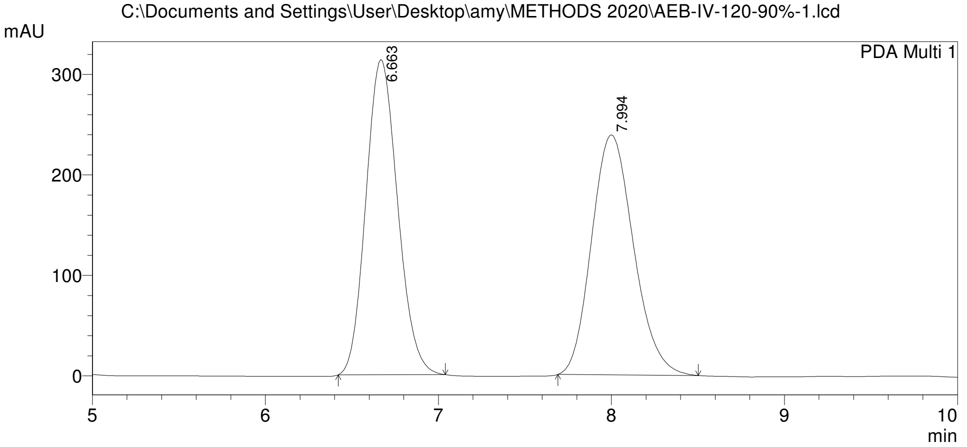

1 PDA Multi 1/254nm 4nm

PeakTable

PDA Ch1 254nm 4nm

Peak#	Ret. Time	Area	Height	Area %	Height %
1	6.663	4012254	313766	49.910	56.781
2	7.994	4026648	238819	50.090	43.219
Total		8038902	552585	100.000	100.000

==== Shimadzu LCsolution Analysis Report ====

C:\Documents and Settings\User\Desktop\amy\METHODS 2020\AEB-IV-129-90%-1.lcd

Acquired by	: Admin
Sample Name	: AEB-IV-129-90%-1
Sample ID	: AEB-IV-129-90%-1
Tray#	: 1
Vail #	: 1
Injection Volume	: 10 uL
Data File Name	: AEB-IV-129-90%-1.lcd
Method File Name	: pos3-90%_10MIN_1_d2.lcm
Batch File Name	: Batch table C3 _90%_10min_1.0_D2.lcb
Report File Name	: Default.lcr
Data Acquired	: 8/10/2020 1:57:58 PM
Data Processed	: 8/10/2020 2:08:01 PM

<Chromatogram>

C:\Documents and Settings\User\Desktop\amy\METHODS 2020\AEB-IV-129-90%-1.lcd

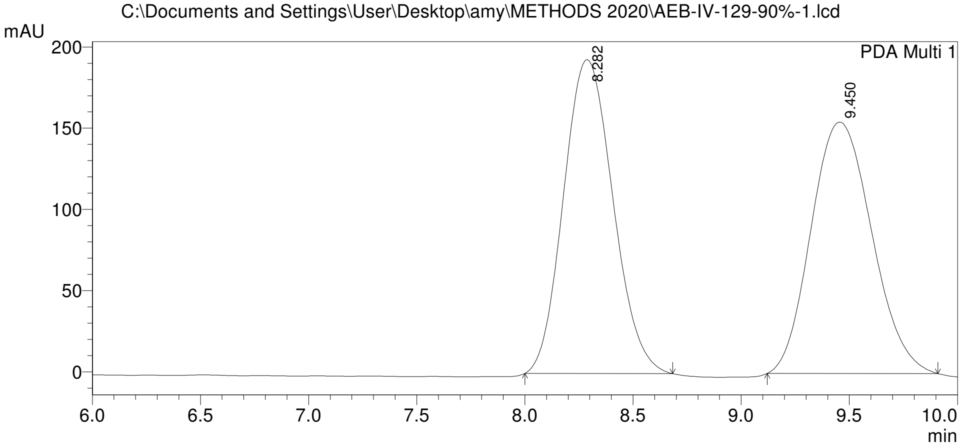

1 PDA Multi 1/254nm 4nm

PeakTable

PDA Ch1 254nm 4nm

Peak#	Ret. Time	Area	Height	Area %	Height %
1	8.282	3072026	193301	50.376	55.531
2	9.450	3026213	154793	49.624	44.469
Total		6098239	348094	100.000	100.000

==== Shimadzu LCsolution Analysis Report ====

C:\Documents and Settings\User\Desktop\amy\METHODS 2020\AEB-IV-132-2-90%-1.lcd

Acquired by	: Admin
Sample Name	: AEB-IV-132-2-90%-1
Sample ID	: AEB-IV-132-2-90%-1
Tray#	: 1
Vail #	: 3
Injection Volume	: 10 uL
Data File Name	: AEB-IV-132-2-90%-1.lcd
Method File Name	: pos3-90%_10MIN_1_d2.lcm
Batch File Name	: Batch table C3 _90%_10min_1.0_D2.lcb
Report File Name	: Default.lcr
Data Acquired	: 8/12/2020 2:39:12 PM
Data Processed	: 8/12/2020 2:49:14 PM

<Chromatogram>

C:\Documents and Settings\User\Desktop\amy\METHODS 2020\AEB-IV-132-2-90%-1.lcd

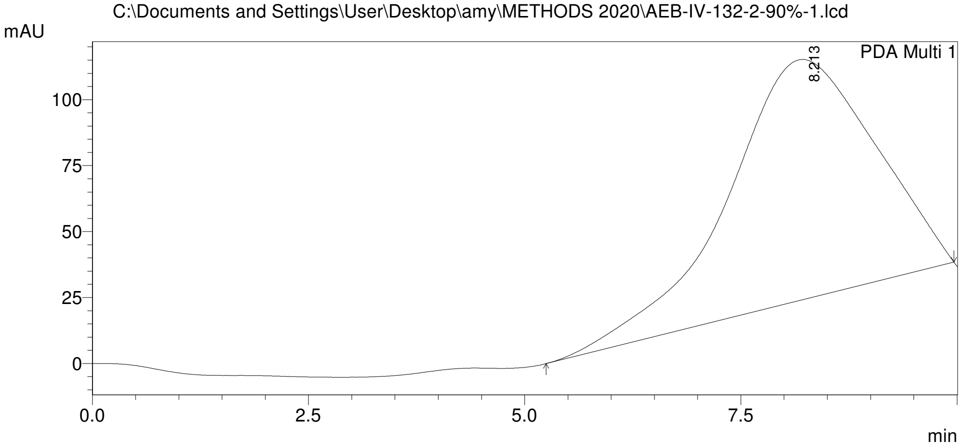

1 PDA Multi 1/254nm 4nm

PeakTable

PDA Ch1 254nm 4nm

Peak#	Ret. Time	Area	Height	Area %	Height %
1	8.213	10720614	91104	100.000	100.000
Total		10720614	91104	100.000	100.000

==== Shimadzu LCsolution Analysis Report ====

C:\Documents and Settings\User\Desktop\amy\METHODS 2020\AEB-IV-146-90%-1.lcd

Acquired by	: Admin
Sample Name	: AEB-IV-146-90%-1
Sample ID	: AEB-IV-146-90%-1
Tray#	: 1
Vail #	: 2
Injection Volume	: 10 uL
Data File Name	: AEB-IV-146-90%-1.lcd
Method File Name	: pos3-90%_10MIN_1_d2.lcm
Batch File Name	: Batch table C3 _90%_10min_1.0_D2.lcb
Report File Name	: Default.lcr
Data Acquired	: 8/11/2020 6:06:47 PM
Data Processed	: 8/11/2020 6:16:49 PM

<Chromatogram>

C:\Documents and Settings\User\Desktop\amy\METHODS 2020\AEB-IV-146-90%-1.lcd

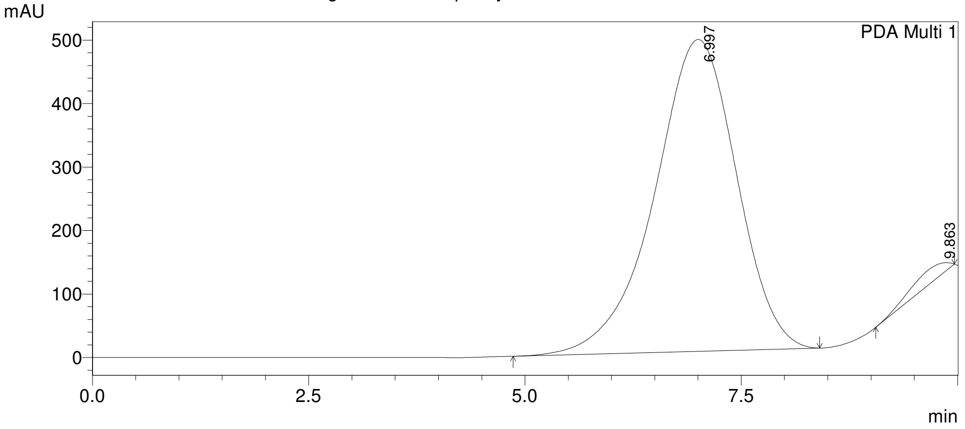

1 PDA Multi 1/254nm 4nm

PeakTable

PDA Ch1 254nm 4nm

Peak#	Ret. Time	Area	Height	Area %	Height %
1	6.997	32401449	491274	97.814	97.371
2	9.863	724000	13263	2.186	2.629
Total		33125450	504537	100.000	100.000

==== Shimadzu LCsolution Analysis Report ====

C:\Users\user\Desktop\amy\AEB-IV-269.lcd

Acquired by	: Admin
Sample Name	: AEB-IV-269
Sample ID	: AEB-IV-269
Tray#	: 1
Vail #	: 2
Injection Volume	: 10 uL
Data File Name	: AEB-IV-269.lcd
Method File Name	: pos3-90%_10MIN_1_d2.lcm
Batch File Name	: Batch table C3 _90%_60min_1.0_D2.lcb
Report File Name	: Default.lcr
Data Acquired	: 1/13/2021 3:48:39 PM
Data Processed	: 1/13/2021 3:58:42 PM

<Chromatogram>

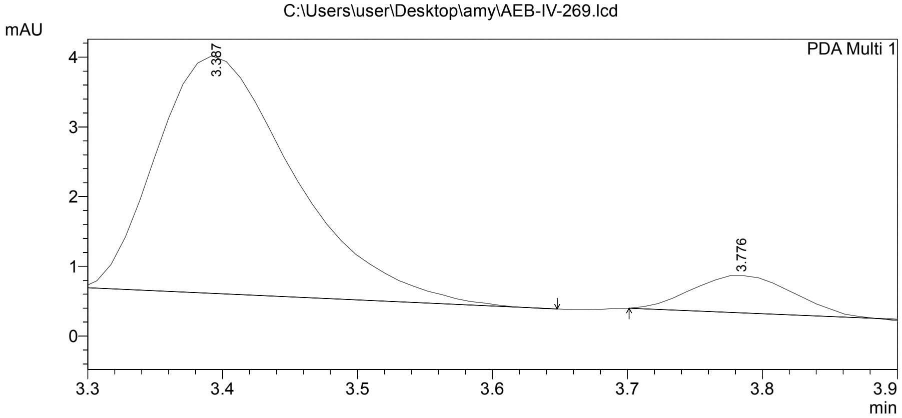

1 PDA Multi 1/254nm 4nm

PeakTable

PDA Ch1 254nm 4nm

Peak#	Ret. Time	Area	Height	Area %	Height %
1	3.387	24003	3403	89.855	86.590
2	3.776	2710	527	10.145	13.410
Total		26713	3930	100.000	100.000

==== Shimadzu LCsolution Analysis Report ====

C:\Users\user\Desktop\amy\AEB-IV-265.lcd

Acquired by : Admin
Sample Name : AEB-IV-265
Sample ID : AEB-IV-265
Tray# : 1
Vail # : 1
Injection Volume : 10 uL
Data File Name : AEB-IV-265.lcd
Method File Name : pos3-90%_60MIN_1.0_D2.lcm
Batch File Name : Batch table C3 _90%_60min_1.0_D2.lcb
Report File Name : Default.lcr
Data Acquired : 1/13/2021 3:12:44 PM
Data Processed : 1/13/2021 3:32:58 PM

<Chromatogram>

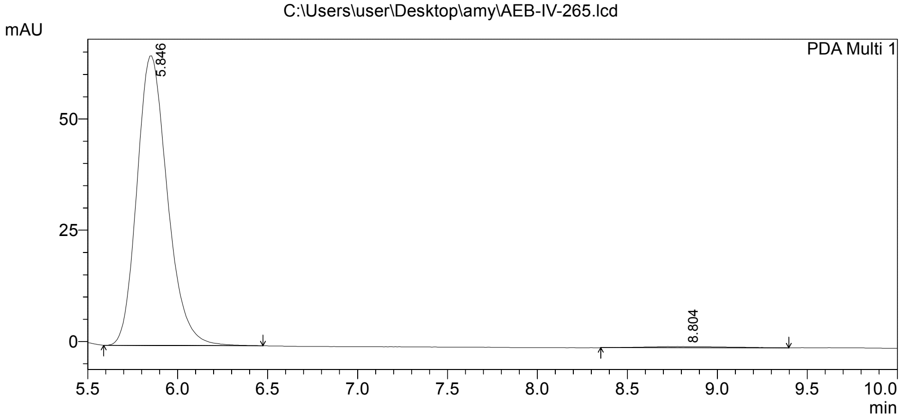

1 PDA Multi 1/254nm 4nm

PeakTable

PDA Ch1 254nm 4nm

Peak#	Ret. Time	Area	Height	Area %	Height %
1	5.846	766612	65056	98.922	99.604
2	8.804	8357	259	1.078	0.396
Total		774969	65314	100.000	100.000

==== Shimadzu LCsolution Analysis Report ====

C:\Users\user\Desktop\amy\AEB-IV-273.lcd

Acquired by	: Admin
Sample Name	: AEB-IV-273
Sample ID	: AEB-IV-273
Tray#	: 1
Vail #	: 3
Injection Volume	: 10 uL
Data File Name	: AEB-IV-273.lcd
Method File Name	: pos3-90%_10MIN_1_d2.lcm
Batch File Name	: Batch table C3 _90%_60min_1.0_D2.lcb
Report File Name	: Default.lcr
Data Acquired	: 1/13/2021 4:09:19 PM
Data Processed	: 1/13/2021 4:19:20 PM

<Chromatogram>

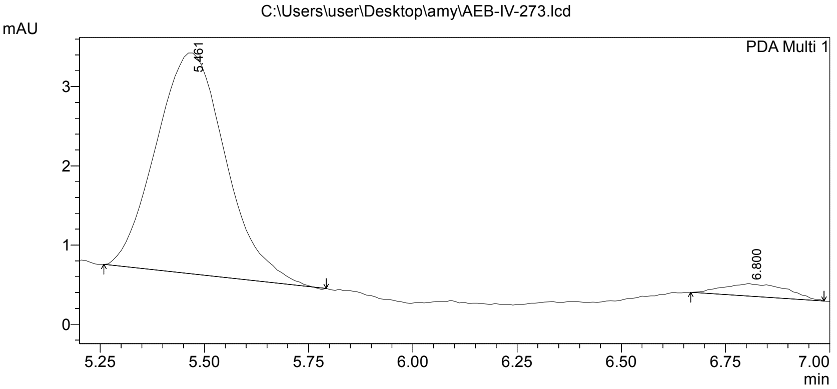

1 PDA Multi 1/254nm 4nm

PeakTable

PDA Ch1 254nm 4nm

Peak#	Ret. Time	Area	Height	Area %	Height %
1	5.461	32272	2781	95.064	94.693
2	6.800	1676	156	4.936	5.307
Total		33948	2937	100.000	100.000

==== Shimadzu LCsolution Analysis Report ====

C:\Users\user\Desktop\amy\AEB-IV-274.lcd

Acquired by	: Admin
Sample Name	: AEB-IV-274
Sample ID	: AEB-IV-274
Tray#	: 1
Vail #	: 4
Injection Volume	: 10 uL
Data File Name	: AEB-IV-274.lcd
Method File Name	: pos3-90%_10MIN_1_d2.lcm
Batch File Name	: Batch table C3 _90%_60min_1.0_D2.lcb
Report File Name	: Default.lcr
Data Acquired	: 1/13/2021 4:29:56 PM
Data Processed	: 1/13/2021 4:39:58 PM

<Chromatogram>

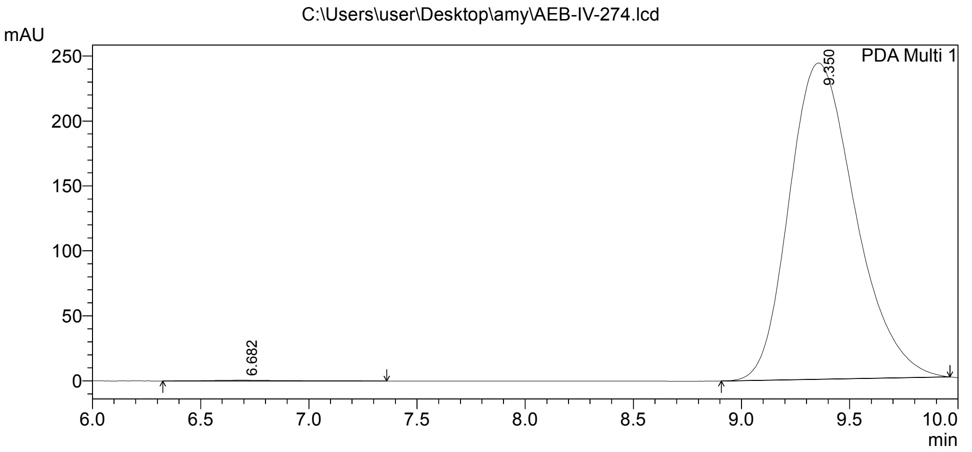

1 PDA Multi 1/254nm 4nm

PeakTable

PDA Ch1 254nm 4nm

Peak#	Ret. Time	Area	Height	Area %	Height %
1	6.682	15641	642	0.301	0.263
2	9.350	5181502	243492	99.699	99.737
Total		5197143	244134	100.000	100.000
